# Design and Manufacturing of Piezoelectric Biomaterials for Bioelectronics and Biomedical Applications

**DOI:** 10.1021/acs.chemrev.5c00399

**Published:** 2025-10-09

**Authors:** Zhuomin Zhang, Zhenqi Wang, Xuemu Li, Yi Zheng, Zhengbao Yang

**Affiliations:** ∇ Department of Mechanical and Aerospace Engineering, Hong Kong University of Science and Technology, Clear Water Bay, Hong Kong SAR 999077, China; ‡ Department of Mechanical Engineering, City University of Hong Kong, Kowloon Tong, Hong Kong SAR 999077, China; § Department of Mechanical Engineering, 6429 Stanford University, Stanford, California 94305, United States

## Abstract

The piezoelectric effect enables the conversion between electrical and mechanical energy, making it essential across various fields. While synthetic piezoelectric ceramics and polymers are extensively utilized in electronics and biomedicine, their inherent rigidity, fragility, processing challenges, toxicity, and nondegradability limit their potential. In contrast, piezoelectric biomaterials offer a promising alternative for biomedical fields because of their natural biocompatibility, biodegradability, and environmental friendliness. However, weak piezoelectricity and challenges in large-scale fabrication hinder their applications. This paper critically reviews recent advances in piezoelectric biomaterials, focusing primarily on design strategies and manufacturing methods. We first summarize the principles, advantages, and categories of a variety of piezoelectric biomaterials. Next, we explore computational studies, highlight emerging approaches in molecular engineering and manufacturing, and examine their cutting-edge applications in bioelectronics and biomedicine. Additionally, we evaluate the effectiveness of various design and manufacturing approaches in enhancing piezoelectric performance, outlining their respective advantages and limitations. Finally, we discuss key challenges and provide insights into computational modeling, fabrication techniques, characterization methods, and biomedical applications to guide future research.

## Graphical abstract

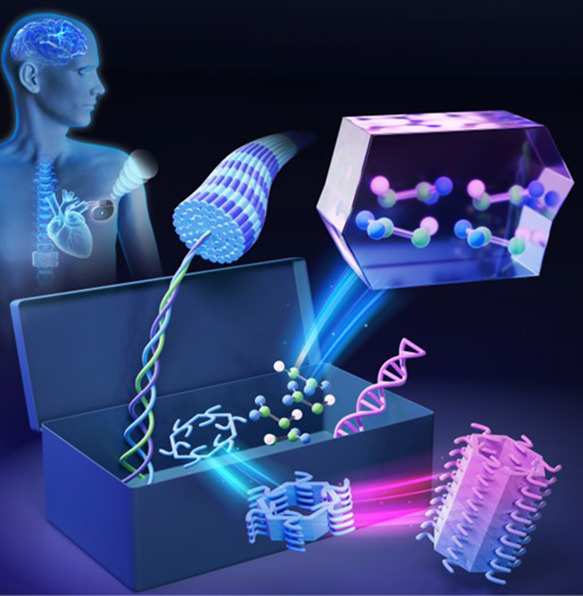

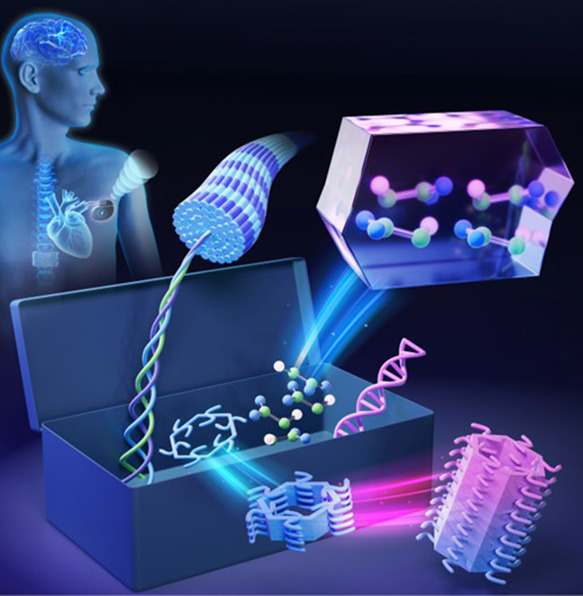

## 1 Introduction

Piezoelectricity is an inherent characteristic of crystals that possess a noncentrosymmetric structure, enabling efficient and precise conversion between electrical and mechanical energies.^1,2^ The history of piezoelectricity dates back to 1880 when the young French scientists Pierre Curie and Jacques Curie discovered and demonstrated the piezoelectric effect in quartz and Rochelle salt, naming it after the Greek term for “pressure electricity”.^3,4^ Since then, extensive research has focused on the fundamentals, fabrication methods, and performance optimization of piezoelectric materials, leading to widespread industrial applications such as ultrasound transducers, ignition devices, resonators, sensors, energy harvesters, and scanning probe microscopes.^5–7^ In recent years, the demand for wearable electronics, implantable devices, and medical healthcare technologies has grown rapidly.^8,9^ As a result, piezoelectric materials, which serve as crucial functional components in modern electronics, have gained widespread attention and use. They are increasingly applied in advanced biomedical fields including health monitoring, disease diagnosis, drug delivery, cancer therapy, tissue regeneration, neuromodulation, and antifouling treatments.^10–12^ However, several limitations associated with conventional piezoelectric materials impede their ability to meet the diverse requirements driven by concerns about biosafety and environmental sustainability. For instance, inorganic materials such as lead zirconate titanate (PZT) contain highly toxic lead elements, and they are rigid, brittle, and difficult to process. Although synthetic polymers such as poly­(vinylidene fluoride) (PVDF) are biocompatible, they still face challenges in terms of degradation within the body or natural environment, potentially leading to unforeseen issues related to biosafety and environmental impact.

Therefore, piezoelectric biomaterials, which either are derived from biological systems themselves or can biodegrade within the body, have recently garnered considerable interest in biomedical applications. These materials exhibit natural flexibility, biocompatibility, and biodegradability. Since the first indication of the piezoelectric effect observed in wool and hair was reported in 1941 by Martin,^13^ piezoelectricity has so far been demonstrated in numerous biomaterials, such as wood,^14,15^ bone,^16,17^ tendons,^18^ invertebrate exoskeletons,^19^ the epidermis,^20^ and viruses.^21^ Of significant excitement is the awarding of the 2021 Nobel Prize in Physiology or Medicine to David Julius and Ardem Patapoutian, whose groundbreaking discovery of the proteins Piezo 1 and Piezo 2 shed light on the mechanisms underlying human sensation, including touch and pain.^22,23^ Their work demonstrated that cells could detect mechanical forces and convert them into electrical signals through electromechanical coupling mediated by Piezo proteins. Similarly, various biological systems exhibiting piezoelectric behavior generate bioelectric signals in response to mechanical deformation, contributing to essential physiological functions. For instance, the piezoelectric charges generated in the human tibia during walking are known to influence bone remodeling and growth,^24,25^ while the piezoelectric potential produced in the lungs during respiration may facilitate oxygen binding to hemoglobin.^26^ Furthermore, the piezoelectric or ferroelectric properties of blood vessel walls may be associated with thrombosis and play a significant role in the progression of atherosclerosis.^27^

Although many biomaterials exhibit a piezoelectric response, very few can be used in engineering. Most research on piezoelectric biomaterials primarily focuses on theoretical analysis and investigations at the micro-nanoscale dimensions with key challenges in scaling up the manufacturing process to create larger structures and the relatively weaker piezoelectricity of these biomaterials compared to ceramics and polymers. These limitations significantly hinder their practical applications. Several excellent reviews have been published, focusing on mechanisms,^28,29^ materials,^30–33^ structure,^10^ potential applications^6,34–37^ and degradation characteristics^38,39^ of piezoelectric nanomaterials and biomaterials. By contrast, this review aims to address the critical challenge of translating molecular design and processing strategies into complex, high-level functional organizations that are suitable for real applications (Figure 1). We systematically summarize the range of discovered and engineered piezoelectric biomaterials, clarify their working principles, and compare their piezoelectric performance. Particular attention is given to emerging computational methods, molecular engineering approaches, and fabrication techniques (mechanical, electrical, magnetic, and thermal) as well as their applications in sensing, actuation, filtration, energy harvesting, and tissue engineering. In addition, we discuss emerging biomedical applications of piezoelectric materials, including neuromodulation, piezocatalytic therapies, and piezocatalytic materials synthesis. Finally, we highlight key challenges in materials, characterization, manufacturing, and applications and propose possible solutions and future directions—including enhancing piezoelectric performance, advancing reliable characterization methods, enabling more complex structures, and achieving seamless integration into bioelectronic and biomedical systems.

**Figure 1:**
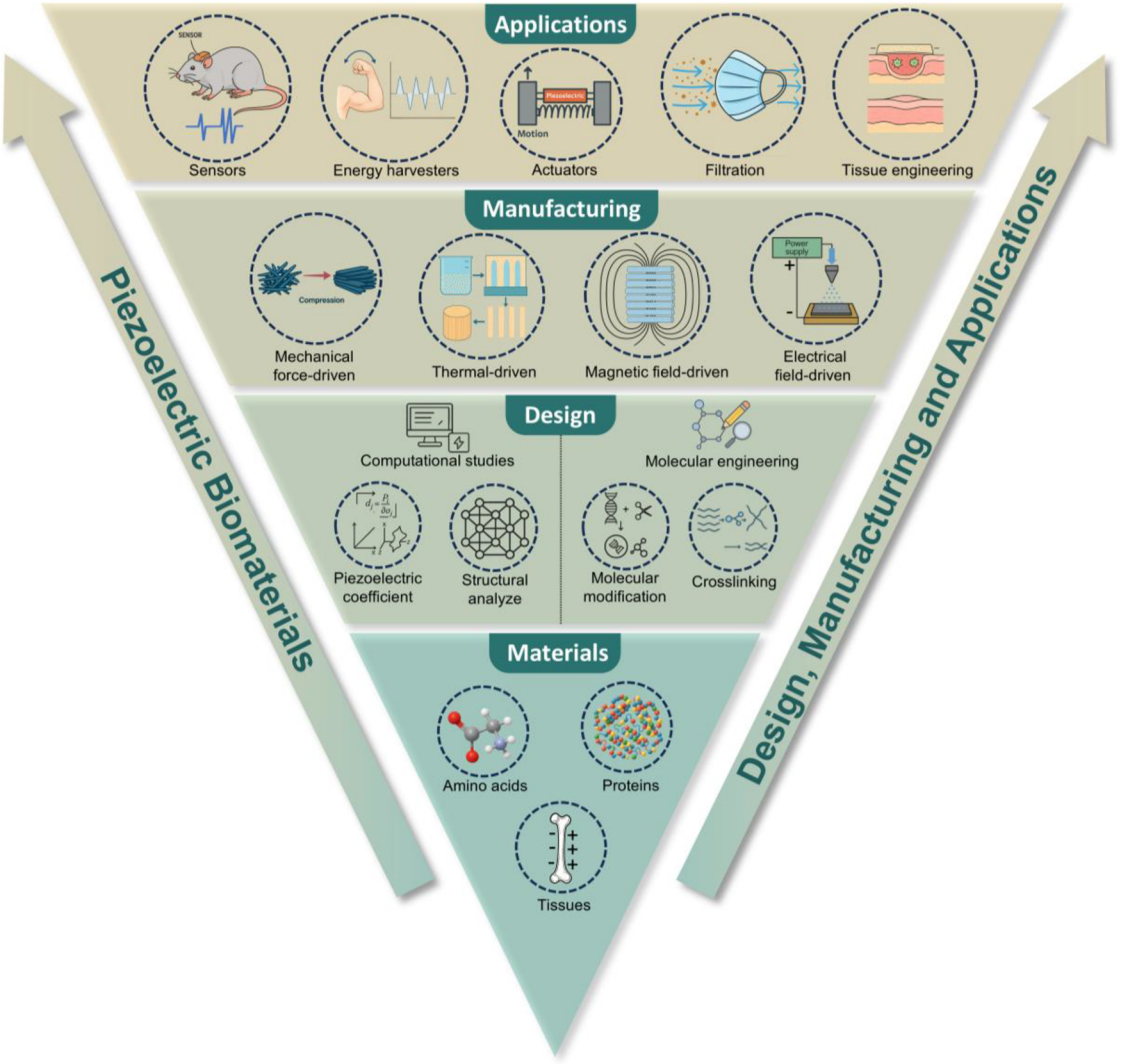
Overview of piezoelectric biomaterials: from materials, to design, to manufacturing and applications.

## 2 Fundamentals of Piezoelectricity

The relationship among dielectric, piezoelectric, pyroelectric, and ferroelectric materials is depicted in Figure 2A. A dielectric is an insulating material that exhibits polarization when subjected to an external electric field. This polarization phenomenon involves the separation of positive and negative charges, resulting in the formation of dipoles. The polarization is quantified as the dipole moment per unit volume. Additionally, dielectrics undergo electrostrictive strain upon applying an electric field, which is the square of the polarization, although this strain is typically small enough to be negligible. However, if a dielectric lacks centrosymmetry, it can exhibit piezoelectric strain under a varying electric field. While this response is often approximated as linear, in practice it frequently involves nonlinear contributions due to domain wall motion and related extrinsic effects.^40^ According to crystallographic theory, only when belonging to 20 non-centrosymmetric point groups can a dielectric be piezoelectric. Within the piezoelectric point groups, there are 10 groups that possess distinctive polar axes and demonstrate spontaneous polarization, leading to the pyroelectric effect, where the spontaneous polarization alters with changes in temperature. Moreover, if this spontaneous polarization can be reversed through the application of an external electric field, then the material is additionally categorized as ferroelectric.

**Figure 2:**
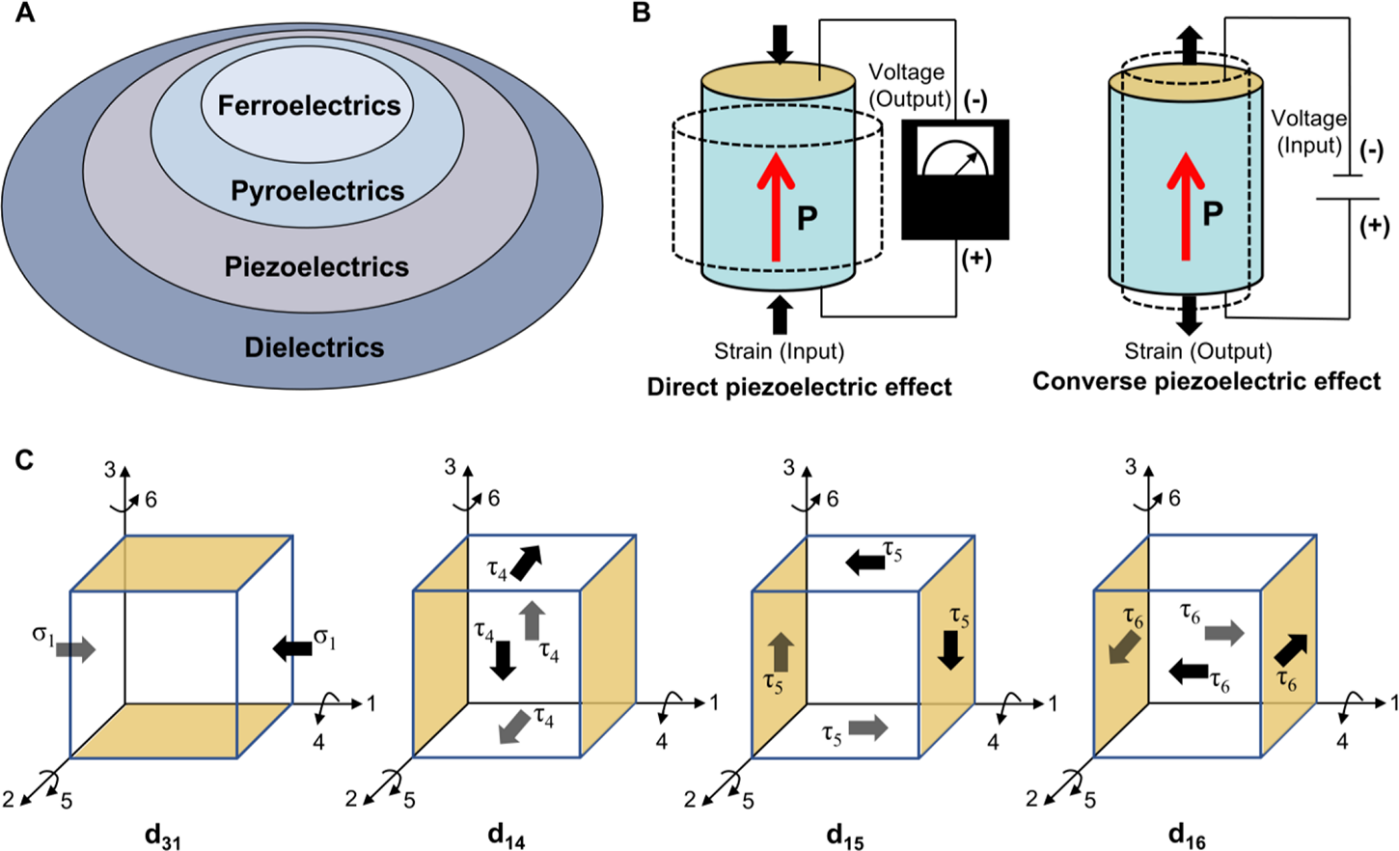
Principles of the piezoelectric effect. A) Illustration of the relationships among dielectric, piezoelectric, pyroelectric, and ferroelectric materials. B) Schematic of the direct piezoelectric effect and converse piezoelectric effect. C) Illustration of the piezoelectric mode for transverse piezoelectric strain coefficient d_31_ and shear piezoelectric strain coefficients d_14_, d_15_, and d_16_, respectively. Yellow shading denotes the electrodes to detect the polarization.

The focus of this review is on piezoelectric materials, which are highly attractive thanks to their low structural prerequisites and widespread presence in biological materials or systems. Piezoelectric materials exhibit both the direct piezoelectric effect and the inverse or converse piezoelectric effect governed by the following equation:^41^

1 $$\left[\begin{array}{l} Direct\\ Converse \end{array}\right]=\left[\begin{array}{l} D\\ S \end{array}\right]=\left[\begin{array}{ll} d & \varepsilon^{T}\\ s^{E} & d^{t} \end{array}\right]\left[\begin{array}{l} T\\ E \end{array}\right]$$

where *S*, *T*, *D*, and *E* represent the strain, stress, charge density, and electric field, respectively, *s* ^*E*^ is the compliance under a constant electrical field, *ε* ^*T*^ is the dielectric permittivity under a constant stress, and *d* and *d* ^*t*^ represent the matrices for the direct and converse piezoelectric effect, with the superscript *t* indicating the transpose operation.

The direct piezoelectric effect enables the conversion of mechanical energy into electrical energy (Figure 2B). When pressure is applied to the surface of a piezoelectric material, it generates electric charges. This effect has found applications in sensors and nanogenerators. On the other hand, the inverse piezoelectric effect allows for the conversion of electrical energy into mechanical energy (Figure 2B). When an electric field is applied, it results in a change in the length of the piezoelectric material. This effect is utilized in actuators and ultrasonic transducers. Figure 2B represents the most classical and commonly used longitudinal piezoelectric coefficient, namely d_33_. In piezoelectric biomaterials, nonlongitudinal transverse or shear piezoelectric coefficients are also very common and play a significant role. For example, β-glycine crystals exhibit a marvelous shear piezoelectric coefficient, d_16_, of about 195 pm V^–1^, comparable to the inorganic ceramics.^42^ Figure 2C illustrates several of the most classical transverse piezoelectric coefficients, d_31_, and shear piezoelectric coefficients, d_14_, d_15_ and d_16_. Here, d_31_ represents the polarization change in the 3-direction when pressure is applied in the 1-direction. d_14_, d_15_ and d_16_ represent the generation of charges on the surface in the 1-direction when shear forces are respectively applied in the 4, 5, and 6 directions. Due to the multidirectional and multimodal nature of mechanical forces in physiological environments, flexible design of piezoelectric material structures can utilize different piezoelectric coefficients to achieve multidirectional electromechanical energy conversion and enhance system efficiency.

## 3 Comparison between Piezoelectric Biomaterials and Synthetic Piezoceramics and Polymers

Inorganic perovskite piezoceramics are currently the most widely used class of piezoelectric materials. Their development began during World War II with the synthesis of barium titanate (BTO), a ceramic with a simple perovskite structure that exhibited piezoelectric properties comparable to those of crystals but with a dielectric constant 100 times higher.^43^ This breakthrough led to the discovery of various piezoelectric perovskite oxides, among which PZT, introduced by Shirane in 1952,^44^ remains the most extensively used piezoelectric material to date. In addition to these inorganic materials, in 1969, Kawai first discovered strong piezoelectricity in the organic polymer PVDF.^45^ Then, more synthetic polymers such as PVDF-copolymers,^46,47^ polyacrylonitrile,^48^ and nylon-11 ^49,50^ were demonstrated with evident piezoelectricity.^51^ Piezoelectricity is also an essential property present in many natural biomaterials and plays a fundamental role in biological systems. It originates from intricate dipolar interactions and hydrogen bonding networks within biomolecular structures that often exhibit self-assembly and hierarchical organization. Owing to their natural origin, piezoelectric biomaterials offer distinct advantages over conventional inorganic and synthetic counterparts, making them especially valuable for applications in biomedicine, transient electronics, and sustainable technologies.

Figure 3 summarizes the key advantages and limitations of piezoceramics, synthetic polymers, and biomaterials. Inorganic piezoceramics, such as PZT and BTO, exhibit strong piezoelectric responses, excellent electromechanical coupling, thermal stability, and long-term reliability, making them well-suited for high-performance and harsh-environment applications. However, their rigidity and brittleness limit their use in flexible systems. Additionally, their manufacturing often requires high energy input, involves toxic components like lead, and results in non-biodegradable waste. Synthetic piezoelectric polymers, such as PVDF, are lightweight, flexible, and biocompatible, which makes them favorable for wearable and biomedical uses. Nonetheless, they are limited by relatively low piezoelectric output, reduced thermal stability, and environmental concerns due to their non-biodegradable nature. Due to the fluorine-containing nature of PVDF, although it is generally considered to have certain biocompatibility, potential environmental and health risks still exist throughout its lifecycle, particularly during the production process and incineration or landfilling after disposal. The European Chemical Agency’s (ECHA) scientific committees for Risk Assessment (RAC) and for Socio-Economic Analysis (SEAC) are currently evaluating the proposal to restrict Per- and Polyfluoroalkyl Substances (PFAS) in the EU/EEA. This highlights concerns regarding the environmental friendliness and sustainability of fluoropolymers like PVDF.^52^

**Figure 3:**
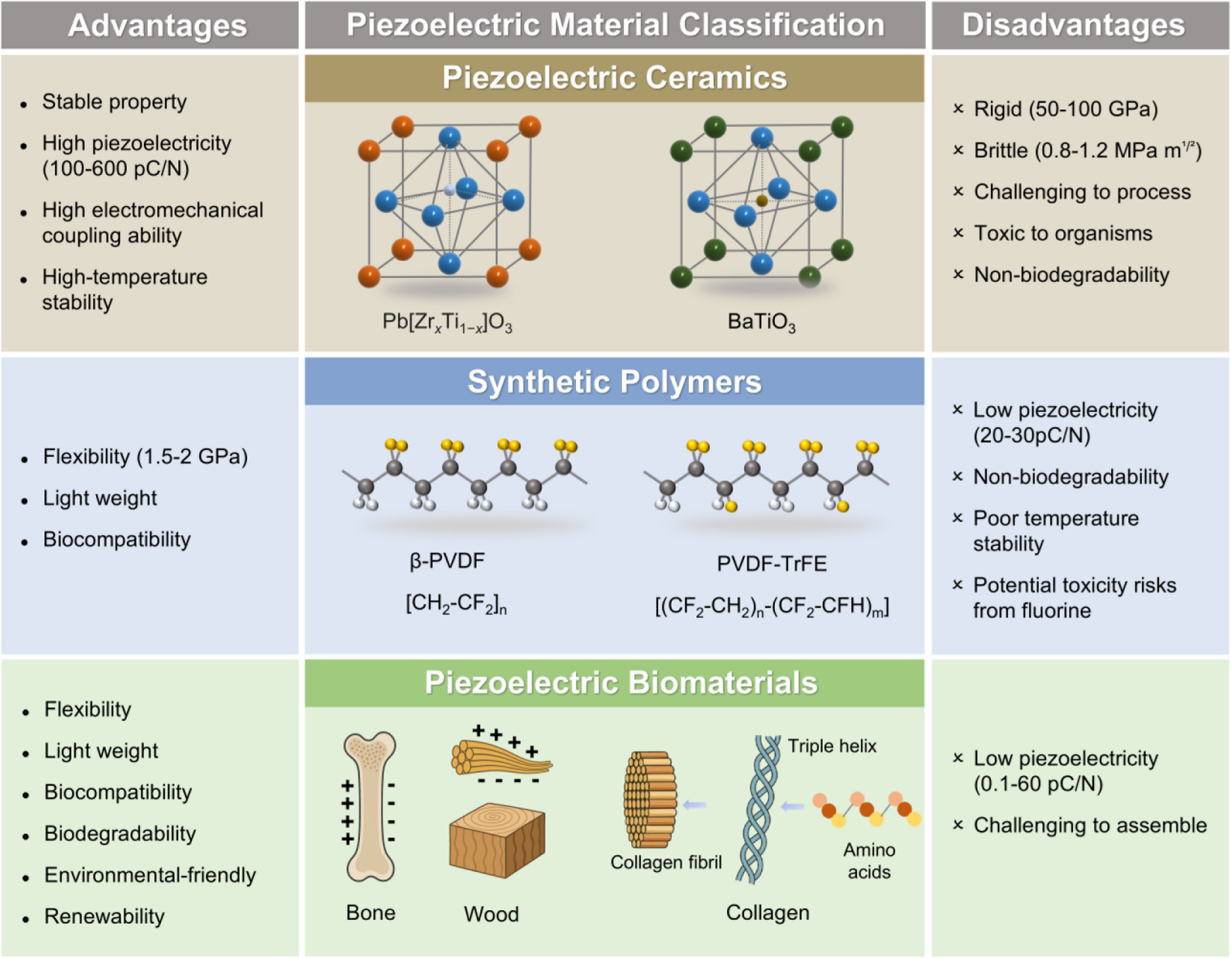
Comparison of piezoelectric biomaterials with synthetic piezoceramics and polymers.

In comparison, piezoelectric biomaterials, while generally exhibiting lower piezoelectric coefficients and facing scalability challenges, offer unique advantages such as intrinsic biocompatibility, biodegradability, and environmental sustainability, which are summarized as follows: **Flexibility:** Many piezoelectric biomaterials are lightweight, soft, and deformable, making them suitable for a wide range of applications, including comfortable sensors, mechanically deformable devices, and conformal biointegrated devices. They can conform to biological tissues and convert biomechanical energy from body movements into electricity through flexible nanogenerators. **Biocompatibility**: These materials are inherently biocompatible, showing a minimal risk of causing clotting, inflammation, or toxicity when implanted. Natural biomaterials inherently meet the requirements of ISO 10993 standards, such as hemocompatibility, cytotoxicity, and sensitization, making them the optimal choice for implantable medical devices. In contrast, inorganic materials such as PZT contain toxic elements, such as lead, posing health risks. **Degradability and resorbability**: A key feature of piezoelectric biomaterials is their ability to degrade in biological environments. They can undergo hydrolysis or enzymatic reactions, with byproducts being safely absorbed and even metabolized by the body. For instance, piezoelectric peptides and proteins break down into useful nutrients. However, some, such as cellulose, may require microbial enzymes for degradation. **Environmental friendliness**: These materials are nontoxic, require minimal harmful chemicals in processing, and degrade naturally, making them easier to dispose of and less polluting. **Sustainability and Renewability**: As carbon-based materials derived from abundant natural sources, piezoelectric biomaterials offer sustainable alternatives. For example, wood and collagen—widely available in nature—both exhibit piezoelectric properties and serve as renewable building blocks for future green electronics.

## 4 Categories and Mechanisms of Piezoelectric Biomaterials

In recent decades, researchers have continuously discovered new biological tissues or biomolecular materials that exhibit piezoelectric properties. In summary, these piezoelectric biomaterials can be classified into amino acids, peptides, proteins and protein-based tissues, polysaccharides and polysaccharide-based tissues, and synthetic biodegradable molecular materials, as illustrated in Figure 4.

**Figure 4:**
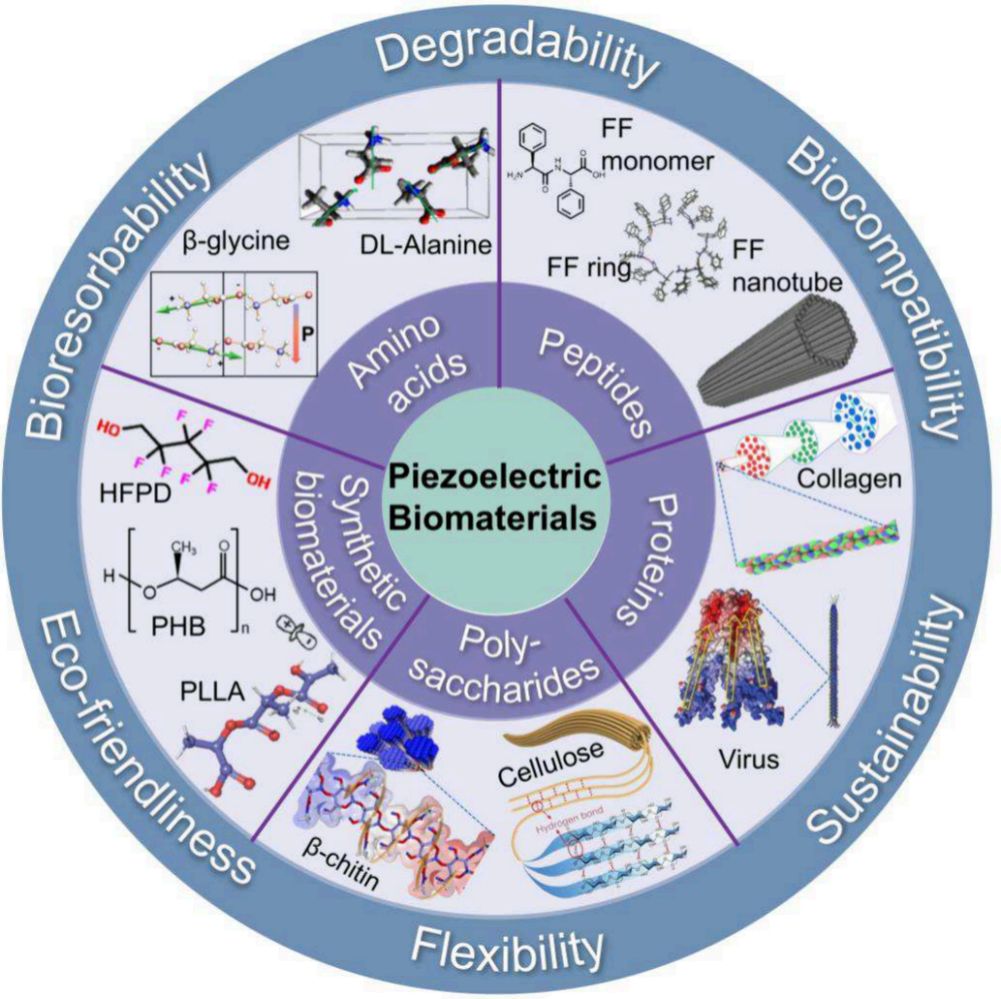
Schematic of the advantages and categories of piezoelectric biomaterials. Reproduced with permission.^53^ Copyright 2023, Springer Nature. Reproduced with permission.^54^ Copyright 2019, American Physical Society. Reproduced with permission.^55^ Copyright 2011, Wiley-VCH. Reproduced with permission.^21^ Copyright 2012, Springer Nature. Reproduced with permission.^56^ Copyright 2018, Springer Nature. Reproduced with permission.^57^ Copyright 2018, Elsevier. Reproduced with permission.^6^ Copyright 2019, Wiley-VCH.

### 4.1 Amino Acids

Amino acids are the fundamental building blocks of proteins such as collagen, silk, and keratin, and their piezoelectric properties arise from the non-centrosymmetric crystalline forms. In 1970, the piezoelectric properties of amino acid crystals with various structures were studied through crystallographic analysis and resonance tests.^58^ Most of these crystals, whether in the left-handed (l) or right-handed (d) form, exhibit piezoelectricity due to the presence of chiral symmetry groups.^59–66^ In 2019, Guerin and Thompson predicted the high longitudinal piezoelectricity of about 10.3 pm V^–1^ in racemic dl-alanine crystal.^54^ They demonstrated that the presence of net molecular chirality is not a mandatory requirement for the manifestation of the piezoelectric effect in bio-organic crystals.^67^

Among amino acids, glycine is the simplest in structure and the only nonchiral amino acid. It exhibits various polymorphic forms, with three different polymorphs, α, β, and γ-glycine, capable of crystallizing under ambient conditions.^68–71^ The crystal structure of α-glycine is classified under centrosymmetric space group P21/c, thereby excluding the presence of piezoelectric properties. Conversely, both β-glycine and γ-glycine crystals belong to non-centrosymmetric point groups, P21 and P32, respectively, and therefore exhibit evident piezoelectric behavior.^53,72–76^ Kholkin pioneered research on piezoelectric glycine, demonstrating through both simulations and experiments that β-glycine and γ-glycine possess both piezoelectricity and ferroelectricity.^77–82^ In 2018, Guerin et al. revealed that β-glycine exhibits an exceptionally large shear piezoelectric strain coefficient (d_16_) of 195 pC N^–1^ and a marvelous piezoelectric voltage coefficient (g_16_) of up to 8 Vm N^–1^, surpassing those of traditional inorganic or organic piezoelectric materials.^42^ Additionally, they also reported a significant longitudinal piezoelectric response in γ-glycine with a d_33_ value of 10.4 pC N^–1^.

### 4.2 Peptides

Peptides are short chains composed of amino acid monomers linked by peptide (amide) bonds.^83^ Similar to amino acids, the piezoelectric properties of peptides can be attributed to the internal electric dipoles formed between the amino and carboxyl groups.^84^ Moreover, peptides exhibit more complex noncovalent interactions, such as hydrogen bonding, π-π stacking interactions, hydrophobic interactions, and electrostatic interactions, due to their larger molecular size and the presence of multiple functional groups compared to amino acids.^85^ These interactions lead to higher-level organized self-assembly and non-centrosymmetric structures. Piezoelectric behavior has been discovered in various peptides, including the oligopeptides such as diphenylalanine (FF),^86^ fluorenylmethoxycarbonyl-diphenylalanine (Fmoc-FF),^87^ cyclo-phenylalanine-tryptophan (cyclo-FW),^88^ cyclo-glycine-tryptophan (cyclo-GW),^89^ bis-cyclic β-peptides,^90^ and synthetic polypeptides such as poly-γ-methyl-l-glutamate (PMLG)^91–93^ and poly-γ-benzyl-l-glutamate (PBLG).^94–97^

FF, one of the most well-known piezoelectric biomolecules, has been extensively studied due to its simple structure, high rigidity, and excellent piezoelectric properties.^98–104^ FF (NH_2_-Phe-Phe-COOH) is a dipeptide composed of two natural phenylalanine (Phe) residues. FF dipeptides can self-assemble into ordered nanotubes through hydrogen bonding and π-π stacking, forming non-centrosymmetric hexagonal crystal structures (C_6_) with inherent piezoelectricity.^105–109^ In 2003, Gazit et al. obtained FF peptide nanotubes for the first time by characterizing the minimal recognition motif of amyloid-β protein associated with Alzheimer’s disease.^110^ In 2010, Kholkin et al. measured the large shear piezoelectric coefficient, d_15_ (60 pm V^–1^), of FF peptide nanotubes using piezoresponse force microscopy (PFM).^86^ In 2016, they employed the PFM technique to measure the complete piezoelectric coefficient matrix of FF peptide nanotubes in different configurations, with d_33_, d_31_, d_15_, and d_14_ values of 18 ± 5 pm V^–1^, 4 ± 1 pm V^–1^, 80 ± 15 pm V^–1^, and 10 ± 1 pm V^–1^, respectively.^103^

### 4.3 Proteins and Protein-Based Tissues

Proteins are complex macromolecules formed by the folding of polypeptide chains, exhibiting a hierarchical spatial structure. Proteins are essential components of all cells and tissues in the human body, serving as the fundamental building blocks of life. There are numerous types of proteins in living organisms, each with distinct properties and functions. Piezoelectricity is also an important electromechanical property found in many proteins, including collagen,^111–115^ silk,^116–119^ keratin,^13,120,121^ prestin,^122–126^ elastin,^27,127–129^ lysozyme,^130–132^ and capsid protein of phage viruses.^21,133–138^

Collagen is the most abundant structural protein found in animal bodies. The structure of collagen consists of three twisted polypeptide chains, forming a triple helix.^139–142^ It is also a source of piezoelectricity in various biological tissues such as bone,^16–18,143–146^ tendons,^18,111,147–149^ sclera,^150^ the small intestine,^151,152^ skin,^20^ and fish swim bladder.^153^ Despite numerous experimental evidences supporting its reliable piezoelectricity, the fundamental principles underlying collagen’s piezoelectric properties are still not fully understood. Various hypotheses have been proposed to elucidate the underlying mechanisms behind the piezoelectricity observed in collagen fibrils, involving non-centrosymmetric structures, the presence of polar bonds at the molecular level, the rotational behavior of CO-NH bonds in the α-helical structure, and the polarization of hydrogen bonds within collagen.^154–157^ In 2016, Zhou et al. conducted a comprehensive investigation into the molecular origin of the “supertwisted” collagen’s piezoelectric effect through full atomistic simulations.^158^ Their findings revealed that collagen exhibits uniaxial polarization aligned with the long axis of collagen fibrils, demonstrating molecular-level piezoelectricity resulting from reorientation and alterations in the magnitude of permanent dipoles induced by mechanical stress. Shear piezoelectric coefficients of collagen fibrils or films reported in different studies varied from 0.1 pm V^–1^ to 12.0 pm V^–1^ for d_14_, and from 1 pm V^–1^ to 6.2 pm V^–1^ for d_15_.^111,149,159–166^

### 4.4 Polysaccharides and Polysaccharide-Based Tissues

Cellulose, which serves as the primary constituent of plant cell walls, is the most prevalent natural polysaccharide found on our planet. Notably, cellulose also showcases the characteristics of piezoelectricity.^167,168^ Cellulose comprises β-glucose units linked by glycosidic bonds, adopting a fibril bundle structure in wood cells. In the 1950s, Fukada et al. first reported cellulose’s piezoelectric properties by measuring the electromechanical response of cut wood.^14,15^ They attributed the piezoelectric properties of wood to the polarity ordering of hydroxyl groups in crystalline cellulose and the monoclinic symmetry of the crystal structure.^169,170^ In the early stages, Fukada’s research findings indicated that solely the shear piezoelectric effect of cellulose within wood tissues could be measured (d_14_ and d_15_, with values only one-twentieth of standard quartz), while no evident longitudinal and transverse effects were examined.^14,171^ They proposed that the crystalline cellulose microfibrils present in the cell walls of wood tissues exhibit uniaxial alignment and are predominantly arranged in an average antiparallel manner.^14,171^ With advancements in wood technology, it became possible to extract cellulose completely from wood, leading to the discovery of two polymorphs, Iα and Iβ, which belong to monoclinic and triclinic crystal structures, respectively, both exhibiting non-centrosymmetry.^172–175^ Subsequently, extensive studies have been conducted on the piezoelectricity of cellulose nanocrystals (CNCs) and their formed nanocrystalline films or paper.^168,169^ Compared with natural wood, they exhibit higher levels of crystallization and a more oriented structure, consequently demonstrating superior piezoelectric coefficients ranging from 0.1 pm V^–1^ to 19.3 pm V^–1^.^176–186^

Chitin, the second most abundant natural polysaccharide after cellulose, is widely distributed in various organisms in nature, such as insects, exoskeletons of crustaceans (crabs, shrimp, lobsters), as well as the cell walls of fungi.^187–189^ Chitin is a polymer composed of long chains of N-acetyl-d-glucosamine units. Chitin can be deacetylated to form poly­(d-glucosamine), commonly known as chitosan, which has widespread industrial use. Both chitin and chitosan exhibit piezoelectric properties, attributed to their non-centrosymmetric molecular structure, much like cellulose. Chitin typically crystallizes into three forms: α, β, and γ. As early as the 1970s, Fukada and colleagues reported that α-chitin displayed relatively low shear piezoelectricity (less than 0.1 pC N^–1^) when subjected to oscillating mechanical stress.^190^ Recently, Kim et al. utilized the PFM technique to examine fabricated chitin films enriched with the β phase and obtained a piezoelectric coefficient of approximately 4 pm V^–1^, consistent with the computational results.^57^

### 4.5 Synthetic Biomaterials

In addition to the natural biomaterials mentioned above, many biodegradable piezoelectric polymers or molecular crystals have been synthesized in the laboratory, offering significant flexibility and multifunctionality in tailoring material properties.

A widely studied synthetic piezoelectric biopolymer is polylactic acid (PLA), produced by the polymerization of lactic acid, a common byproduct of human metabolism. As an FDA-approved, biocompatible, and implantable material, PLA has been broadly utilized in diverse biomedical applications that exploit its piezoelectric properties.^191–197^ The chiral nature of lactic acid, which exists as two enantiomers—l-lactic acid and d-lactic acid—results in two different configurations, namely poly­(l-lactic acid) (PLLA) and poly­(d-lactic acid) (PDLA).^198^ The alignment of dipoles stemming from the carbon–oxygen double bonds (C=O) in the polymer backbone can be expected to generate shear piezoelectricity, with the piezoelectric constants (d_14_) ranging from 5 pm V^–1^ to 9.8 pm V^–1^ reported in different studies.^199^

Poly­(β-hydroxybutyrate) (PHB) is another well-known synthetic piezoelectric biopolymer with excellent biocompatibility and biodegradability. PHB crystals have a high degree of crystallinity and are composed of two twisted left-handed helical molecules that form a double helix structure along the polymer chain.^200,201^ The helical arrangement gives rise to an asymmetric α phase in PHB, which allows it to exhibit shear piezoelectricity (d_14_ = 1.6–2 pm V^–1^).^39,202–204^

Organic single-component molecular crystals mimicking amino acid crystals have also been a promising candidate for biodegradable piezoelectrics because they are generally water-soluble and tend to be absorbed or eliminated from the body. Typical synthetic organic molecular piezoelectrics, such as Croconic acid^205^ and 2-(hydroxymethyl)-2-nitro1,3-propanediol,^206^ exhibit piezoelectric coefficients ranging from 5 pm V^–1^ to 27.8 pm V^–1^. Recently, Xiong and Zhang first demonstrated 2,2,3,3,4,4-hexafluoro-1,5-pentanediol (HFPD) biodegradable molecular crystals with a remarkable piezoelectric strain coefficient d_33_ of 138 pm V^–1^ along with a significant piezoelectric voltage constant, g_33_, reaching around 2,450 mV m N^1–^.^207^

Natural piezoelectric biomaterials, such as collagen, peptides, and amino acid crystals, inherently offer excellent biocompatibility, biodegradability, and low immunogenicity, making them highly attractive for biomedical use. Their structures are naturally optimized for safe integration with tissues, although their piezoelectric output and large-scale processability remain limited. In contrast, synthetic biomaterials generally offer greater processability and tunability and in some cases enhanced piezoelectric performance. PLLA, for example, is FDA-approved, and its biocompatibility and biodegradability have been well demonstrated through years of clinical use, making it a representative synthetic option with established safety. Meanwhile, newly developed small-molecule organic crystals such as HFPD show outstanding piezoelectric constants and potential biodegradability, but their long-term biocompatibility and degradation behavior still require a more systematic investigation. Overall, natural biomaterials excel in safety and degradability, while synthetic systems offer stronger performance and design flexibility. A balanced integration of the two may provide optimal solutions for future biomedical applications

## 5 Computational Studies of Piezoelectric Biomaterials

Over the past decade, significant advancements have been made in the theoretical modeling and computational analysis of piezoelectric biomaterials. These studies help us understand the origins of piezoelectricity in biological piezoelectric materials at the molecular level, offering significant value for identifying potential unexplored piezoelectric materials and refining existing piezoelectric biomaterials.^208^ Current theoretical research on piezoelectric biomaterials primarily relies on methods such as density functional theory (DFT) and molecular dynamics (MD) simulations. DFT is a computational quantum mechanical modeling method used to investigate the electronic structure of many-body systems, particularly atoms, molecules, and solids.^209^ It is widely used in physics, chemistry, and materials science due to its balance of accuracy and computational efficiency. In the field of piezoelectric materials, it has been utilized to predict and rationalize the piezoelectric response of ceramics,^210^ single crystals,^211^ polymers^212^ and two-dimensional materials.^213^ In recent years, numerous researchers have employed DFT to calculate the piezoelectric response of piezoelectric biomaterials, serving as an effective complementary approach to experimental measurements of piezoelectric coefficients.^84^ Furthermore, DFT has been utilized to investigate the underlying mechanisms of piezoelectric biomaterials by computing mechanical parameters, such as elastic modulus, and polarization parameters, such as electric dipoles, thereby providing deeper insights into the origins of their piezoelectric properties.^214,215^ In addition to DFT, MD simulations are commonly used to explore the dynamic behavior of macromolecular systems.^216^ This section highlights representative computational studies on piezoelectric biomaterials organized into four categories: piezoelectric matrix calculation, computation-guided material design, high-throughput screening, and analysis of intermolecular interactions.

### 5.1 Piezoelectric Coefficient Calculations

Given the prevalence of high shear strains in biological piezoelectric materials, using these theoretical approaches to predict full piezoelectric tensor coefficients also facilitates the design of diverse self-assembly strategies during practical fabrication processes to achieve enhanced macroscopic outputs. As the fundamental building blocks of proteins, studying and predicting the piezoelectricity of amino acids contribute to understanding other biological piezoelectric materials. Several studies have utilized DFT to predict the piezoelectricity of amino acid single crystals, calculating piezoelectric constants and validating them through experimental approaches. Guerin et al. performed DFT calculations on three glycine polymorphs (α, β, γ) to determine their elastic and piezoelectric coefficients.^42^ β-Glycine displayed a high transverse shear coefficient (d_16_) of 195 pm V^–1^, resulting from lowered shear stiffness due to its molecular packing (Figure 5A). Similar methods have also been applied to study other amino acids and small molecules analogous to amino acids, such as l-leucine, l-alanine and dl-alanine.^42,217^

**Figure 5:**
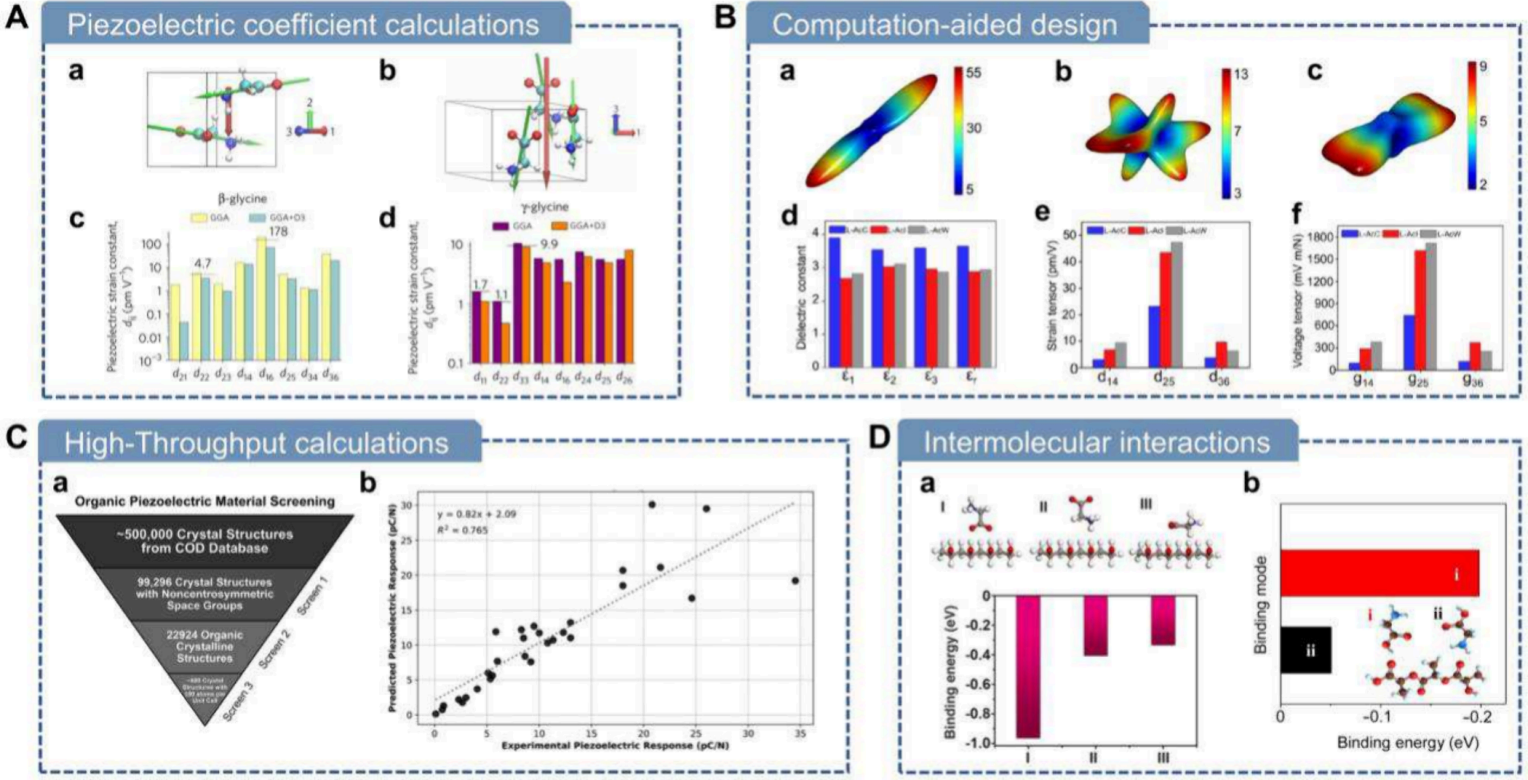
Computational studies of piezoelectric biomaterials. A) Piezoelectric coefficient calculation of glycine. (a) Molecular structure and dipoles of β-glycine. (b) Molecular structure and dipoles of γ-glycine. (c) Calculated piezoelectric strain constants for β-glycine. (d) Calculated piezoelectric strain constants for γ-glycine. Reproduced with permission.^42^ Copyright 2018, Springer Nature. B) Computation-aided design. (a–c) Young’s modulus (GPa) of (a) L-AcC, (b) L-AcI, and (c) L-AcW in 3D. (d–f) Calculated (d) dielectric constant, (e) strain tensor, and (f) voltage tensor of l-AcC, l-AcI, and l-AcW. Reproduced with permission.^63^ Copyright 2023, American Chemical Society. C) Hign-throughput calculations of piezoelectric biomaterials. (a) Overview of the computational screening process for organic piezoelectric materials. (b) Regression plot comparing calculated piezoelectric constants with experimental data reported in the literature. Reproduced with permission.^218^ Copyright 2025, Wiley-VCH. D) Intermolecular interaction calculations. (a) DFT-calculated binding energies for the three binding modes of the glycine-PVA film. Reproduced with permission.^74^ Copyright 2021, American Association for the Advancement of Science. (b) DFT-calculated binding energies for the two binding situations of the glycine-PLLA nanofibers. Reproduced with permission.^219^ Copyright 2024, American Association for the Advancement of Science.

### 5.2 Computation-Aided Design

In addition to calculating and predicting the piezoelectric parameters of existing amino acids, DFT can also serve as an auxiliary tool to guide the supramolecular engineering of biomolecular assemblies. Wang et al. used DFT calculations to validate the enhanced piezoelectric properties of their proposed acetylation modification (Figure 5B),^63^ and Yuan et al. investigated the mechanical and piezoelectric properties of sulfonic acid-containing bio-organic molecules using DFT methods.^220^ Computation-aided design has also been utilized to enhance piezoelectric performance by strategies such as tuning the hydrogen bond interactions between amino and carboxyl groups and modifying the chirality of amino acid single crystals and cocrystals to achieve robust piezoelectric responses.^221,222^

At higher levels of hierarchical biomolecular organization, researchers have employed DFT to investigate the piezoelectric behavior of peptide assemblies. For instance, Basavalingappa et al. calculated the binding energies between molecules in the dipeptides to confirm that the introduction of aromatic groups significantly enhanced the material’s stiffness·^223^ DFT calculations predicted a dipole moment of 2.1 D, suggesting the potential for a high d_33_ value. Ji et al. employed DFT calculations to analyze the significant piezoelectric properties of 4,4′-bipyridine (4,4′-Bpy) and confirm the transition in molecular stacking modes.^224^

As structural complexity increases to the level of proteins and biopolymers, DFT-based methods face limitations in quantitatively analyzing elastic and piezoelectric constants. In such cases, DFT and other computational approaches are more frequently employed for auxiliary nonqualitative analyses or indirect investigations through the disassembly of biomacromolecules. Kim et al. used DFT as an auxiliary tool to analyze the origin of piezoelectricity in chitin. The computational results revealed that the net polarization in β-conformation chitin crystals exhibits strong uniaxial characteristics, whereas the α-phase demonstrates almost negligible theoretical polarization.^57^ Zhou et al. used molecular dynamics simulations to elucidate the mechanistic underpinnings of experimentally observed “supertwisted” collagen architectures.^158^ Guerin et al. employed DFT to further investigate the piezoelectric tensors of collagen’s non-glycine building blocks: hydroxyproline, proline, and alanine.^156^ The results demonstrated that the piezoelectric response of biopolymer structures depends significantly on both the magnitude and the sign of the piezoelectric charge constants of their constituent amino acids. Furthermore, Bera et al. utilized collagen building blocks to develop a piezoelectric generator based on tripeptides (Pro-Phe-Phe and Hyp-Phe-Phe) with the assistance of computational methods

### 5.3 High-Throughput Calculations

Traditional methods in material design face issues of low efficiency. With the advancement of computational technology, high-throughput screening has seen widespread application and development in the field of material design in the last 20 years.^225^ High-throughput calculation is a technique that enables rapid execution of numerous simulations or computations in parallel, allowing researchers to efficiently analyze large data sets or explore many scenarios simultaneously. Recently, Vishnoi et al. presented a high-throughput computational screening of ∼600 non-centrosymmetric organic molecular crystals to identify sustainable piezoelectric materials (Figure 5C).^218^ Using DFT, they created CrystalDFT, a database of electromechanical properties for crystals, from the Crystallographic Open Database. The automated workflow validated predictions against experimental data (R^2^ = 0.76), identifying 22 crystals with longitudinal piezoelectric coefficients (d_33_) up to 79.4 pC/N, surpassing materials like zinc oxide. Low dielectric constants (ε < 5) enhance energy harvesting efficiency. Functional groups like hydroxyl and phenyl derivatives drive strong piezoelectricity. This scalable methodology supports future material discovery for eco-friendly sensors and biomedical devices.

In recent years, the rapid development of artificial intelligence has transformed the fundamental paradigms of material design. Unlike the traditional forward design approach, which involves screening existing materials based on requirements, inverse design—starting directly from target properties—is advancing rapidly.^226–229^ Compared with screening methods that are fundamentally limited by the number of known materials, inverse design can more efficiently produce material structures that meet the needs of piezoelectric material applications. Although AI-assisted material design has not yet been widely studied in the field of piezoelectric biomaterials, the continuous advancement of artificial intelligence and its integration with materials science will open up endless possibilities for this field.

### 5.4 Intermolecular Interactions Calculation

Theoretical computational tools have also been employed as auxiliary methods to study the interactions between piezoelectric biomaterials and other composite materials. Yang et al. employed DFT to analyze the interaction between poly­(vinyl alcohol) (PVA) and γ-glycine in their fabricated wafer-scale bio-organic films. The DFT calculations revealed that PVA macroscopically guides the stacking of glycine molecules, directing the formation of the piezoelectric γ-phase.^74^ Li et al. demonstrated through combined MD and DFT analyses that the C=O bonds on PLLA preferentially form bonds with the -OH groups on Gly (Figure 5D).^219^ This interaction can guide and anchor the orientation of C=O groups on PLLA chains, thereby stabilizing the β-phase and its macroscopic alignment and ultimately leading to enhanced piezoelectric performance.

In summary, computational methods such as DFT and MD have provided valuable insights into piezoelectric biomaterials, from calculating coefficients and guiding supramolecular design to enabling high-throughput screening of new candidates. These approaches have significantly advanced our understanding of the molecular origins of piezoelectricity and facilitated the rational design of next-generation materials. Nonetheless, current computational studies still function largely as complementary tools to experiments, with limitations in handling structural complexity and dynamic biological environments. Looking ahead, the integration of advanced computational techniques with artificial intelligence and data-driven inverse design is expected to accelerate the discovery and optimization of biocompatible high-performance piezoelectric biomaterials.

## 6 Molecular Engineering of Piezoelectric Biomaterials

Molecular engineering utilizes molecular properties, behaviors, and interactions to assemble desired materials and achieve specific functionalities. This approach involves directly modifying molecular structures to influence the characteristics of bottom-up organized macroscopic systems. Molecular engineering plays a vital role in designing and fabricating piezoelectric biomaterials. It often employs chemical design, coassembly, genetic and molecular modification, interface hydrogen bonding, and chemical cross-linking to adjust their polarity, structure, mechanical properties, crystal phase, and orientation, thereby enhancing piezoelectric performance and stability. Those engineered biomolecules are typically further microfabricated into macroscopic self-assembled structures by using simple recrystallization, drop-casting, or solution-casting techniques.

### 6.1 Hydrogen/Fluorine (H/F) Substitution

Based on ferroelectrochemical principles, the H/F substitution strategy enables tuning of key ferroelectric properties such as Curie temperature, spontaneous polarization, and coercive field, while also enhancing piezoelectric performance through the incorporation of long, highly polarized C–F bonds.^230,231^ Therefore, by employing the H/F substitution strategy and crystal engineering, Xiong and Zhang, for the first time, discovered a biodegradable piezoelectric molecular crystal called HFPD, which exhibited a considerable d_33_ value of approximately 138 pC N^–1^ and a piezoelectric voltage constant g_33_ of about 2450 × 10^–3^ Vm N^–1^ without any poling conditions (Figure 6A).^207^ HFPD crystals mimic the β-phase structure of PVDF using just three -CF_2_- units, combined with hydrogen bonding to form infinite chain-like structures. By reducing PVDF’s repeating units to a small molecule, they achieved a 4-fold increase in piezoelectricity. The high d_33_ value is attributed to strong Young’s modulus anisotropy from the ordered 2D hydrogen bond network and aligned F atoms, as well as a pressure-induced phase transition that causes ∼45° molecular rotation, further boosting d_33_. These crystals are biodegradable and soluble in various solvents due to terminal O–H···O hydrogen bonds. In addition, flexible, uniform HFPD–PVA composite films were prepared via solution casting, achieving a d_33_ of 34.3 pC N^–1^. Similarly, the same team investigated the molecular crystal of 1H,1H,9H,9H-perfluoro-1,9-nonanediol (PFND), which contains only seven -CF_2_- groups.^232^ This crystal also exhibited notable piezoelectric (longitudinal d_33_ = 5.7 pC N^–1^) and ferroelectric properties.

**Figure 6:**
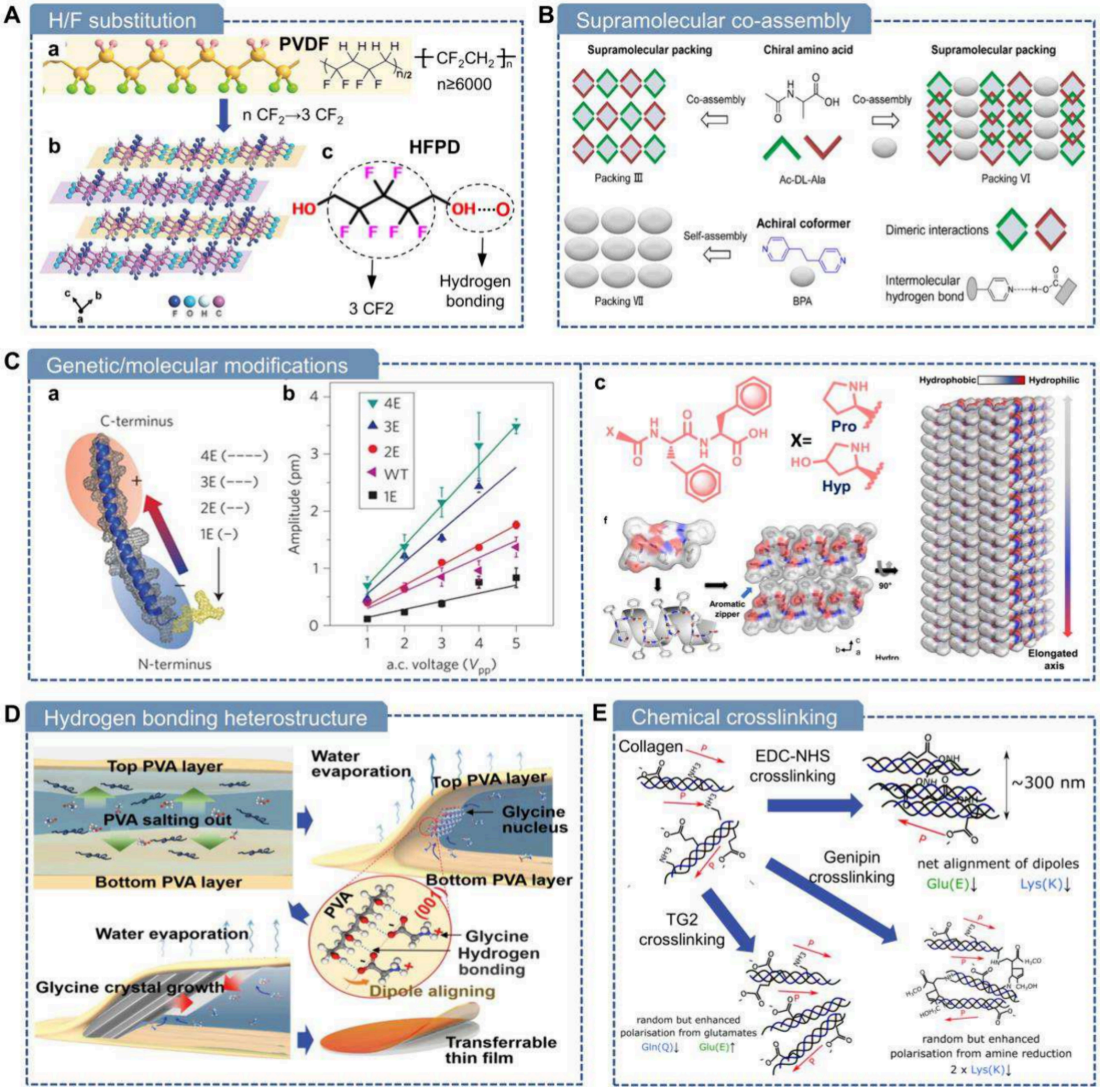
Molecular engineering of piezoelectric biomaterials. A) H/F substitution strategy for HFPD molecular crystal design. (a) Molecular structure of PVDF. Reproduced with permission.^230,231^ Copyright 2022, Wiley-VCH. (b) Packing view of 2D hydrogen bond layers of HFPD crystals along the direction of c-axes. (c) Molecular structure of HFPD. Reproduced with permission.^207^ Copyright 2024, American Association for the Advancement of Science. B) Supramolecular coassembly of centrosymmetric-crystallizing conformers with N-terminally capped alanine-based assemblies (Ac-Ala). Reproduced with permission.^62^ Copyright 2022, American Chemical Society. C) Genetic and molecular modifications of piezoelectric biomaterials. (a) Schematic of negatively charged amino acid glutamates modified M13 bacteriophage and (b) comparison of their piezoelectric responses. Reproduced with permission.^21^ Copyright 2012, Springer Nature. (c) Molecular structure and collagen-like assembly of Hyp-Phe-Phe short peptides. Reproduced with permission.^234^ Copyright 2021, Springer Nature. D) Schematic of the nucleation and crystallization process of heterostructured PVA-γ-glycine bio-organic films facilitated by the interface hydrogen bonding. Reproduced with permission.^74^ Copyright 2021, American Association for the Advancement of Science. E) Schematic of collagen cross-linked via three distinct cross-linkers. Reproduced with permission.^235^ Copyright 2019, Royal Society of Chemistry.

Hu et al. demonstrated that simple fluorination of amino acid side chains can effectively modulate supramolecular self-assembly and significantly enhance piezoelectric performance.^233^ They synthesized three phenylalanine derivatives (Cbz-Phe, Cbz-Phe­(4F), and Cbz-pentafluoro-Phe) and systematically studied their structural and functional properties. While Cbz-Phe and Cbz-pentafluoro-Phe primarily formed amorphous aggregates, Cbz-Phe­(4F) self-assembled into well-ordered single crystals with a C2 space group. Notably, these crystals exhibited a remarkably high piezoelectric coefficient (d_33_ = 17.9 pm V^–1^). Molecular dynamics simulations confirmed that the distinct molecular structure of Cbz-Phe­(4F) plays a critical role in directing its crystallization and piezoelectric behavior.

Overall, due to the strong electronegativity of F and the high polarity of the C–F bond, as well as the presence of many highly piezoelectric fluorinated polymers, introducing F into piezoelectric biomaterials represents a straightforward and effective strategy to enhance piezoelectricity. Nevertheless, the widespread use of fluorinated systems also raises concerns regarding their biocompatibility and long-term biodegradability. Future studies are required to systematically evaluate the in vivo safety, metabolic fate, and environmental impact of fluorinated biomaterials to ensure their sustainable application in biomedical contexts.

### 6.2 Supramolecular Coassembly

Racemic assembly, by mixing equal amounts of left- and right-handed enantiomers, is a common coassembly form. dl-Alanine, a typical racemic amino acid, was demonstrated to have a d_33_ value of 10.3 pC N^–1^ and an exceptionally high g_33_ value of 0.47 Vm N^–1^, surpassing both the longitudinal and shear piezoelectric constants of the l enantiomer.^54^ Kholkin et al. reported the coassembly of layered racemic FF crystals, coassembled by the l,l- and d,d-enantiomers of FF, demonstrating improved thermal and chemical stability, as well as enhanced piezoelectricity (d_33_ = 20 pm V^–1^) comparable to hexagonal FF nanotubes.^236^ However, racemic coassembly does not always enhance piezoelectric properties. Recently, Zhang et al. reported the racemic assembly of l-tyrosine and d-tyrosine and observed a weakening of the optical properties, mechanical rigidity, and piezoelectric output of these racemic assemblies.^67^ This can be attributed to the transformation of the antiparallel β-folded secondary structure in enantiomeric assemblies to a parallel fold due to racemic coassembly, resulting in increased degrees of freedom and consequent changes in physicochemical properties.

Besides the racemic assembly, Gazit et al. reported the entropy-driven coassembly of l-histidine (l-His) with aromatic amino acids, such as Phe, tryptophan (Trp), and tyrosine (Tyr) in both enantiomeric forms.^64^ Compared to the original l-His, coassembled structures exhibited unique morphologies when combined with different aromatic amino acids, including fibrous, rod-like, and sheet-like structures. In particular, the combination of 1-His and 1-Phe amino acids formed a cocrystal, displaying a non-centrosymmetric crystalline structure, indicating potential piezoelectric behavior.^64^ Yuan et al. achieved coassembly of FW-FF, cyclo-FW-FF, and Fmoc-FF-FF crystals.^237^ They observed that the introduction of FW into the coassembled structures resulted in a reduction of interplanar spacing and enhanced non-centrosymmetry, effectively improving the piezoelectric output. The most significant enhancement was observed in the 20% FW-FF sample, which exhibited an effective piezoelectric coefficient d_33_ of 35.5 pm V^–1^, 38% higher than that of pure FF peptides.^237^

Ji et al. introduced a novel approach using centrosymmetric-crystallizing coformer-assisted coassembly to enhance the piezoelectric properties of single crystals (Figure 6B).^62^ Specifically, they manipulated the supramolecular packing of N-terminally capped alanine-based assemblies (Ac-Ala) by altering the chirality of the amino acids and incorporating a nonchiral bipyridine derivative (BPA). Despite the centrosymmetric nature of BPA crystals, which typically excludes piezoelectricity, a significant enhancement in piezoelectric response was achieved when cocrystallized with Ac-l-Ala and Ac-d-Ala, with piezoelectric constants d_14_ of 26.3 and 21.9 pC N^–1^, respectively.^62^ This improvement can be attributed to the enhanced polarization resulting from the supramolecular stacking in the coassembled structures.

Chen et al. utilized guest–host interactions to regulate the conformation-dependent piezoelectric behavior of peptide-based metal–organic frameworks (MOFs) derived from endogenous carnosine dipeptide.^238^ They demonstrated that the oriented arrangement of guest molecules within the host carnosine-Zn­(II) peptide-MOF channels modulated the macroscopic piezoelectricity of the supramolecular assemblies. In particular, the introduction of the guest molecule MeCN resulted in the formation of the lowest symmetric piezoelectric peptide-MOF crystals (space group P1), leading to improved piezoelectric performance with an effective d_33_ of 4.7 pm V^–1^. The resulting nanogenerator could generate an open-circuit voltage (OCV) of 1.4 V.^238^

Supramolecular coassembly offers effective routes to modulate symmetry, polarization, and piezoelectric output by combining enantiomers, aromatic residues, nonchiral coformers, or guest–host systems. These strategies can enhance stability, non-centrosymmetry, and functional performance. A deeper mechanistic understanding is still needed to guide rational design toward reliable and high-performance piezoelectric biomaterials, and the changes in biocompatibility and degradability after coassembly also warrant careful evaluation.

### 6.3 Genetic and Molecular Modifications

The application of genetic engineering to bacteriophage viruses has successfully demonstrated their ability to enhance piezoelectricity by manipulating charge distribution and polarity strength. The intrinsic piezoelectricity of the M13 bacteriophage originates from the absence of inversion symmetry within the pVIII coat protein.^21^ Lee et al. employed recombinant DNA techniques to modify the pVIII coat protein, introducing a varying number of negatively charged amino acid glutamates (E) at the N-terminus of pVIII (Figure 6C-a).^21^ The genetically modified phages exhibited a molecular structure-dependent piezoelectricity. The vertical PFM results demonstrated that as the negative charge E increased, the piezoelectric response correspondingly increased. The effective piezoelectric coefficient d_eff_ increased from its lowest value of 0.14 pm V^–1^ in the 1E-phage to its highest value of 0.70 pm V^–1^ in the 4E-phage (Figure 6C-b).^21^

Genetic engineering of the M13 bacteriophage can also be employed to control the unidirectional polarization alignment during self-assembly. In addition to the major coat protein (pVIII) copies covering the phage body, five minor coat protein (pIII and pIX) copies are distributed at each end of the M13 phage. Lee et al. modified the N-terminus of the minor coat protein (pIII) by introducing a hexa-histidine (6H).^134^ The introduction of 6H resulted in a strong binding interaction with a substrate modified with nickel-nitrilotriacetic acid (Ni-NTA), facilitating the organized assembly of the phage with unidirectional polarization. The resulting d_eff_ value was measured to be 13.2 pm V^–1^, three times higher than that of phage films.^21,134^

Utilizing various functional groups to modify biomolecules, such as amino acids and peptides, is a practical approach for enhancing piezoelectricity, mechanical performance, and stability. Basavalingappa et al. developed a series of phenyl-rich dipeptides, namely β,β-diphenyl-Ala-OH (Dip)-Dip, cyclo-Dip-Dip, and *tert*-butoxycarbonyl (Boc)-Dip-Dip, by substituting each phenylalanine amino acid in the FF, cyclo-FF, and Boc-FF motifs with Dip residues.^223^ These modified dipeptides exhibited a doubling in aromatic moieties, significantly increasing the Young’s modulus to 70 GPa. Notably, due to the denser aromatic network and a relatively small unit cell, Boc-Dip-Dip possessed significant molecular dipoles and an increased asymmetry, exhibiting a greatly enhanced piezoelectric coefficient, d_33_, of 73.1 ± 13.1 pm V^–1^.^223^

Similarly, Fmoc-FF nanofiber networks, obtained by incorporating the Fmoc group as a side chain onto the FF peptide chain, exhibited a shear piezoelectricity of d_15_ = 1.7 pm V^–1^, along with a mechanical modulus comparable to that of biological gels.^87^ Furthermore, cyclic-GW peptides were predicted with a high piezoelectric coefficient d_36_ of 14.1 pm V^–1^ through DFT calculations.^89^ Wang et al. investigated the piezoelectric behavior changes of chemically modified amino acids through acetylation.^63^ They found that the acetylation of amino acid side chains leads to significantly different solid-state packing modes, influencing supramolecular dipoles and structural symmetry. The shear piezoelectric coefficient of the acetylated tryptophan (L-AcW) crystal was significantly enhanced, with a predicted value of d_25_ = 47.3 pm V^–1^.^63^ Bera et al. modulated the piezoelectric behavior in collagen-mimicking short peptides through side chain engineering (Figure 6C-c).^234^ By incorporating both the hydroxyproline (Hyp) and aromatic Phe moieties in the sequence, they developed Phe-Phe-derived short peptides that exhibited helical-like sheet assembly. Among them, Hyp-Phe-Phe showed a higher piezoelectricity, with a d_33_ of 4 pm V^–1^ and d_34_ of 16 pm V^–1^, attributed to the enhanced polarizability under stress induced by hydroxylation.^234^

Genetic and molecular modifications can effectively enhance piezoelectricity by tuning the charge distribution, dipole alignment, and supramolecular packing. These strategies improve performance and stability, but their biocompatibility and scalability still require further investigation. Emerging gene-editing tools such as Clustered Regularly Interspaced Short Palindromic Repeats (CRISPR) may provide new opportunities to precisely engineer protein sequences and supramolecular assemblies, enabling customized charge distributions and dipole alignments. Such approaches could open new directions for the rational design of next-generation piezoelectric biomaterials.

### 6.4 Hydrogen Bonding Heterostructure

Hydrogen bonding serves as a driving force for the self-assembly of supramolecular structures in many biological molecules. In addition, interface hydrogen bonding in the heterogeneous structures also holds the potential for microfabricating piezoelectric biomaterials and increasing their piezoelectricity by means of controlling crystalline phases, enhancing supramolecular dipoles, and facilitating polarization orientation.

Yang and Li et al. fabricated sandwich-structured PVA-glycine-PVA films with well-defined crystal orientation using interfacial hydrogen bonding (Figure 6D).^74^ The structure was formed by sequential precipitation of PVA and glycine from a mixed solution during solvent evaporation. PVA, being less soluble, first precipitated at the water–air and water–solid interfaces, followed by glycine nucleation near the liquid edge, encapsulated by the top PVA layer. Hydrogen bonding at the PVA–glycine interface promoted γ-phase glycine nucleation with polarization perpendicular to the liquid edge. As the glycine-to-PVA ratio increased from 0.5:1 to 2:1, the dominant orientation of the glycine crystals shifted from [110] to [101], enhancing out-of-plane (OOP) piezoelectricity, since the γ-glycine [001] direction is better aligned with [101]. The films showed a high and uniform d_33_ value of 5.3 pm V^–1^ and good flexibility due to the PVA layers. To enhance piezoelectricity in flexible glycine–polymer composite films, Li et al. studied the effects of various water-soluble biodegradable polymers (e.g., PEO, sodium hyaluronic acid, Pullulan, Gelatin, and PVA) and film-forming interfaces with different wettabilities on glycine crystal structure and piezoelectric properties.^239,240^ They found that γ-glycine–PEO films with a sandwich structure, formed on a more hydrophobic PTFE surface, achieved a higher OOP piezoelectric coefficient (d_33_ ≈ 8.2 pC N^–1^).

Tao et al. presented a heterostructure assembled through alternating water layers-aromatic peptide (l-tryptophan-d-tryptophan) layers.^241^ The extensive and oriented hydrogen bonding formed within the water layers resulted in notable electron transitions and dipole–dipole interactions, thereby inducing a broad-range fluorescence emission and high piezoelectric response (d_33_ of approximately 47.4 pm V^–1^) in the heterostructure peptide assemblies. By incorporating additional neutron doping techniques, the mobility of hydrogen-bonded water molecules could be facilitated, enhancing charge hopping and significantly improving the piezoelectric performance with a d_33_ of about 61.9 pm V^–1^.

Hydrogen bonding heterostructures can guide crystal orientation, enhance dipole alignment, and improve piezoelectric output in biomaterials. They show great potential, but their stability under physiological conditions and scalability for large-scale fabrication still require further investigation.

### 6.5 Chemical Cross-linking

By modifying the collagen bonding via chemical cross-linking to create stable structures, the active agent in the cross-linker could alter the chemical groups, thereby contributing to the distribution of charges along the collagen peptide backbone. Nair et al. studied the impact of three distinct cross-linkers, namely 1-ethyl-3-(3-(dimethylamino)­propyl)­carbodiimide (EDC)-*N*-hydroxysuccinimide (NHS), genipin, and tissue transglutaminase (TG2), with different reaction chemistries, on the piezoelectric behavior of collagen (Figure 6E).^235^ Collagen films cross-linked with EDC-NHS exhibited local alignment of collagen fibrils, forming thicker fiber bundles measuring 300 nm. This alignment led to an enhanced and localized vertical piezoelectricity. On the other hand, TG2- and genipin-cross-linked films showed a nonlocalized yet enhanced piezoelectricity. It was suggested that the localization of piezoelectricity in EDC-NHS-cross-linked collagen films, along with the overall improvement of piezoelectricity in TG2- and genipin-treated films, can be attributed to the alterations in the charge-carrying amino acid groups. This finding demonstrated the potential to utilize chemical cross-linking to tailor the piezoelectric behavior of collagen.

Beyond biopolymers, Gao et al. recently demonstrated that precise slight cross-linking of poly­(vinylidene fluoride-*co*-trifluoroethylene) (P­(VDF-TrFE)) with soft-chain diamines enables the formation of intrinsically elastic ferroelectrics that maintain stable ferroelectric and piezoelectric responses under strains up to 70%, effectively resolving the long-standing trade-off between crystallinity and elasticity.^242^ Overall, chemical cross-linking provides a versatile route to enhance piezoelectric output, modify mechanical performance, and stabilize functionality in both natural and synthetic systems.

In summary, while molecular engineering plays a critical role in tuning the intrinsic piezoelectric properties of biomaterials, translating these materials into practical devices requires effective strategies for their patterning, alignment, and integration. One of the major hurdles has been the reliable microfabrication and scalable assembly of piezoelectric biomaterials without compromising their functionality. In recent years, significant progress has been made in overcoming these challenges through various physical and field-driven approaches. These fabrication methods can be broadly classified into mechanical force-driven, electric-field-driven, magnetic-field-driven, and thermally driven strategies. Table 1 compares representative strategies in terms of manufacturing speed, dimensions of the final product, and characteristics. In the following four sections, we will provide a detailed discussion of these methods.

**Table 1:** Comparison of Different Microfabrication and Assembly Strategies

Driving Force	Types	Efficiency	Alignment Direction	Outcome Dimensions	Advantages	Disadvantages
Mechanical Force	Mechanical exfoliation	Low	IP or OOP, depending on material type and exfoliation direction	2-D film	(1) Unique properties of ultrathin or monolayer films	(1) Challenging to scale
					(2) Simple operation	
	Mechanical annealing	Medium	IP alignment	2-D film	(1) Enhanced piezoelectricity and crystallinity	(1) Weak alignment
					(2) Simple operation	(2) Antiparallel polarization
	3D printing/inkjet printing	Low	Hard to control	3-D custom shape	(1) Complex 3D structures	(1) Weak alignment
						(2) Poor scalability
						(3) Complicated instrumental setup
	Dip-coating	Low	IP alignment	2-D film	(1) Large scale	(1) Specialized setup needed
					(2) Simple operation	(2) Antiparallel polarization
	Spin coating	High	IP or OOP alignment along the direction of shear flow	2-D film	(1) Uniform film	(1) Weak alignment
					(2) Large scale	
	Template confine-ment	Low	IP or OOP alignment based on template structure	3-D	(1) Custom patterns	(1) Limited scalability
					(2) Nanoconfinement	(2) Specialized templates and materials needed
						(3) Antiparallel polarization
Electric Field	Electro-spinning	Low	IP or OOP along the electrical field direction	2-D film	(1) Nanofiber porous membrane	(1) Complicated instrumental setup
				3-D scaffold	(2) Unidirectional polarization alignment	
	Electro-spray	High	OOP alignment along electric field direction	2-D film, nanoparticle	(1) Rapid fabrication	(1) Complicated instrumental setup
					(2) High scalability	
					(3) Unidirectional polarization alignment	
					(4) High density	
	Parallel-plate electric field	Low	OOP alignment along electric field direction	2-D film, 3-D hydrogel	(1) Unidirectional polarization alignment	(1) Specialized setup
						(2) Challenging to scale
	IP electric field	Low	IP alignment along electric field direction	2-D film	(1) Unidirectional polarization alignment	(1) Specialized setup
						(2) Challenging to scale
Magnetic Field	/	Low	IP or OOP alignment along the magnetic field	2-D film	(1) Wireless and uniform alignment	(1) Applicable for specialized materials
						(2) Extremely high magnetic field required
						(3) Antiparallel polarization
Thermal	Thermal vapor deposition	Low	IP or OOP alignment depending on crystalline growth morphologies	2-D film	(1) Solvent-free	(1) High vacuum and temperatures needed
					(2) Precise thickness control	(2) Complicated instrumental setup
						(3) Antiparallel polarization
	Freeze casting	Medium	IP or OOP alignment guided by ice-templated growth	3-D structure	(1) Porous structures	(1) Weak alignment
					(2) Simple operation	(2) Antiparallel polarization

## 7 Mechanical Force-Driven Manufacturing Techniques

Mechanical force not only induces electricity through the piezoelectric effect but also serves as a powerful tool for the microfabrication and assembly of piezoelectric biomaterials. In recent years, diverse processing approaches based on mechanical force—such as stretching, extrusion, peeling, and shearing—have been developed to manipulate molecular orientation, create tailored structures, and enhance piezoelectric performance.

### 7.1 Mechanical Exfoliation

It is challenging for natural bulk biological tissues, like bones and wood, to display decent piezoelectricity at the macroscopic scale due to the presence of complex non-piezoelectric components and the disordered distribution of domains within these materials.^14–17^ Moreover, these biological materials typically lack ferroelectricity, preventing them from aligning piezoelectric domains and enhancing the macroscopic piezoelectricity through poling via external electric fields. In such cases, thinning the bulk materials may prove to be an effective approach. This line of thinking has been successfully applied in various studies. For instance, graphene down to a single atomic layer was found to have electrical, thermal, and mechanical properties that changed dramatically.^243,244^ Similar findings include the superelasticity of nanoscale diamond^245^ and the emergence of piezoelectricity or ferroelectricity in monolayer molybdenum disulfide (MoS_2_)^246^ and ultrathin oxides.^247^

Drawing inspiration from the processing methods of 2D materials like graphene and transition metal dichalcogenides,^248–251^ Zhang et al. developed a van der Waals exfoliation (vdWE) method to produce ultrathin SIS films by leveraging the weak van der Waals interactions between collagen fiber layers in small intestinal submucosa (SIS) tissues (Figure 7A).^152^ Using adhesive tape, dried SIS could be easily peeled into single- or few-layer collagen fibril films as thin as 100 nm (Figure 7A-b). With the vdWE approach, the piezoelectricity of the ultrathin films (d_eff_ ≈ 3.3 pm V^–1^) was enhanced by over 20-fold in comparison to the nonexfoliated raw SIS films.^152^ Bulk SIS tissues struggle to show piezoelectricity due to their IP polarization orientation and layered antiparallel domain arrangement. The presented vdWE approach successfully addressed piezoelectricity cancellation by producing ultrathin SIS films. Since van der Waals interactions are common in soft tissues, especially in collagen-rich extracellular matrices, this approach was also applied to other biological tissues, such as bovine tendon and fish swim bladder.

**Figure 7:**
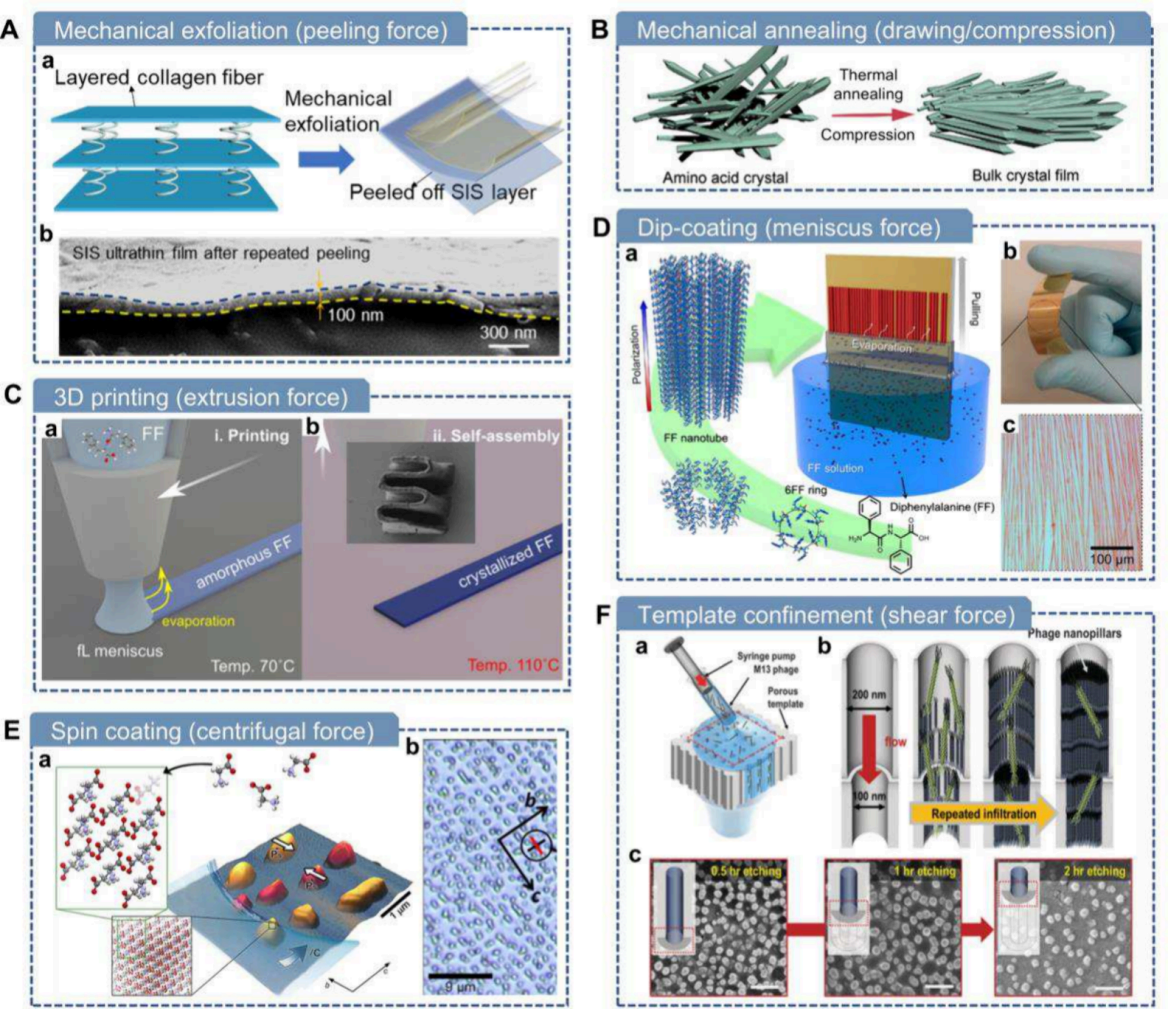
**Mechanical force-driven methods for fabricating piezoelectric biomaterials.** A) Mechanical exfoliation of soft tissues using peeling force. (a) Schematic of van der Waals layered collagen fiber network in SIS soft tissue mechanically exfoliated by adhesive tape. (b) Cross-sectional SEM image of SIS ultrathin film after repeated peeling. Reproduced with permission.^152^ Copyright 2022, Wiley-VCH. B) Schematic of mechanical annealing for fabricating amino acid crystal films by simultaneously applying mechanical compression and thermal annealing treatment. Reproduced with permission.^252^ Copyright 2023, Wiley-VCH. C) 3D printing of FF peptides. (a) Schematic of the 3D printing process and (b) the crystallized FF 3D structure. Reproduced with permission.^253^ Copyright 2021, American Chemical Society. D) Dip coating of horizontally aligned FF nanotubes. (a) Schematic of the meniscus force-driven dip-coating process. (b) Photograph of the FF nanotubes on a flexible substrate. (c) SEM image of the aligned FF nanotubes. Reproduced with permission.^254^ Copyright 2018, American Chemical Society. E) Spin coating of β-glycine crystals. (a) Schematic of the centrifugal force-driven spin coating process. (b) Optical image of the isolated crystal islands of β-glycine. Reproduced with permission.^255^ Copyright 2017, American Chemical Society. F) Template confinement-assisted fabrication of phage nanopillars. (a) Schematic of the fabrication setup based on a syringe and an AAO template. (b) Schematic of the phage nanopillar formation by repeated infiltration. (c) SEM images of the phage nanopillars in the AAO template at variable etching time. Reproduced with permission.^133^ Copyright 2015, Royal Society of Chemistry.

Kholkin et al. employed the coassembly of l,l- and d,d-enantiomers of FF monomers to fabricate layered piezoelectric biocrystals.^236^ The FF enantiomers are organized into bilayers, wherein monomers with alternating chirality are densely packed, forming a ribbon-like monoclinic structure. The synthesized layered bulk crystals are held together by weak aromatic interactions between the bilayers. This allows for simple mechanical or chemical exfoliation, resulting in two-dimensional piezoelectric biomaterials with high elasticity, thermal stability, and chemical stability. Measurement results demonstrated that each bilayer (approximately 1.5 nm thick) exhibited robust vertical piezoelectricity, with an effective d_33_ value of approximately 20 pm V^–1^.^236^

Overall, mechanical exfoliation provides a simple yet powerful strategy to unlock the hidden piezoelectricity in bulk biological tissues and supramolecular crystals by thinning them into ultrathin films. In collagen-rich SIS tissues, van der Waals exfoliation overcomes antiparallel domain cancellation, yielding significantly enhanced piezoelectric output, while layered FF crystals can be exfoliated into stable 2D piezoelectric sheets with robust performance. These examples highlight the potential of exfoliation techniques to fabricate flexible, high-performance piezoelectric biomaterials, though challenges remain in achieving uniformity, scalability, and integration into practical devices.

### 7.2 Mechanical Annealing

Mechanical annealing, by simultaneously applying mechanical drawing or compression and thermal annealing treatment, is widely used in the manufacturing of various piezoelectric polymers or crystals, primarily to enhance the crystallinity and orientational polarity (Figure 7B).^256–260^ For example, Nguyen et al. prepared PPLA films through hot pressing and then subjected the films to mechanical stretching at an annealing temperature of 90 °C.^191^ They found that as the stretching rate increased, more crystalline domains aligned along the [200] and [110] directions, while the (111) crystal face disappeared when the stretching rate reached 3.5.^261,262^ This transition signifies a shift from the α-form crystal structure with a left-handed 10_3_ helical conformation to the β-form crystal structure with a 3_1_ helical conformation.^263^ Increasing the stretching rate to approximately five greatly increased the crystallinity percentage in PLLA films and maximized the alignment of crystal domains, thereby providing the enhanced piezoelectric coefficient d_14_ of 11 pm V^–1^.^264^

Similarly, Yucel et al. utilized mechanical stretching to fabricate piezoelectric silk and studied the structural basis of silk piezoelectricity.^117^ They observed a positive relationship between the stretching ratio λ and the proportion of the II β phase component. The II β phase was identified as the crucial component in silk that is responsible for exhibiting crystalline and piezoelectric properties. They revealed that at the maximum drawn ratio of 2.7 before the material broke, the silk film exhibited enhanced crystallinity and demonstrated the best shear piezoelectricity d_14_ with a value of approximately 1.5 pm V^–1^.

Cheng et al. utilized mechanical annealing methods to fabricate dense amino acid crystal films, where the crystals exhibited a preferred orientation under mechanical stress, resulting in well-ordered polycrystalline films (Figure 7B). When subjected to compression, crystals positioned nonhorizontally experience higher stress and preferentially fracture, followed by regrowth that tends to occur in a horizontal orientation to alleviate stress concentration. Moreover, the application of high pressure facilitated the formation of larger crystals. The presence of residual water on the crystal surface can accelerate the dissolution-recrystallization kinetics, thus promoting the mechanical annealing process. Notably, the piezoelectric coefficient of mechanically annealed isoleucine crystals (d_33_ of about 1.2 pm V^–1^) was found to be 12 times higher compared to that of its crystal powders (d_33_ of around 0.1 pm V^–1^).^252^

In summary, mechanical annealing, by combining thermal treatment with drawing or compression, enhances crystallinity, phase transitions, and domain alignment in piezoelectric biomaterials. It has been shown to improve the piezoelectric output in polymers (e.g., PLLA), proteins (e.g., silk), and amino acid crystals, demonstrating versatility for high-performance structures. However, its broader use is limited by the brittleness of some biomaterials, structural integrity concerns, and scalability challenges.

### 7.3 Three-Dimensional (3D) Printing/Inkjet Printing (Extrusion Force)

3D printing techniques enable precise control over extrusion forces and directions, facilitating the microfabrication of piezoelectric biomaterials. 3D printing, also known as additive manufacturing, offers numerous advantages, including customized design capabilities, reduced processing times, minimal waste generation, and scalability.^265–268^

3D printing techniques are particularly advantageous for the fast and accurate fabrication of complex 3D piezoelectric scaffolds using raw polymeric materials. For instance, Karanth et al. utilized fused deposition modeling (FDM) technology to create PLLA scaffolds.^269^ Despite a reduction in crystallinity from 27.5% to 13.9% during the 3D printing process, the piezoelectric properties of the PLLA scaffold were retained. These scaffolds produced an output voltage of 25 mV under repeated loading, promoting bone regeneration.

Moreover, there have been advancements in ink-based 3D printing methods that involve the incorporation of piezoelectric biomaterials as constituents of the ink. For example, Kholkin et al. achieved controlled deposition of FF crystals using high-resolution and reproducible drop-on-demand printing technology.^270^ This method allowed the precise patterning of FF-based ribbonlike microcrystals onto various surfaces. Yang et al. introduced a 3D printing method for FF, involving meniscus-guided printing of amorphous FF followed by crystallization through heat treatment (Figure 7C).^253^ Ink-based 3D printing technologies have also been employed in the fabrication of piezoelectric amino acids,^271^ silk,^272^ CNC,^273^ and PHB.^274^

While 3D printing offers superior freedom of structural design, it often comes with a compromise in piezoelectric properties. This is primarily due to the unavoidable decrease in crystallinity and challenges in achieving proper orientation during the 3D printing manufacturing process. A possible solution to improve piezoelectricity is mixing piezoelectric biopolymers with inorganic piezoelectric nanoparticles such as BTO.^275,276^ However, these composites lost their inherent biodegradability.

In short, 3D and inkjet printing enable rapid, customizable fabrication of complex piezoelectric biomaterial scaffolds with high design flexibility and scalability. These techniques have been successfully applied to polymers, peptides, and other biomolecules, though often at the cost of reduced crystallinity and limited molecular orientation, leading to a compromised piezoelectric output. Incorporating inorganic nanoparticles can recover performance but sacrifice biodegradability, highlighting the need for strategies that balance structural freedom, functional enhancement, and material sustainability.

### 7.4 Dip-Coating (Meniscus Force)

For biomaterial solutions with low viscosity, shearing forces, such as streaming and centrifugal forces, are often more suitable for their microfabrication. Hence, the dip-coating approach, which relies on meniscus force, can be employed to fabricate self-assemblies of piezoelectric biomaterials (Figure 7D). For example, Lee et al. reported a meniscus-driven approach to assembling phages into large-scale films by vertically pulling substrates from phage suspensions at a low speed.^277^ During the substrate-pulling process, evaporation occurred primarily in the vicinity of the air–liquid–solid contact line, leading to the local accumulation and deposition of phage particles at the meniscus region. They then reported the first virus-based piezoelectric nanogenerator.^21^ These phage films exhibited piezoelectric strengths of up to 7.8 pm V^–1^, and the piezoelectric phage nanogenerator produced output currents of up to 6 nA and output voltages of up to 400 mV.

Furthermore, Lee et al. fabricated IP aligned FF nanotube films using the same method (Figure 7D).^254^ The morphologies and alignment of FF nanotubes were precisely manipulated by tuning the fabrication parameters, including solvent composition, peptide concentrations, and pulling speeds. Additionally, Wang et al. recently utilized the dip-coating method to fabricate a truss-like pattern of interconnected dl-alanine microfibers self-assembled on a hydrophilic substrate.^278^ The resulting piezoelectric dl-alanine biocrystal films exhibited tissue-compatible omnidirectional stretchability without compromising their piezoelectric strength.

Overall, dip-coating harnesses meniscus forces to assemble low-viscosity biomaterial solutions into large-area piezoelectric films and patterns. This method has enabled virus-based nanogenerators and aligned peptide nanotube films and stretchable amino acid microfibers, demonstrating simplicity and versatility. However, its broader application is limited by sensitivity to processing parameters, difficulty in achieving precise molecular orientation, and challenges in scaling up uniform large-area fabrication.

### 7.5 Spin Coating (Centrifugal Force)

Centrifugal forces are also utilized for fabricating biomolecular assemblies (Figure 7E).^279^ For instance, by subjecting an FF nanotube suspension containing surfactant to a centrifugal force using a spinning polytetrafluoroethylene (PTFE) rod, FF nanotubes could be assembled into well-aligned arrays along the direction of shear flow.^280^ The degree of alignment is directly influenced by the amount of surfactants added, with a maximum alignment of 80% of FF nanotubes within ±10° against the shear direction.^280^ Additionally, Kohlkin employed spin-coating of glycine solutions to fabricate ferroelectric nanostructured β-glycine films (Figure 7E).^255,281^ This technique enables the formation of quasi-regular arrays of nano- and microislands with preferentially orientated polarization axes. Alikin and Romanyuk utilized the spin-coating of organic solutions containing FF monomers to fabricate an amorphous FF layer.^282,283^ Subsequently, the amorphous layer was subjected to a regulated humidity environment, which initiated the formation of a well-aligned piezoelectric FF crystalline film. This crystallization process occurs without altering the structure and yields uniform and compact films of precise thickness. A recent study used spin-coating of a PVA–CNCs solution onto a polydimethylsiloxane (PDMS) soft mold to create a nanopatterned CNC–PVA array.^284^ The cellulose nanocrystals embedded in the polymer underwent viscous flow shaping, forming distinct surface patterns with precise spatial control. This process enabled multilayer stacking and improved both the piezoelectric response and power output density.

In summary, spin coating utilizes centrifugal force to assemble biomolecules into ordered films and arrays, enabling precise thickness control and large-area fabrication. It has been successfully applied to align FF nanotubes, form ferroelectric β-glycine films, and create patterned CNC–polymer composites with enhanced piezoelectric output. Despite its versatility and scalability, challenges remain in achieving uniform crystallinity and controlled orientation and maintaining performance consistency across large substrates.

### 7.6 Template Confinement

Template confinement techniques, such as screen printing and soft lithography, utilize templates with precise topological structures to capture biomolecules in specific areas, resulting in molded morphologies. These methods are broadly applied in microelectromechanical systems for creating customized micropatterns.^21,285,286^ Typically, the templates are composed of highly flexible polymeric resins, such as PDMS, which enables easy removal of the microfabricated peptide patterns after molding. For instance, Lee et al. employed a micropatterned PDMS mold as a template to control interfacial forces and fabricate vertically aligned phage nanostructures. These vertically ordered structures exhibited strong unidirectional polarization and had piezoelectric coefficient values three times higher than those of IP aligned structures.^134^

Furthermore, many studies have focused on the use of nanoporous templates such as anodic aluminum oxide (AAO) for fabricating piezoelectric biomaterials.^287,288^ For instance, Shin et al. created nanopillar virus arrays exhibiting increased piezoelectricity by extruding negatively charged M13 bacteriophages into a positively charged AAO porous template (Figure 7F).^133^ Additionally, the growth and piezoelectric behavior of β-glycine and l-alanine within nanoporous matrices were also investigated.^289–291^ These studies revealed that while β-glycine crystals are metastable under ambient conditions, they become the stable phase when confined to nanopores.

In short, template confinement enables precise control of the biomaterial assembly by guiding molecules into predefined micro- or nanoscale patterns, producing ordered structures with enhanced piezoelectric performance. This approach provides excellent structural precision and versatility, but its broader application is limited by challenges in achieving uniform polarization alignment, as well as difficulties in fabricating high-density or large-scale structures.

Overall, mechanically driven manufacturing offers notable advantages in production speed, scalability, ease of operation, and design flexibility. Techniques such as 3D printing enable the fabrication of complex three-dimensional architectures, broadening the structural possibilities for piezoelectric biomaterials. Nevertheless, these methods still face challenges in achieving precise polarization alignment and maintaining a strong piezoelectric performance, both of which are critical for practical applications. To overcome these limitations, many studies have combined mechanical processing with additional stimuli, such as electric, magnetic, or thermal fields, to further enhance piezoelectric output.

## 8 Electric Field-Driven Manufacturing Techniques

Electric fields are extensively employed for the large-scale arrangement of substances due to their ability to provide homogeneous forces. For piezoelectric biomaterials, which typically possess charges and intrinsic polarity, electric fields can drive the controlled microfabrication and self-assembly processes and, importantly, facilitate polarization alignment along electric field directions. Depending on the configuration, electric field-driven manufacturing methods can be broadly classified into three categories: tip-induced, parallel-plate, and in-plane (IP) electric field approaches.

### 8.1 Tip-Induced Electric Field

A strong electrostatic field could disrupt the stability of the liquid–air interface of a microfluidic droplet, resulting in the formation of a conical-shaped interface known as the Taylor cone, which occurs when the microfluid becomes charged beyond the Rayleigh limit. When the sample is subjected to a sufficiently strong electric field, either a stream of droplets or a thin liquid jet that subsequently breaks up into numerous droplets is ejected from the tip of the cone. These phenomena have inspired various microfabrication techniques such as electrospinning, electrospray, and droplet focus deposition.^292^

#### 8.1.1 Electrospinning

Electrospinning is specifically used to generate fine fibers or nanofibers from a polymer solution (Figure 8A). It has been utilized in various fields, including scaffolds, filtration membranes, and textiles. The resulting fiber membranes possess excellent elasticity and can withstand high strains. Moreover, the high electric field applied during the spinning process facilitates the polarization alignment, resulting in nanofibers with enhanced piezoelectric properties. Therefore, various piezoelectric biopolymers such as PLLA,^192,293–295^ PHB,^201,203^ PBLG,^95^ chitin,^296^ and silk^297^ have been demonstrated to be fabricated into nanofibers using electrospinning. For example, Farrar et al. used electrospinning to fabricate PBLG fibers, applying directional shear and electric fields to align permanent dipoles and helical chains along the fiber axis or field direction (Figure 8A-a).^95^ This work provided the first direct evidence of poled molecular dipoles and linked them to the piezoelectric properties of electrospun PBLG.^95^ Similarly, Nguyen et al. electrospun PLLA nanofibers using different drum rotation speeds, producing membranes with varying fiber orientation.^192^ Low-speed collection led to poor alignment and low crystallinity, resulting in negligible piezoelectricity. In contrast, higher speeds enhanced both domain and fiber alignment, and by optimizing collector and jet speeds, they achieved improved piezoelectric performance.^189^

**Figure 8:**
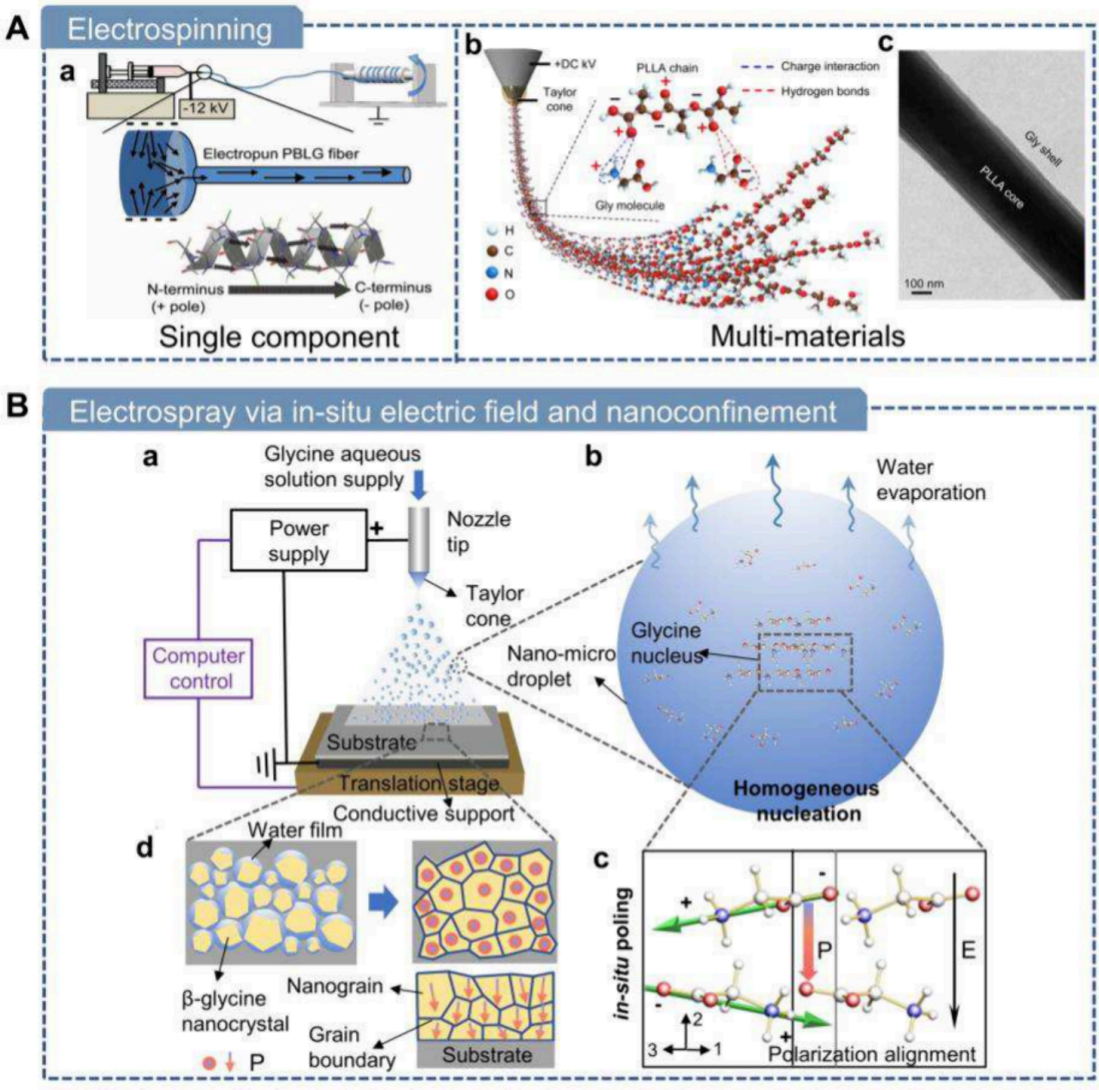
**Tip-induced electric field-driven methods for fabricating piezoelectric biomaterials.** A) Electrospinning technique. (a) Schematic of electrospun single-component PBLG fibers. Reproduced with permission.^95^ Copyright 2011, Wiley-VCH. (b) Schematic of the electrospun glycine-PLLA nanofiber. (c) TEM image showing the core–shell structure of the glycine-PLLA nanofiber. Reproduced with permission.^219^ Copyright 2024, American Association for the Advancement of Science. B) Electrospray technique via synergistic nanoconfinement and in situ electric field. (a) Schematic of electrospray deposition process for fabricating β-glycine nanocrystalline films. (b) Schematic of the homogeneous nucleation and crystallization of β-glycine in the in-flight nano-micro droplets. (c) Schematic of the polarization alignment of glycine molecules along the in situ electric field direction during the nucleation process. (d) Schematic of the forming process of β-glycine films with compact and well-aligned nanograins. Reproduced with permission.^53^ Copyright 2023, Springer Nature.

Electrospinning requires a solution with sufficient viscosity and surface tension to facilitate fiber formation and stretching. Although small biological molecules such as amino acids and dipeptides exhibit higher piezoelectricity, the low viscosity and low surface tension of their solution make direct electrospinning challenging. One approach to overcome this challenge is by simply mixing piezoelectric small biomolecules with polymer solutions or by employing coaxial electrospinning to create core–shell structured piezoelectric fibers.^298–300^ These fibers possess a polymer shell (e.g., PVA) that provides the electrospinning capability and flexibility, while the nanocrystals formed by the biological molecules (e.g., β-glycine) in the core contribute to the piezoelectric properties.^301^ Small biomolecules in the core may also promote the favorable orientation and stabilization of the piezoelectric β-phase structure in the polymer (PLLA) shell through strong intermolecular interactions (Figure 8A-b).^219^

In short, electrospinning enables the fabrication of piezoelectric nanofibers with a controlled orientation, combining mechanical flexibility with enhanced polarization alignment. It has been successfully applied to a range of biopolymers, such as PLLA, PHB, PBLG, chitin, and silk, where optimized processing improves crystallinity and dipole IP alignment.

#### 8.1.2 Electrospray

Although electrospinning can achieve highly aligned fibers with directional polarization, the polarization direction is typically parallel to the fiber axis and within the plane of the fiber membrane. However, for many applications, obtaining an OOP piezoelectric response with polarization aligned perpendicular to the film surface is considered ideal. Electrospray is primarily used to produce charged droplets or aerosols from a liquid solution. It is commonly employed in applications such as mass spectrometry, drug delivery, and coating deposition. While there have been studies exploring the fabrication of piezoelectric inorganic ceramic films and synthetic organic polymers such as PZT and PVDF using electrospray,^292,302–306^ there is limited research on manufacturing piezoelectric biomaterials through this technique.

Recently, Zhang et al. reported a generalizable approach for fabricating large-scale, high-performance, and customizable piezoelectric biocrystal thin films using electrospray deposition, revealing an active self-assembly mechanism driven by nanoconfinement and in situ poling (Figure 8B).^53,307^ The synergistic effect of electric fields and confinement induced glycine nucleation and alignment, resembling sintering and poling in piezoceramics. This led to uniform OOP crystal orientation and enhanced piezoelectricity, with strain and voltage coefficients of 11.2 pm V^–1^ and 252 mV m N^–1^, respectively. Notably, nanoconfinement promoted the formation of metastable β-glycine, which is notoriously difficult to obtain under ambient conditions. The thermostability of nanocrystalline films was greatly improved without a phase transition until melting (192 °C), compared with bulk crystals (67 °C), greatly broadening the usage temperature range and piezoelectric stability. The method also allows scalable fabrication of films with tunable dimensions and structures, including freestanding particles and flexible composites,^292^ and is potentially extendable to other molecular biocrystals and organic–inorganic piezoelectrics.^71^

Building on a similar active self-assembly strategy, Li et al. developed a roll-to-roll multinozzle electrospray system for scalable fabrication of flexible, bioresorbable piezoelectric composite films using a glycine–PVP (polyvinylpyrrolidone) solution.^308,309^ The process, driven by a thermal-electric coupled field, enabled one-step high-throughput deposition at speeds up to 10^8^ μm^3^ s^–1^, which is 2 orders of magnitude faster than conventional methods. The resulting films exhibited excellent flexibility (0.3 GPa), nearly 100 times more flexible than pure glycine crystals, and maintained uniformly high piezoelectricity (10.8 pm V^–1^).

In summary, electrospray has emerged as a powerful method for fabricating large-area piezoelectric biomaterial films with uniform OOP polarization and enhanced thermal stability. It enables high-density, single-component thin films with controlled nucleation and stabilization of metastable phases such as β-glycine, resulting in an exceptional piezoelectric output. Recent advances, including scalable roll-to-roll systems, highlight its promise for flexible and bioresorbable devices. Moreover, this strategy is expected to be extendable to other biomolecular materials and even organic–inorganic ferroelectric systems. Nevertheless, extending this technique to the controlled fabrication of complex three-dimensional architectures remains a key challenge.

### 8.2 Parallel-Plate Electric Field

For electrospinning and electrospray, the tip-induced electric field is applied not only to fabricate materials on a large scale but also to control polarization. Besides, there was also much research focusing on new methods that combine conventional manufacturing methods with a parallel plate electric field, which mainly plays the role of in situ aligning polarization during fabrication. This strategy has been applied to many biomaterials, such as peptides,^96,98,310^ cellulose,^180,184,311^ silk,^312^ and gelatin.^313^ Here, we focus on introducing several representative works.

#### 8.2.1 Epitaxial Growth under Electric Field

FF dipeptides can self-assemble into peptide nanotubes with strong piezoelectric properties.^86^ However, these nanotubes typically exhibit random polarization during the growth process. Moreover, the high coercive field of around 30 MV cm^–1^ associated with these dipeptides makes it practically impossible to switch the polarization of grown peptide nanotubes via electric poling.^106^ To enable the practical utilization of piezoelectricity on a larger scale, it becomes crucial to grow FF peptide nano- and microstructures with uniform polarization. As shown in Figure 9A, Yang et al. introduced an engineered FF seed film growth method to align FF structures.^98,310^ Through precise control of water diffusion during the formation of seed films, they created seeds with most of the vertical domains exhibiting antiparallel polarizations. Subsequent application of a parallel-plate electric field ensured uniformity of the polarizations. Additionally, the engineered seed film facilitated the epitaxial growth of microrods. The researchers successfully obtained vertically aligned FF peptide microrods with controlled polarization,^310^ demonstrating significantly higher piezoelectricity compared to the array grown without an electric field. The effective vertical piezoelectric constant (d_33_) for an individual microrod was measured as 17.9 pm V^–1^.^310^

**Figure 9:**
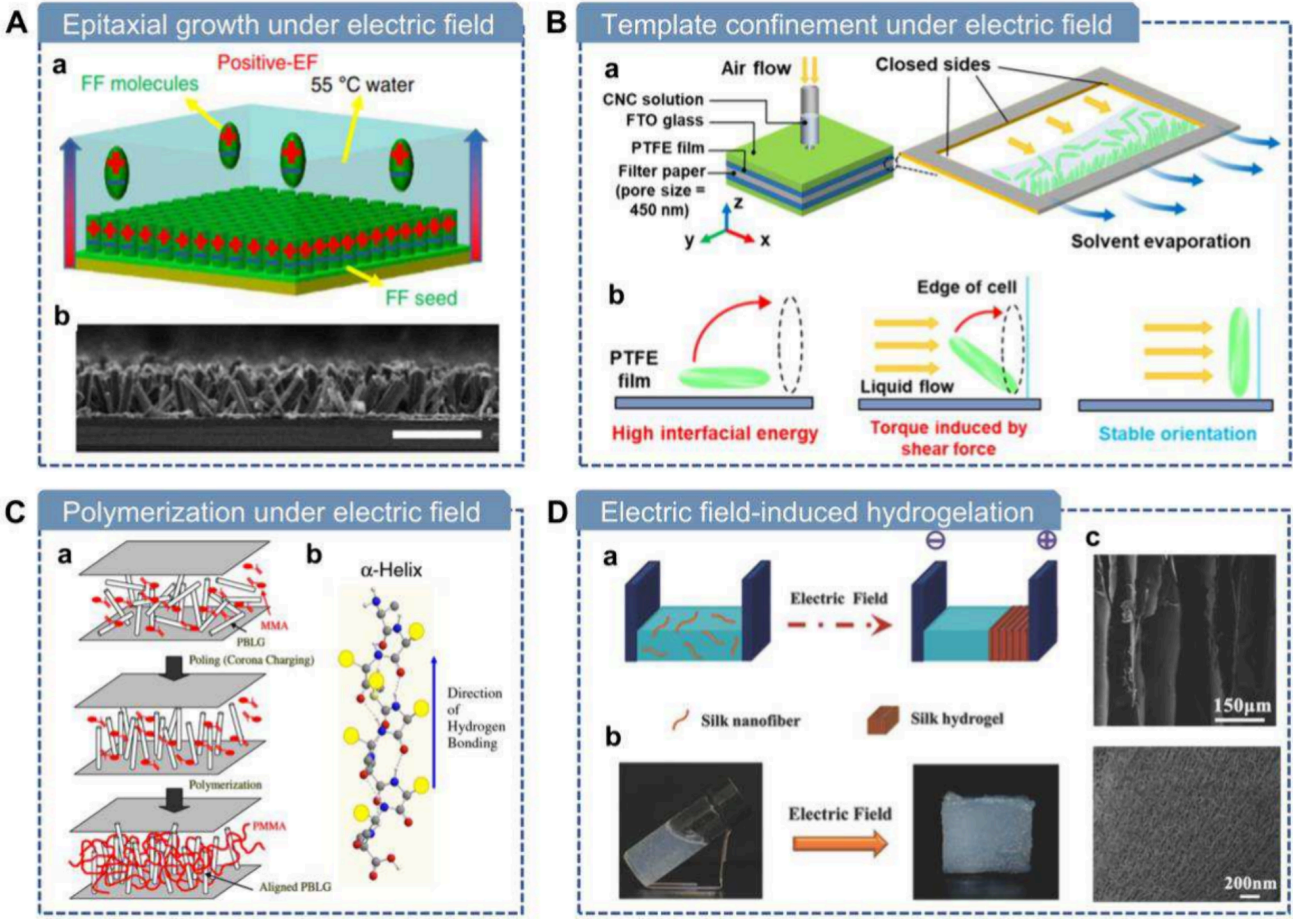
**Parallel-plate electric field-driven methods for fabricating piezoelectric biomaterials.** A) Electric field-assisted epitaxial growth of vertically aligned FF microrod arrays. (a) Schematic of the epitaxial growth process. (b) Cross-sectional SEM image of the vertically aligned FF microrods. Reproduced with permission.^310^ Copyright 2016, Springer Nature. B) Vertically aligned CNC films via template confinement and electric field poling. (a) Schematic of the fabrication setup based on a confinement cell and an electric field applied in situ. (b) Schematic of the underlying mechanism of vertically aligning CNC films. Reproduced with permission.^184^ Copyright 2020, American Chemical Society. C) Corona discharge-driven simultaneous poling and polymerization of PBLG and MMA homogeneously mixed solution. (a) Schematic of the manufacturing process of PBLG–PMMA composite films. (b) α-Helical PBLG polypeptide structure aligned by the electric field. Reproduced with permission.^96^ Copyright 2008, Elsevier. D) Silk hydrogel assembly via applying an external electric field. (a) Schematic of the silk hydrogel formation process. (b) Photographs of the silk solution transitioning to hydrogel. (c) SEM images of the frozen dried hydrogels showing the hierarchically assembled structure. Reproduced with permission.^312^ Copyright 2016, Wiley-VCH.

#### 8.2.2 Template Confinement under Electric Field

CNC assemblies typically possess a fibrous structure, leading to a sheet-like arrangement with predominant IP polarizations. Consequently, they commonly exhibit shear piezoresponses and are limited in the level of OOP polarization. Achieving a vertical alignment of CNCs, where the dipole moments align perpendicular to the film surface, would be geometrically advantageous for showing significant OOP piezoelectric response in bulk form.^184,314^ Therefore, Wang et al. successfully created vertically aligned CNC films using a parallel-plate electric field-assisted confinement cell technology (Figure 9B).^184^ This innovative technique utilized the interplay of high interfacial energy and torque generated by shear stress from the capillary flow, resulting in the stabilized vertical alignment of CNC microrods. Importantly, all dipole moments were oriented perpendicular to the CNC film surface with the consistent polarization direction tuned by the high electric field during synthesis. Notably, the average piezoelectric coefficient was verified by PFM measurements with a d_33_ value of 19.3 ± 2.9 pm V^–1^.^184^

#### 8.2.3 Polymerization under Electric Field

PBLG rods assembled into films on solid substrates tend to align parallel to the film surfaces owing to inevitable interactions with the substrates, greatly weakening their OOP piezoelectric response.^97,315–317^ To overcome this challenge, Farrar et al. utilized corona discharging to align the PBLG rods in a direction perpendicular to the PBLG-poly­(methyl methacrylate) (PMMA) composite film surface (Figure 9C).^96^ The fabrication process involved dissolving PBLG in liquid monomer MMA and subsequently polymerizing the monomer. At the same time, the corona discharging facilitated the alignment of dispersed PBLG rods along the parallel-plate electric field direction prior to solidification into a mixed PBLG–PMMA state. The resulting piezoelectric coefficient was measured to be up to 23 ± 3.5 pC N^–1^.^96^ In contrast, composite films produced without corona discharging through thermal polymerization did not exhibit evident piezoelectric responses resulting from the random thermal motion of the PBLG rods during the film formation process.

#### 8.2.4 Electric-Field-Induced Hydrogelation

Electric fields have been proven to be an effective means of synthesizing anisotropic hydrogels. There were several studies applying this strategy to the fabrication of piezoelectric biomaterial gels.^85,312,313,318^ For example, Ma et al. fabricated a piezoelectric gel by applying an electric field to gelatin dissolved in a glycerol solution, forming a cross-linked network.^313^ The piezoelectric behavior of the gel was primarily attributed to the alignment of molecular dipoles induced by the electric field during the gelation process, leading to an output voltage of around 50 mV. In 2016, Lu and colleagues utilized a parallel-plate electric field to drive the layered arrangement of nanofibers and create an anisotropic silk hydrogel (Figure 9D).^312^ The nanofiber alignment was driven by the electrostatic interaction of PH-induced electric dipoles of silk nanofibers with high β-sheet content and the applied electric field. The entanglement of nanofibers led to the formation of layers composed of aligned nanofibers, and the inherent repulsion among negatively charged entities caused the separation of these layers, resulting in a layered anisotropic structure.^312^ Additionally, they also demonstrated the generality of this electric-field-induced layered hydrogelation strategy by producing anisotropic hydrogels of peptide nanofibers.

In summary, parallel-plate electric fields provide an effective means of aligning dipoles and guiding the controlled assembly of piezoelectric biomaterials during fabrication. Representative strategies include electric-field-assisted growth of FF peptide microrods, vertical alignment of CNC films, and corona-discharge polymerization of PBLG composites, all enhancing piezoelectricity. Electric fields have also been used to induce anisotropic hydrogelation in silk, gelatin, and peptide nanofibers, producing aligned structures with measurable outputs. However, parallel-plate fields alone are often insufficient for shaping and usually need to be combined with other fabrication methods. Moreover, the requirement of high voltages introduces breakdown risks, and achieving precise control in complex 3D architectures remains a major challenge.

### 8.3 In-Plane (IP) Electric Field

For the horizontally distributed microstructures of piezoelectric biomaterials, achieving uniform IP polarization is also crucial for enhancing their piezoelectricity. Selective wettability offered a way to control the microfabrication and alignment of peptide superstructures, creating patterned architectures on targeted substrates.^319–321^ Upon application of the FF solution at the interface of a hydrophobic substrate and a hydrophilic substrate, which was created using selective UV/ozone exposure, the solution exhibited an anisotropic spreading behavior. This resulted in the fabrication of nanotube arrays, specifically on the hydrophilic region of the substrate. However, the polarization of these horizontally aligned peptide nanotubes was not uniform. Hence, Rodriguez et al. introduced an IP electric field within the hydrophilic region and achieved uniform polarization in the microfabricated film.^320^ This approach greatly increased the output performance of the flexible piezoelectric nanogenerator based on peptide nanotube composites. Under the bending conditions, the output voltage and current were measured to be 6 V and 60 nA, respectively.^320^ In addition to applying an external IP electric field, there were also several studies utilizing the electrostatic interaction between the substrate itself and the IP grown superstructure to assist in unidirectional polarization alignment.^254,278^

In short, IP electric fields are effective for achieving uniform polarization in horizontally aligned piezoelectric biomaterials. By a combination of substrate patterning or electrostatic interactions with applied fields, peptide nanotube arrays have been aligned to produce enhanced nanogenerator outputs. However, this strategy is restricted to IP polarization control, which limits its applicability to broader device architectures and multifunctional applications.

In summary, the electric-field-driven fabrication of piezoelectric biomaterials has seen extensive research and development. Unlike conventional piezoceramics exhibiting ferroelectricity, most piezoelectric biomaterials either are non-ferroelectric or possess extremely high coercive fields, making postfabrication poling impractical. Thus, applying an electric field during fabrication is particularly important to induce polarization alignment and achieve a high piezoelectricity. Moreover, by adjustment of the direction of the applied field, polarization can be tuned in either IP or OOP orientations. Nevertheless, the relatively low efficiency of methods such as electrospinning and the high cost of experimental setups still limit their widespread use. Moving forward, balancing high piezoelectric performance with scalable manufacturing efficiency and reduced cost will be critical for advancing piezoelectric biomaterials toward large-scale engineering applications.

## 9 Magnetic Field-Driven Manufacturing Techniques

In addition to electric fields, magnetic fields offer a compelling external cue for directing the fabrication and alignment of piezoelectric biomaterials. Many molecular materials exhibit diamagnetism, a weak magnetic response that arises when an applied magnetic field alters the orbital motion of electrons.^85,322,323^ This diamagnetism effect enables their alignment under strong magnetic fields. Furthermore, magnetic responsiveness can be enhanced by incorporating ferro- or paramagnetic nanoparticles into these biomolecular superstructures.

FF peptides, in particular, represent a notable example of magnetically responsive piezoelectric biomaterials. FF Peptide monomers show negligible magnetic response due to the dominant influence of Brownian motion. However, the magnetic susceptibility of a molecule increases with its size and structural organization. The stacking of aromatic rings through π–π interactions in assembled molecules further amplifies their magnetic susceptibility. As a result, their assembled superstructures, particularly 1D forms like nanotubes and fibers, can be effectively manipulated using magnetic fields. Hill et al. demonstrated that FF peptide nanotubes can be aligned in strong magnetic fields (>7 T) without the need for additional functionalization (Figure 10A).^322^ They observed increasing alignment of FF nanotubes along the field direction as magnetic field strength increased to 12 T. The alignment is attributed primarily to the diamagnetic anisotropy of the aromatic rings within the FF structure, rather than peptide bonds, and is supported by a theoretical model quantifying the magnetic torque. To lower the required magnetic field intensity, Gazit and co-workers developed a strategy to impart magnetic properties by assembling FF monomers in the presence of magnetite (Fe_3_O_4_) nanoparticles (Figure 10B).^321^ These nanoparticles adhered uniformly to the nanotube surfaces via hydrophobic interactions without disrupting the tube formation. The resulting magnetic coated FF nanotubes could be efficiently aligned under magnetic fields as low as 0.5 T. Radvar et al. presented a strategy combining coassembly and magnetic alignment to fabricate nanofibrous membranes, where aromatic cationic peptides self-assemble with hyaluronic acid (HA) into aligned structures under an external magnetic field.^324^ The peptides are engineered with a tetraphenylalanine segment to promote self-assembly through hydrophobic and π–π interactions and a lysine-rich domain to facilitate electrostatic interactions with HA. Under an external magnetic field, the high diamagnetic anisotropy of the phenylalanine residues induces alignment of the nanofibers within the membrane. This alignment guides mesenchymal stem cells to elongate along the fiber direction, demonstrating potential applications in soft tissue engineering.

**Figure 10:**
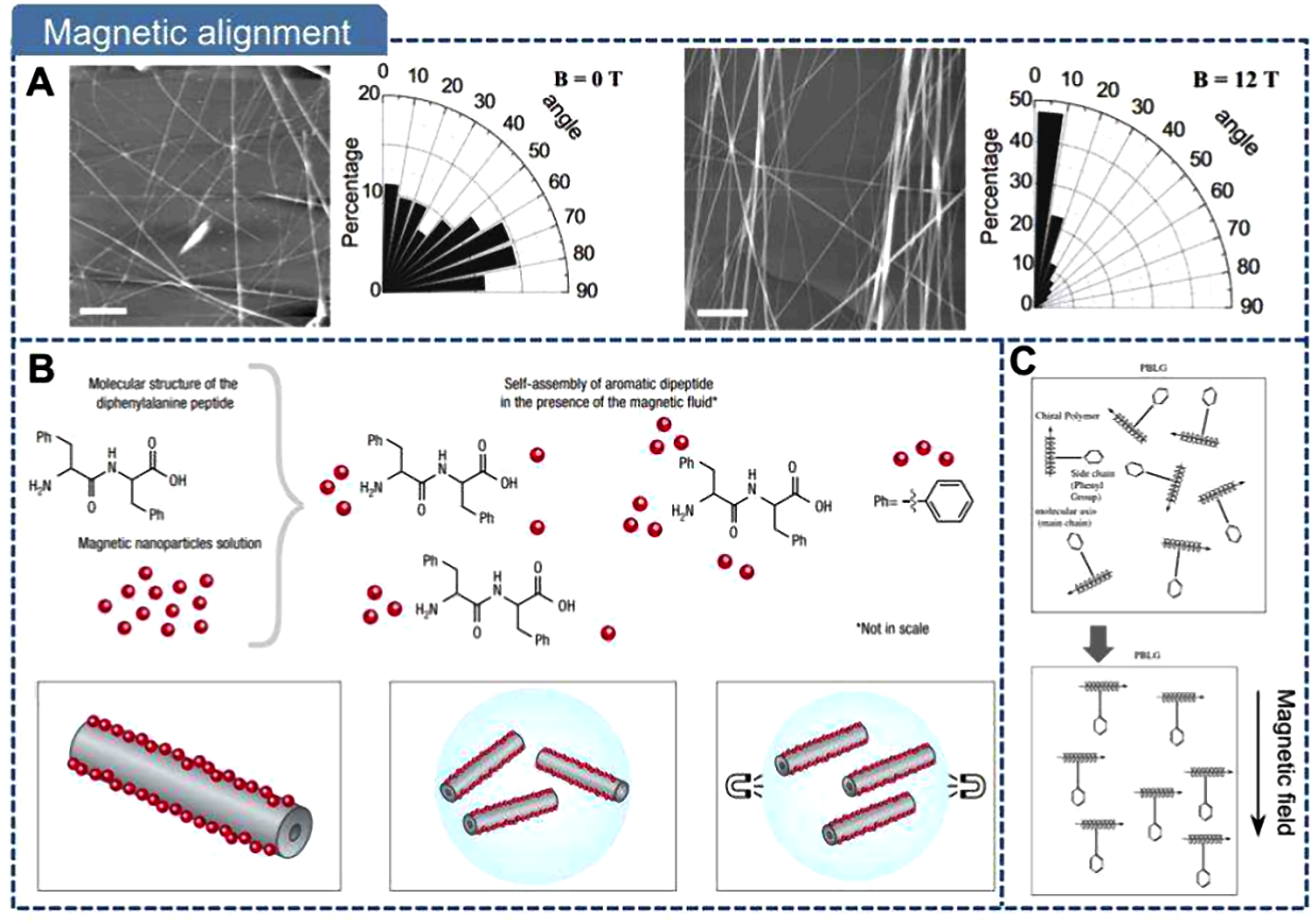
**Magnetic field-driven methods for fabricating piezoelectric biomaterials.** A) AFM topography images of FF nanotube residues obtained after evaporation of HFIP–water solution droplets under magnetic fields of 0 T (left) and 12 T (right). Reproduced with permission.^322^ Copyright 2007, Wiley-VCH. B) Schematic illustration of dipeptide monomers self-assembling in a ferrofluid with magnetite nanoparticles, showing randomly oriented magnetic tubes before and horizontally aligned tubes after magnetic field exposure. Reproduced with permission.^321^ Copyright 2006, Springer Nature. C) Schematic illustration of PBLG alignment under a magnetic field. Reproduced with permission.^97^ Copyright 2004, IOP Publishing.

Similarly, PBLG, a chiral polypeptide with rigid α-helical backbones and pendant aromatic groups, has also been shown to align effectively under strong magnetic fields due to the combined effects of its structural rigidity and diamagnetic anisotropy (Figure 10C).^97,325^ The α-helical conformation provides mechanical stiffness and a well-defined molecular axis, while the aromatic rings generate significant diamagnetic anisotropy that enables torque under magnetic fields. Crucially, magnetic alignment was only achieved when PBLG was cast from a liquid crystalline phase formed at concentrations above 30 wt% in 1,2-dichloroethane, where cooperative domain behavior facilitates orientation.^97^ X-ray diffraction confirmed that the chain alignment occurs perpendicular to the magnetic field direction. As a result, PBLG films prepared under 10 T fields exhibited a high shear piezoelectric constant (d_14_ up to 26 pC N^–1^), significantly surpassing values reported for unoriented or drawn PBLG films (<1 pC N^–1^).

A magnetic field was also used to assist the fabrication of piezoelectric composites, in which non-piezoelectric fillers served as magnetic components. By casting PLLA/hydroxyapatite (Hap) mixtures under a strong magnetic field (10 T), the Hap particles were aligned, enabling subsequent mechanical drawing without membrane breakage.^326^ The resulting films exhibited high molecular orientation and enhanced crystallinity, leading to a shear piezoelectric constant of ∼20 pC N^–1^, significantly higher than that of pure PLLA (∼9–10 pC N^–1^). This work highlights the synergistic effect of magnetic alignment and composite design in improving the piezoelectric response of chiral polymer systems.

In short, magnetic fields can noninvasively realign electric dipoles while offering advantages such as no risk of breakdown and excellent uniformity. However, compared to electric fields, the application of magnetic fields in piezoelectric materials remains relatively limited.

## 10 Thermally Driven Manufacturing Techniques

Thermal engineering techniques, including heating and freezing, were also widely used in the microfabrication of piezoelectric biomaterials, such as thermal vapor deposition, thermal annealing, isotropic freeze-drying, and directional freeze casting.

### 10.1 Thermal Vapor Deposition

In most cases, the self-assembly of piezoelectric biomaterials relies on a solvent environment. Directly assembling the biomaterial powders into organized superstructures, such as through thermal-evaporation-driven sublimation, offers a solvent-free approach. Notably, the conformation of the self-assembled superstructures, such as the thickness, morphologies, and topological structures, can be controlled by adjusting sublimation parameters, including the vacuum level, heating temperature, deposition time, etc.

Among the thermal evaporation methods, physical vapor deposition (PVD) stands out as a prominent technique for self-assembling and microfabricating piezoelectric biomaterials. For example, Gazit and co-workers reported peptide nanotube arrays obtained by vapor deposition.^327^ In their study, FF powder was heated to 220 °C under a vacuum, vaporized into the gas phase, and deposited onto a substrate, forming a film with a self-assembled nanotube array structure (Figure 11A). Notably, the resulting peptide-arraying films exhibited significant piezoelectric properties, which can be attributed to a higher level of integration and uniform alignment of the self-assembled peptide nanotubes during the PVD process.^327,328^ In addition, Yang et al. reported a modified Stranski–Krastanov (S–K) growth approach based on PVD to create well-organized amino acid array structures.^329^ They examined the growth of vertically aligned valine sheet arrays by manipulating the chamber pressure, substrate temperature, and source-substrate distance, revealing a “layer-plus-sheet” growth process. The modified S–K growth mode was also successfully utilized to fabricate nanostructures of other amino acids, including leucine and isoleucine. This growth mode played a crucial role in achieving uniform and adjustable morphologies of amino acid nanostructures, leading to a notable improvement in their piezoelectric strength. The valine arraying films exhibited a maximal effective piezoelectric constant of 11.4 pm V^–1^, approaching its highest predicted value. Furthermore, the output-circuit voltage of the piezoelectric energy harvester based on the valine array was approximately 4.6 times higher than that of the valine powder-based device.^329^

**Figure 11:**
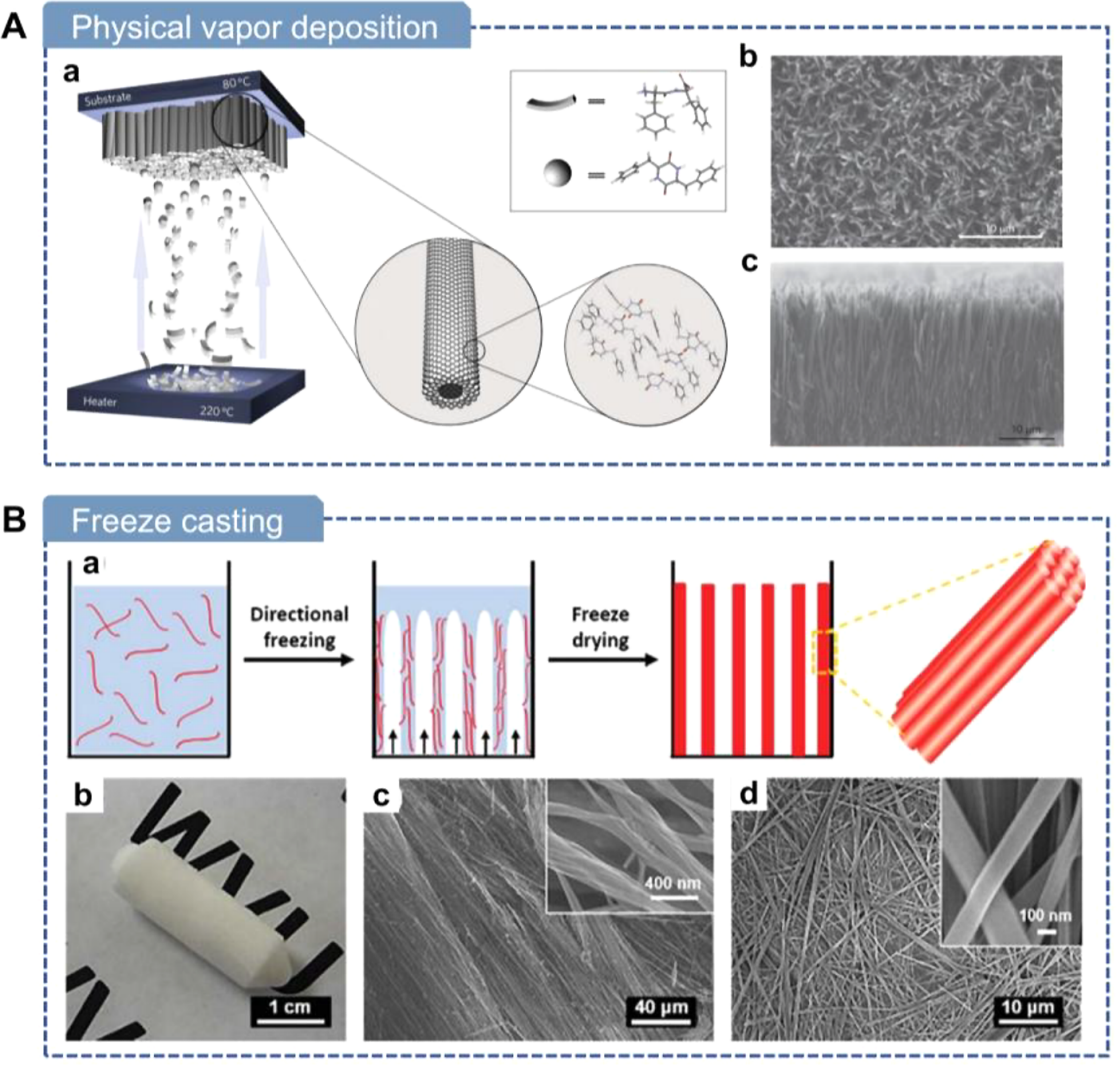
**Thermally driven methods for fabricating piezoelectric biomaterials.** A) PVD technique. (a) Schematic of the PVD process of peptide nanotube arrays. (b) Surface topography and (c) cross-sectional SEM images of peptide nanotube arrays. Reproduced with permission.^327^ Copyright 2009, Springer Nature.^330^ B) Directional freeze casting technique. (a) Schematic of the formation process of aligned nanofibers using directional freeze-drying. (b) Photograph of a freeze-dried 3D Phe nanofibrous monolith. (c,d) SEM images of Phe nanofibers fabricated by (c) directional freeze-drying and (d) conventional drop-casting. Reproduced with permission.^330^ Copyright 2015, Royal Society of Chemistry.

### 10.2 Freeze Casting

Freeze casting, also known as ice templating, is a highly versatile approach widely used for producing porous materials based on polymers, ceramics, metals, and biomolecules.^331,332^ While numerous studies have employed freeze-drying to fabricate biopolymer scaffolds for tissue engineering applications,^333,334^ their piezoelectric properties have not been effectively validated. This is primarily because freeze-dried scaffolds tended to have an amorphous rather than highly crystalline structure and their isotropic structures lacked well-defined alignment. Still, the application of directional freeze casting for the ordered self-assembly of piezoelectric small biomolecules holds significant promise. Hence, Li et al. utilized directional freeze casting to direct the self-assembly of l-phenylalanine (Phe) and FF nanofibers into well-aligned 3D nanofibrous superstructures and to produce Phe-PVA nanofibrous composites (Figure 11B).^330^ During the freezing process of the Phe solution in liquid nitrogen, anisotropic ice crystals exhibited preferential growth along the temperature gradient, forming parallel ice fronts. Simultaneously, the Phe nanofibers from the solidifying solution were concentrated and aligned by growing freeze fronts (Figure 11B-a). Consequently, Phe nanofiber bundles formed among the freeze fronts, ultimately constructing an interconnected 3D micronetwork with compartmental structures (Figure 11B-c). The nanofiber density and alignment could be controlled by adjusting processing parameters, including the concentration and pH of the solution, as well as the freezing temperature.^330^

Overall, thermal engineering methods enable the fabrication of ordered piezoelectric biomaterials. PVD allows solvent-free growth of peptide and amino acid arrays with enhanced alignment and piezoelectricity, while directional freeze casting guides amino acid nanofibers into 3D networks. These techniques show strong potential, but challenges remain in controlling the polarization orientation, achieving complex architectures, and ensuring crystallinity and uniformity at large scales.

## 11 Bioelectronics and Biomedical Applications

Bioelectronics or medical devices based on piezoelectric materials can serve as biosensors, actuators, or electrical stimulators, enabling the monitoring of biophysiological signals and the treatment of diseases. These devices often come into direct contact with external or internal tissues and remain in biological systems after operation, raising concerns about immunotoxicity and electronic waste. Fortunately, piezoelectric biomaterials, as a gift given by Mother Nature, hold promise to play a significant role in the future design of bioelectronics and medical devices. These biomaterials exhibit unique biodegradability and resorbability, allowing them to safely dissolve within the body after the completion of their tasks. Although the application of piezoelectric biomaterials is still in its infancy compared to that of well-established traditional ceramics or synthetic polymer counterparts, their exciting potential continues to drive rapid development in this emerging field in recent years. In this section, we present an overview of the cutting-edge representative biomedical applications of piezoelectric biomaterials in sensing, actuation, energy harvesting, filtration, and tissue engineering.

### 11.1 Sensors

Sensors can be primarily categorized into three types: capacitive, piezoresistive, and piezoelectric sensors. Among them, the first two types of sensors require an external power supply to function properly, limiting their application in biomedical fields due to their large size, poor flexibility, limited biocompatibility, and short battery life. Nevertheless, sensors based on piezoelectric biomaterials have garnered research interest due to their natural flexibility, biocompatibility, low detection limits, high sensitivity, and accuracy.^335^ Importantly, these biomaterials, due to their inherent piezoelectric properties, can generate sensing electrical signals through mechanical actions of the body without the need for batteries. Another promising property is biodegradability, which makes them suitable for developing future transient and ingestible biosensors for short-period medical applications, avoiding additional removal surgery.

Measurement of physiological pressures within the body, such as the brain, lungs, and eyes, using flexible and biocompatible pressure sensors, is essential for monitoring health conditions and preventing the accumulation of harmful internal stress in impaired organs. Shan et al. reported a biodegradable PLLA/BTO piezoelectric sensor (PBPS) composed of PLLA fibers doped with BTO nanoparticles for real-time assessment of motor function recovery following nerve injury (Figure 12A).^336^ The PBPS was implanted alongside tissue scaffolds used for the treatment of nerve injuries in rat models. The output signal of PBPS increased as the repair process progressed, exhibiting good consistency with conventional electromyographic (EMG) testing signals. Moreover, the integration of PBPS with a wireless module enabled wireless evaluation of motor function, effectively circumventing the temporal and spatial limitations associated with traditional evaluation methods.^336^ Curry and Nguyen presented a physiological force sensor based on an annealed stretched and 45° cut piezoelectric PLLA film.^191^ The sensor had a broad pressure measurement range (0–18 kPa) suitable for various significant physiological pressures, including intracranial pressure (below 2.7 kPa) and intraocular pressure (below 5.3 kPa). The researchers validated the medical application of the sensor by implanting it into mice to monitor diaphragm contraction pressure.^191^ During accelerated degradation at 74 °C, the sensor completely degraded within 56 days.

**Figure 12:**
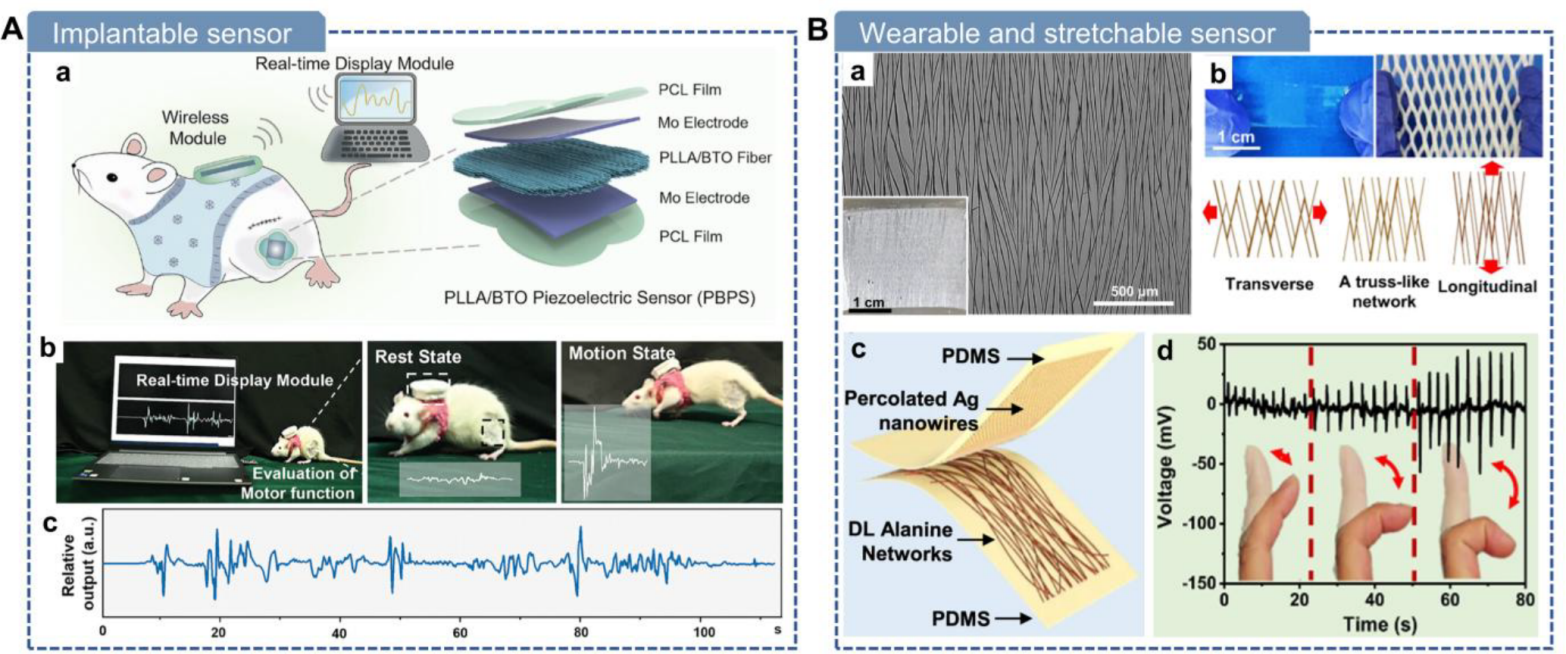
**Sensors based on piezoelectric biomaterials**. A) Implantable piezoelectric sensor for assessing the progress of motor function recovery in nerve injury repair. (a) Schematic illustration of the PBPS sensor implanted in rats and the construction of the sensor. (b) Photographs showing the wireless assessment of rat motor function. (c) Real-time recorded output signals. Reproduced with permission.^336^ Copyright 2024, Wiley-VCH. B) Stretchable sensor based on a dl-alanine biocrystal network. (a) Optical image and photograph (inset) of a dl-alanine biocrystal network. (b) Mechanism of stretchability enabled by the truss-like network structure. (c) Stretchable sensor based on a dl-alanine network with a stretchable Ag nanowires electrode. (d) Increased voltage outputs of the stretchable sensor on a human hand under increased degrees of finger bending. Reproduced with permission.^278^ Copyright 2023, Springer Nature.

Studies also focused on combining highly piezoelectric but rigid biomolecular crystals with biodegradable polymers to achieve high-performance, flexible, and resorbable composite-based biosensors. For example, Hosseini et al. reported a biodegradable and flexible force sensor using a β-glycine-chitosan piezoelectric composite thin film.^72^ The sensor generated an output voltage of 190 mV in response to a 60 kPa pressure applied using a shaker, demonstrating high sensitivity (2.82 ± 0.2 mV kPa^–1^) and a fast response time (<100 ms). Yang et al. demonstrated an implantable biomechanical motion sensor using a heterogeneous structured flexible piezoelectric PVA-γ-glycine-PVA thin film.^74^ They implanted composite films protected by PLA encapsulation underneath the skin in the thigh and chest regions of rats and tested their biomechanical sensing performance. When the leg was gently stretched at approximately 1 Hz frequency, the piezoelectric sensor at the thigh generated an output voltage of 150 mV at the same frequency. The chest’s piezoelectric sensor produced a stable output voltage of 20 mV directly correlated with the rat’s respiration.

Stretchability is essential for sensors designed to interface with soft, dynamic biological tissues. Li et al. first reported a stretchable and biodegradable sensor using a stretchable piezoelectric dl-alanine fiber network.^278^ The stretchability was achieved by self-assembling dl-alanine microfibers in a continuous truss-like pattern on a stretchable substrate using dip-coating (Figure 12B-a, b). Its stretchability was several orders of magnitude higher than that of bulk crystals, and its piezoelectric properties remained intact, even when subjected to high tensile strains of up to 40%. Subsequently, they integrated the dl-alanine network with stretchable electrodes to fabricate an all-stretchable piezoelectric sensor (Figure 12B-c). The omnidirectional stretchability allowed the sensor to seamlessly conform to the surface of soft skin or tissues and to perceive irregular mechanical movements. Therefore, they mounted the stretchable sensor on a finger joint, which generated a piezoelectric response closely related to the degree of finger flexion (Figure 12B-d). Furthermore, even under repeated large angle bending, the sensor did not delaminate and maintained a stable voltage output of approximately 60 mV.^278^

### 11.2 Actuators and Ultrasonic Transducers

Piezoelectric actuators can generate small displacements with a strong force capacity by applying a voltage on the surface of a piezoelectric material. They have been widely used in ultraprecise positioning, step motors, resonance motors, and high-force manipulation. When a high-frequency voltage is applied, the piezoelectric actuator generates high-frequency vibrations, resulting in the production of ultrasonic waves. Ultrasonic transducers have found extensive use in medical applications such as extracorporeal ultrasound imaging and intracavitary ultrasound therapy.^337–339^ Ultrasonic waves can induce various effects on tissues, including mechanical disruption, cavitation, thermal effects, and sonoporation, leading to different therapeutic outcomes, such as localized tissue destruction or repair.

Blood-brain barrier opening for drug delivery has benefited from biodegradable piezoelectric transducers.^192,299^ The blood-brain barrier, composed of tight junctions between brain vascular endothelial cells, restricts the entry of most therapeutic drugs into brain tissue, posing a challenge for the treatment of brain disorders. Implantable ultrasound transducers offer a solution by enabling precise, low-intensity ultrasound treatments at specific locations deep within the brain, allowing for precise drug delivery by opening the blood-brain barrier, while minimizing the impact on surrounding brain tissue. Curry and Nguyen reported a biodegradable ultrasonic transducer composed of electrospun PLLA nanofibers that can be implanted in the brain to open the blood-brain barrier and safely be absorbed in the body without causing harm.^192^ The PLLA-based ultrasonic transducer was driven by a signal generator to produce continuous ultrasound waves at 1 MHz. Degradation experiments confirmed that the well-encapsulated transducers functioned normally in phosphate-buffered saline (PBS) at 37 °C for up to 8 days. Recently, the same research team has developed a new biodegradable transducer made from glycine-PCL (polycaprolactone) nanofiber membranes for in vivo ultrasound-mediated chemotherapy.^299^ When superficially implanted on the brain and activated by an external signal generator, the transducer induced the opening of the blood-brain barrier in deep brain regions, enabling the delivery of chemotherapy drugs to brain tissue (Figure 13A-a, b). They demonstrated that this biodegradable glycine-PCL-based ultrasonic transducer significantly improved the therapeutic efficacy and prolonged the survival of mice carrying U87MG orthotopic glioblastoma tumors.^299^

**Figure 13:**
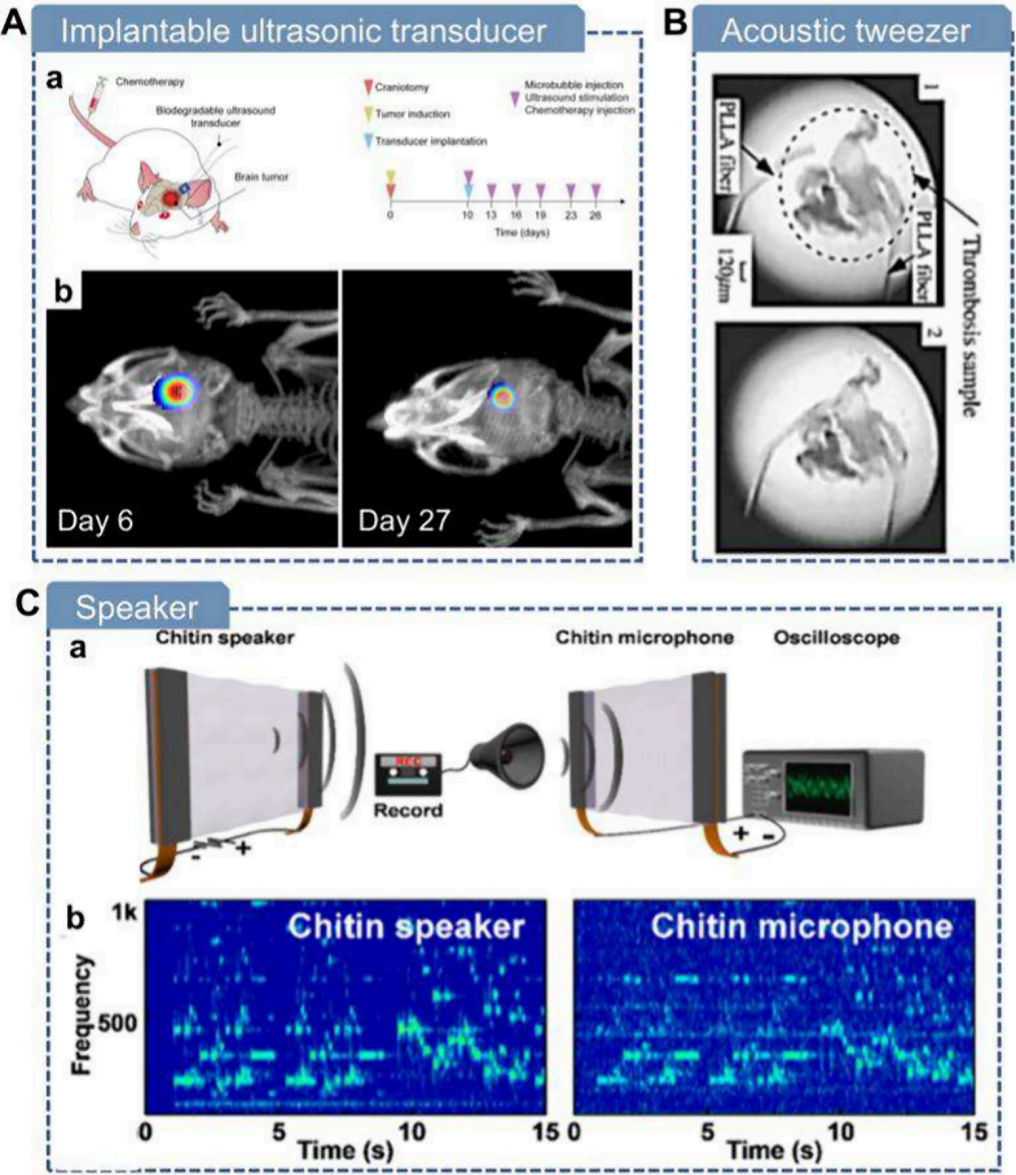
**Actuators and acoustic transducers based on piezoelectric biomaterials.** A) Biodegradable ultrasonic transducer for opening the BBB. (a) Schematic of the implantation of the transducer on the mouse brain. (b) Treatment results of GBM tumor delivery of chemotherapeutic drug to the brain facilitated by the biodegradable ultrasonic transducer. Reproduced with permission.^299^ Copyright 2023, American Association for the Advancement of Science. B) Optical images showing the use of piezoelectric PLLA tweezers to grasp and remove thrombosis samples. Reproduced with permission.^342^ Copyright 2006, Wiley-VCH. C) Chitin-based transducer. (a) Schematic of the chitin transducer as a speaker and a microphone. (b) Short-time Fourier transform (STFT) spectrograms from the chitin speaker and the chitin microphone. Reproduced with permission.^57^ Copyright 2018, Elsevier.

Acoustic tweezers based on biodegradable piezoelectric actuators are a critical application in personalized medicine. Tajitsu et al. utilized electrospun PLLA fibers to fabricate degradable electroactive tweezers with different structures for thrombus treatment in a series of studies.^340–343^ The PLLA fibers with a diameter of 40 μm could generate bending vibrations induced by the shear piezoelectric effect under AC voltages (50–300 V) with different frequencies (0.1–150 Hz). Leveraging these fibers, they designed tweezers that successfully achieved the functionality of grasping and extracting samples in a tiny region (Figure 13B).^342^ The tweezers with different structural designs could be implanted inside the body through minimally invasive surgery via a catheter and controlled externally by applying a voltage to perform grasping and thrombus clearance (Figure 13B).^342^

Piezoelectric biomaterial-based transducers have also been applied in sustainable electronics such as degradable speakers and piezoelectric micromachined ultrasound transducers (PMUTs). For instance, Kim et al. reported a biodegradable piezoelectric transducer made from chitin nanofiber films for use in speakers and microphones (Figure 13C-a).^57^ The chitin extracted from a squid pen was fabricated into transparent freestanding films with a predominant β-phase crystal structure via centrifugal casting and vacuum hot pressing. These chitin films exhibited reliable pressure response characteristics over a wide range of vibrational frequencies, making them suitable for acoustic actuators and sensors. Hence, a piezoelectric chitin speaker was manufactured and successfully played the sound of “Paganini Caprice No.1”. Additionally, the sound was recorded using a chitin microphone (Figure 13C-b). Joseph et al. introduced a silk-based PMUT fabricated by spin-coating biodegradable piezoelectric silk thin films on microfabricated silicon membranes.^344^ The silk films exhibited a supersmooth surface with a roughness of 2.84 and high adhesion strength. The silk-based PMUTs exhibited a center frequency of 76.59 kHz, a bandwidth of 2.44 kHz, and an electromechanical coupling efficiency of 7.07%.

### 11.3 Energy Harvesters (Nanogenerators)

Energy harvesters offer the potential of long-lasting, self-powered operation for low-power electronic devices without the need for batteries, thanks to their independence, sustainability, and maintenance-free characteristics. These devices can generate electrical energy from various forms of renewable resources, including thermal energy, solar energy, and mechanical energy.^345–350^ Among them, piezoelectric energy harvesters (PEHs) or nanogenerators (PENGs) are widely utilized as power sources for various medical devices and wearable electronics due to their high efficiency in converting mechanical energy to electrical energy, ease of manufacturing, and miniaturization capabilities.^7,41,351–355^ The incorporation of piezoelectric biomaterials further enhances these energy harvesters with excellent biocompatibility and biodegradability, making them highly suitable for implantable biomedical devices. These nanogenerators can harvest mechanical energy from various subtle movements of the body, such as muscle stretching, respiration, and heartbeat, and convert it into electrical energy. Additionally, they can be wirelessly recharged on demand using external ultrasound devices.^286^

#### 11.3.1 Natural Raw Material Based Piezoelectric Energy Harvesters

Biological piezoelectricity was first discovered in natural raw materials such as wool, wood, and bone. Recently, energy harvesters based on these animal and plant raw tissues have been actively studied. The piezoelectric behavior of these natural raw materials could be attributed to their intrinsic piezoelectric building blocks, such as collagen, cellulose, or chitin. For instance, Ghosh and Mandal directly utilized fish swim bladders to create a PENG that produced 10 V and 51 nA under finger tapping (1.4 MPa).^153^ These swim bladders were rich in naturally aligned collagen fibers, which served as the source of their piezoelectricity. Similar PENGs have been developed from fish scales,^356^ eggshell membranes,^357^ spider silk,^116^ and onion skins.^358^

Natural raw biomaterials have shown promising piezoelectric output, comparable to traditional materials, sparking research interest. However, caution must be exercised in interpreting these findings. Despite possessing piezoelectric building blocks, natural materials exhibit bulk structures with randomly or oppositely aligned piezoelectric macromolecules and non-piezoelectric components, which may cancel out macroscopic piezoelectricity. Upon re-evaluating these studies, we suspect that some reported outputs may also arise from other surface charge effects related to fabrication or measurement methods. Unlike the bulk effect of piezoelectricity, triboelectricity arises from contact or friction between electrode and material or excitation surfaces.^359–363^ While hybrid nanogenerators combining both effects can boost output,^364,365^ distinguishing the contribution of each mechanism is essential for accurate material evaluation.^359,361^ Nevertheless, these natural materials, often low-cost and easily processed, remain promising for nanogenerators, even after correcting for overestimated piezoelectric output.

#### 11.3.2 Processed Natural Material Based Piezoelectric Energy Harvesters

Further processing of these natural raw biological materials, including mechanical or chemical treatments, could be an effective approach to enhance the actual piezoelectric output of nanogenerators. For example, natural SIS was processed into ultrathin films (∼100 nm) with a monolayer collagen fiber network using the vdWE technique,^152^ making it ∼800 times thinner than the raw tissue. This mechanical exfoliation overcame piezoelectric cancellation from antiparallel domain alignment, enabling an effective piezoelectric thickness. A cantilever energy harvester with IP electrodes was fabricated from the SIS ultrathin film, yielding ∼250 mV output about three times higher than that of the raw SIS, highlighting enhanced piezoelectric performance via mechanical processing.^145^

Another feasible approach is to process natural biological materials through chemical or bioengineering methods to remove non-piezoelectric components and obtain porous piezoelectric structures. For example, Sun et al. developed a wood sponge nanogenerator by delignifying natural wood with hydrogen peroxide and acetic acid, followed by freeze-drying to transform its structure from honeycomb-like to spring-like layered.^366^ This enhanced compressibility, combined with the intrinsic piezoelectricity of crystalline cellulose, led to a significant increase in output. The device produced 0.69 V output, which was 85 times higher than that of raw wood (Figure 14A). In a follow-up study, they used fungal decay to remove lignin, producing a compressible piezoelectric wood sponge with a 55-fold output increase over natural wood, achieving up to 0.87 V and 13.3 nA from a single unit.^367^

**Figure 14:**
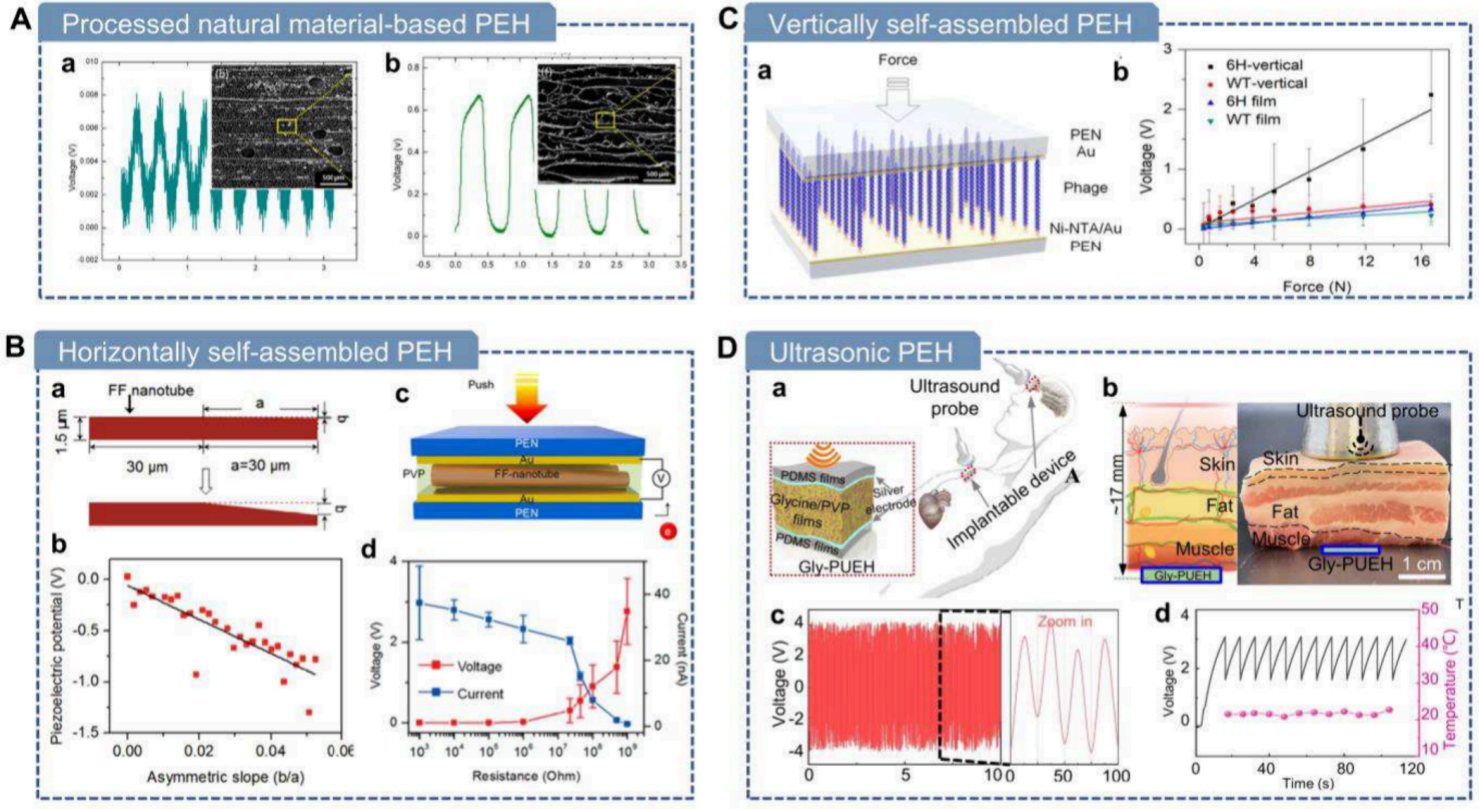
**Energy harvesters based on piezoelectric biomaterials.** A) Wood sponge PEH. Voltage output of (a) the native wood and (b) the wood sponge. Insets are the corresponding SEM images. Reproduced with permission.^366^ Copyright 2020, American Chemical Society. B) Horizontally assembled FF nanotube PEH. (a) The schematic diagram illustrates the dimensions of FF nanotubes. (b) FEM simulation results of piezoelectric potential as a function of slope of asymmetric FF nanotube by vertical pushing force. (c) PEH made with the horizontally assembled FF nanotubes. (d) Voltage and current output of the PEH depending on the load resistance. Reproduced with permission.^254^ Copyright 2018, American Chemical Society. C) Vertically assembled PEH. (a) Schematic diagram and (b) output voltages of the vertically assembled phage PEH. Reproduced with permission.^134^ Copyright 2019, American Chemical Society. D) Flexible β-glycine-PVP film based ultrasonic PEH. (a) Scheme of through-tissue wireless ultrasonic energy transfer by implanted ultrasonic PEH. (b) Schematic and picture of the ultrasonic PEH implanted under porcine tissue. (c) Voltage output of the ultrasonic PEH. (d) Charging curve of the 33 μF capacitor and the corresponding transmitted signals. Reproduced with permission.^308^ Copyright 2024, American Association for the Advancement of Science.

#### 11.3.3 Horizontally Self-Assembled Piezoelectric Energy Harvesters

Unlike raw bulk biomaterials with their nature-defined structures, the bottom-up self-assembly of piezoelectric biomolecules offers a promising strategy to boost energy harvester output. A common approach involves forming horizontally arranged superstructures with IP polarization. For instance, for example, a pioneering virus-based nanogenerator was made from IP self-assembled columnar bacteriophage films, producing 6 nA and 400 mV outputs.^21^ Due to the D6 or C6 symmetry and antiparallel IP polarization, vertical piezoelectricity was not expected. Hence, researchers proposed that the measured vertical piezoelectricity might be related to the space-dependent piezoelectric matrices and stress/stress-gradient induced polarization.^21^

Similar approaches have also been employed to fabricate large-scale, unidirectionally polarized, horizontally aligned FF nanotube PEH (Figure 14B-a, b).^254^ Although lacking inherent vertical piezoelectricity, the asymmetric shape of FF nanotubes enabled the effective conversion of axial stress into shear deformation, as confirmed by simulations. A strong linear correlation was observed between nanotube slope and output (Figure 14B-c). Under a 42 N force, FF peptide-based nanogenerators generated up to 2.8 V, 37.4 nA, and 8.2 nW, which are enough to power several LCD panels (Figure 14B-d).^254^

#### 11.3.4 Vertically Self-Assembled Piezoelectric Energy Harvesters

While horizontally aligned self-assembled structures have enabled OOP energy harvesting, fully vertically aligned structures with oriented polarization remain most desirable. To achieve this, methods such as templating and electric field-assisted self-assembly have been explored. For instance, Shin et al. used enforced infiltration into an AAO template to vertically align bacteriophage nanorods, yielding approximately 2.6 times higher output than horizontal assemblies.^133^ However, the antiparallel phage orientation persisted. To address this, Lee et al. combined genetic engineering with PDMS templates (Figure 14C-a), producing an unidirectionally polarized phage nanogenerator that reached 2.8 V, 120 nA, and 236 nW under 17 N force (Figure 14C-b).^134^ Similarly, Nguyen et al. applied an electric field during self-assembly to align FF microrods, achieving 1.4 V, 39.2 nA, and 3.3 nW cm^–2^ from the resulting PENG.^310^

Still, non-piezoelectric templates and structural nonuniformity hindered piezoelectric output. To address this, Wang et al. fabricated a heterostructured PVA−γ-glycine film via interfacial hydrogen bonding to control crystal phase and orientation, achieving uniform wafer-scale piezoelectricity.^74^ The resulting energy harvester produced up to 4.1 V and 360 nA under a 30 N pulsed force. However, due to the inclined nucleation interface, the polarization direction ([001]) was tilted, limiting output to ∼50% of γ-glycine’s intrinsic potential. To improve alignment, the team later relocated nucleation to flat interfaces, increasing the d_33_ to 6.13 pC N^–1^ and boosting voltage to 6.1 V.^76^

Zhang et al. first achieved both optimal OOP polarization and a compact, uniform structure in a single-component β-glycine crystal film.^53^ XRD confirmed that the (020) piezoelectric axis aligned perpendicularly to the surface, while PFM showed well-aligned ferroelectric domains. The resulting nanogenerator delivered an exceptional performance of 14.5 V OCV, 4 μA current, and 3.61 μW cm^–2^ power density.^53^ However, these crystal films remain rigid and fragile, requiring integration with flexible biopolymers or substrates. Despite these limitations, the active self-assembly method demonstrated scalable fabrication of highly aligned biofilms and offers a promising route for high-output piezoelectric biomaterials.^375^

#### 11.3.5 Ultrasonic Piezoelectric Energy Harvesters

Most of the nanogenerator demonstrations mentioned above were conducted in vitro under impact testing and have not fully leveraged the unique properties of biomaterials for biomedical applications. For in vivo use, piezoelectric nanogenerators can potentially power implantable devices such as pacemakers, defibrillators, and neurostimulators. However, random low-frequency biomechanical movements typically generate insufficient power, especially given the lower output of piezoelectric biomaterials compared with that of inorganic ceramics or synthetic polymers. External ultrasound offers a safe, wireless method for on-demand charging of implantable nanogenerators via energy transfer through acoustic waves.^286,368^

Li et al. first introduced a biodegradable ultrasonic piezoelectric energy harvester (Gly-PUEH) based on glycine–PVP thin films for wireless charging of transcutaneous implants (Figure 14D).^308^ When activated by an ultrasound probe, the device beneath porcine tissue generated ∼3.6 V, 10 μA, and a high-power density of ∼35 μW cm^–1^, which are sufficient to recharge small implants like pacemakers and defibrillators, which typically require less than 10 μW. The team also demonstrated Gly-PUEH powering an ex vivo temperature sensor: harvested energy was stored in a 33 μF capacitor with an under-voltage lockout to drive a wireless transmitter, enabling data transmission every 8 s (Figure 14D-b). In addition, wireless ultrasound triggering successfully lit a red LED, showing the potential for optogenetic stimulation. Notably, the voltage output remained stable over 20 days, indicating excellent durability. Overall, this study provides the first proof-of-concept that piezoelectric biomaterials can enable sustainable, wireless energy systems for implantable medical devices.

### 11.4 Filtration

N95 and surgical masks are widely used to prevent the spread of contagious viruses, offering protection by filtering industrial particles and reducing air pollution exposure. However, their disposable and non-biodegradable nature leads to increasing nonrecyclable waste, posing serious environmental concerns. In addition, surface-induced charges on these masks tend to dissipate quickly, resulting in a reduced filtration efficiency. This degradation increases the infection risk for users during prolonged or continuous wear.

Recent efforts have aimed to enhance and sustain electrostatic filtration using electroactive filtering materials.^369–372^ However, most of these materials remain non-biodegradable, raising ongoing environmental concerns due to persistent waste.^373^ Piezoelectric materials offer an alternative option for filtering materials to achieve improved and stable charge adsorption effects, thanks to their robust electromechanical properties and ease of manufacturing. Nguyen et al. proposed the use of PLLA nanofibers to create a biodegradable air filtration membrane (Figure 15 A).^374^ The PLLA nanofiber filter effectively removed ultrafine particles (PM 2.5 up to 99%, PM 1.0 up to 91%) and provided a favorable pressure drop of approximately 91 Pa for human respiration, thanks to the nanofiber structure and sustainable piezoelectric charges generated. The SEM images before and after filtration demonstrate the effectiveness of static adsorption from piezoelectric nanofibers (Figure 15 B).

**Figure 15:**
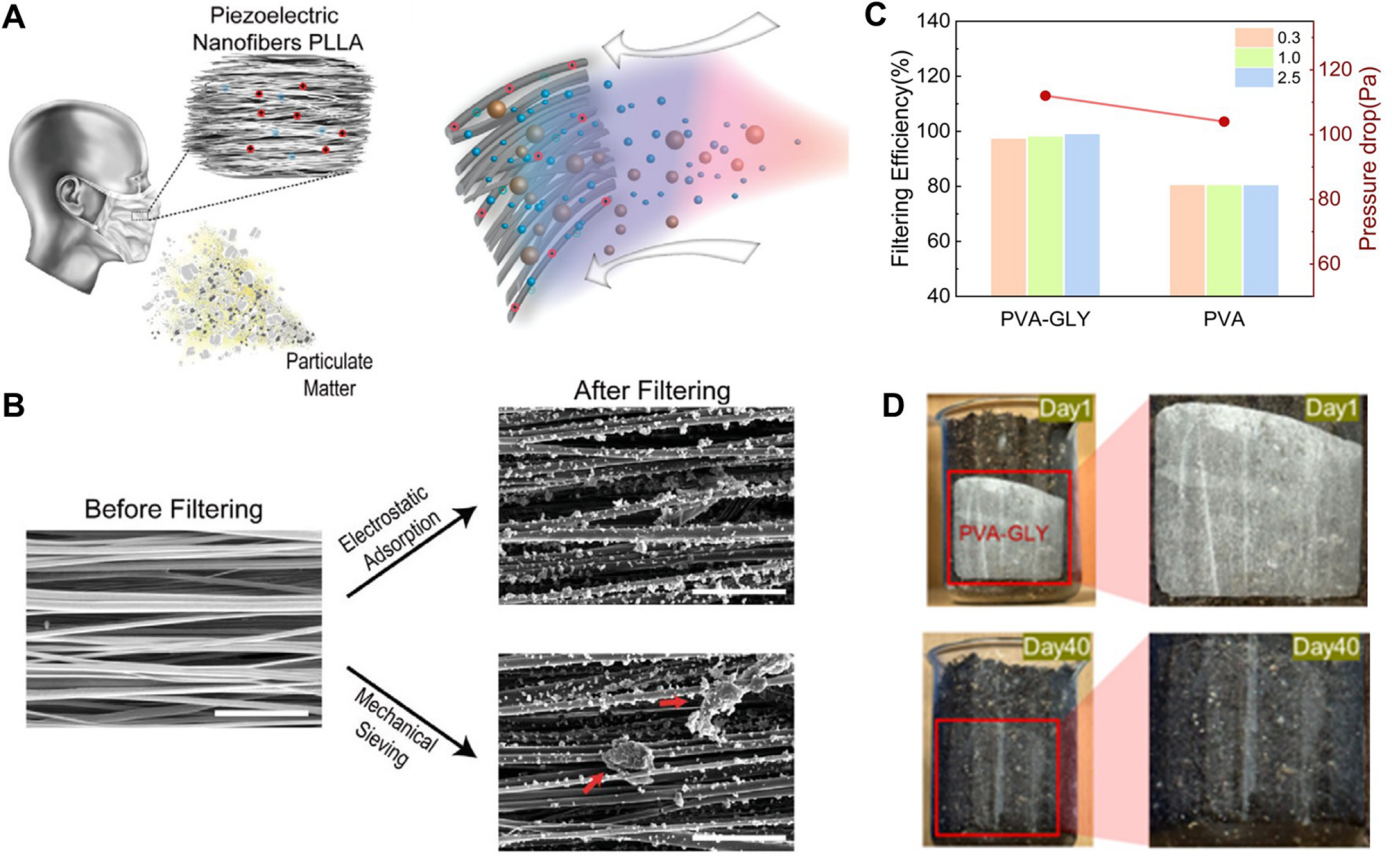
**Filtration based on piezoelectric biomaterials.** A) Photograph and schematic of the self-charging face mask using a PLLA nanofiber membrane as the filter. B) Working principle and two main filtration mechanisms of the mask: mechanical filtration of large particles and electrostatic adsorption of small particles. Reproduced with permission.^374^ Copyright 2006, Wiley-VCH. C) Comparison of the filtration performance of PVA and β-glycine-PVA nanofiber membrane filters. D) Degradation behavior of the β-glycine-PVA nanofiber membranes. Reproduced with permission.^301^ Copyright 2024, Elsevier.

Although PLLA is biodegradable, the high cost and production process involving toxic solvents still limit its widespread use. Wang et al. developed a low-cost, eco-friendly strategy using piezoelectric glycine mixed with PVA to create a self-charging, biodegradable air filtration membrane (Figure 15C, D).^301^ The electrospun nanofibers exhibited strong piezoelectricity due to highly oriented β-glycine crystals embedded in the flexible PVA shell. This electrostatic effect enabled high filtration efficiencies — 98.8%, 97.9%, and 97.1% for 2.5 μm, 1.0 μm, and 0.3 μm particles, respectively — at a low-pressure drop of ∼112 Pa (Figure 15C). The mask completely degraded in only a few weeks in the composite soil, while the current surgical mask took up to 20–30 years (Figure 15D).^301^ Furthermore, the entire PVA–Gly film lifecycle from fabrication to decomposition avoids toxic materials or solvents, aligning with sustainability principles.

### 11.5 Tissue Engineering

Tissue engineering aims to restore or replace damaged tissues and organs by integrating cells, scaffolds, and bioactive cues. Within this landscape, piezoelectric biomaterials — which transduce mechanical inputs into localized electrical signals — have gained prominence for their unique electromechanical coupling, enabling self-powered control of cell behavior and tissue repair. Recent studies highlight the broad utility across bone, cartilage, skin, and peripheral nerve applications.

#### 11.5.1 Piezoelectric Electrical Stimulation Mechanism

As early as 1892, Julius Wolff discovered that the bones of healthy humans or animals adapt and change shape in response to the load imposed on them, a phenomenon known as Wolff’s law.^31,375^ This law explains that the density and structure of the bone rely on the variations in the forces exerted on it. If the load on a specific bone increased, then the bone would gradually become stronger to withstand that load. Subsequently, it was found that bones exhibit inherent piezoelectric effects, generating electrical charge under mechanical stress, which plays a crucial role in bone development and remodeling.^16,17,144^ The discovery of bone piezoelectricity has sparked research into the physiological significance of bio-piezoelectricity and the development of bio-piezoelectric scaffolds or nanomaterials for tissue regeneration. These implanted bio-piezoelectric materials can generate piezoelectric potential differences through subtle body movements, cell migration, or external ultrasound stimulation. In many instances, electrical stimulation has the capability to modulate voltage-gated ion channels (e.g., calcium channels), to regulate intracellular ion levels, thereby promoting cell proliferation and differentiation.^10,376,377^ Elevated levels of intracellular Ca^2+^ concentration were shown to activate calmodulin, calcineurin, and calcineurin dephosphorylates nuclear factor, which further translocates to the cell nucleus to enhance the expression of growth factors. Hence, piezoelectric biomaterials offer an effective platform for stimulating the regeneration or remodeling of intricate biological tissues, including bone and cartilage.

#### 11.5.2 Cell Attachment and Culture

Bio-piezoelectric scaffolds have been highly effective interfaces for cell adhesion and cultivation by modulating the mechanical and electrical environment. Smith et al. explored the application of PLLA nanotubes as bio-piezoelectric interfaces for cell culture and investigated the impact of mechanical modulus, surface charge, and piezoelectricity of nanotubes on cell behavior.^378^ By controlling the crystallinity of the nanotubes, they were able to modulate the level of cell adhesion. The findings suggested that the electromechanical interaction between piezoelectric biomaterials and cells can generate localized electric fields, thereby facilitating cell proliferation and differentiation through repetitive electrical stimulation. Tai et al. optimized a stem cell engineering platform using electrospun PLLA nanofibers.^379^ Modulating the bio-piezoelectricity in these nanofibers had a significant influence on the differentiation behavior of stem cells, exhibiting cell type-specific effects. Specifically, the orthogonal piezoelectric effect enhanced neurogenesis, whereas the shear piezoelectric effect promoted osteogenesis.

#### 11.5.3 Bone Regeneration

Bone regeneration is a key application area for piezoelectric biomaterials.^380^ Han et al. recently introduced a natural collagen scaffold (PiezoCol) that preserves collagen’s native tertiary structure — and thus its intrinsic piezoelectricity — showing that piezoelectric cues alone can markedly potentiate osteogenesis in vivo (Figure 16A).^381^ Under low-intensity ultrasound, PiezoCol promoted robust bone-like tissue formation while simultaneously enhancing angiogenesis and neurogenesis; mechanistic analyses implicated activation of the integrin/PI3K–Akt axis, and even in the absence of ultrasound, everyday motion appeared sufficient to provide weak yet beneficial piezoelectric stimulation. Chernozem et al. proposed a hybrid biocomposite material based on piezoelectric PHB/PHBV polymer as an effective scaffold platform for stimulating bone tissue growth through functionalization with an inorganic phase.^382^ Ultrasound mineralization of PHB and PHBV scaffolds containing biocompatible CaCO_3_ transformed their surface from hydrophobic (for nonmineralized) to hydrophilic (for CaCO_3_ mineralized), notably enhancing the adhesion and proliferation of osteoblasts and improving the hydroxyapatite formation behavior. Gorodzha et al. conducted a comparative study on three different bone scaffolds made of electrospun PCL, PHBV, and a composite material of PHBV with hydroxyapatite containing polyhydroxybutyrate (PHBV-Si-HA).^383^ In vitro studies demonstrated that the piezoelectric PHBV and PHBV-Si-HA scaffolds exhibited superior calcium deposition ability compared to the non-piezoelectric PCL scaffold, while the PHBV-Si-HA scaffold exhibited the highest adhesion and differentiation capability. Das et al. reported a piezoelectric PLLA fiber-based biodegradable scaffold that utilized ultrasound-mediated electrical stimulation to drive bone regeneration.^195^ In vitro experiments demonstrated a significant enhancement in the differentiation of stem cells into osteoblasts using this system. By implanting the PLLA scaffolds into bone defects in mice, controlled surface charges were generated by applying external ultrasound to the scaffold surface, leading to improved osteogenesis and the promotion of bone regeneration.

**Figure 16:**
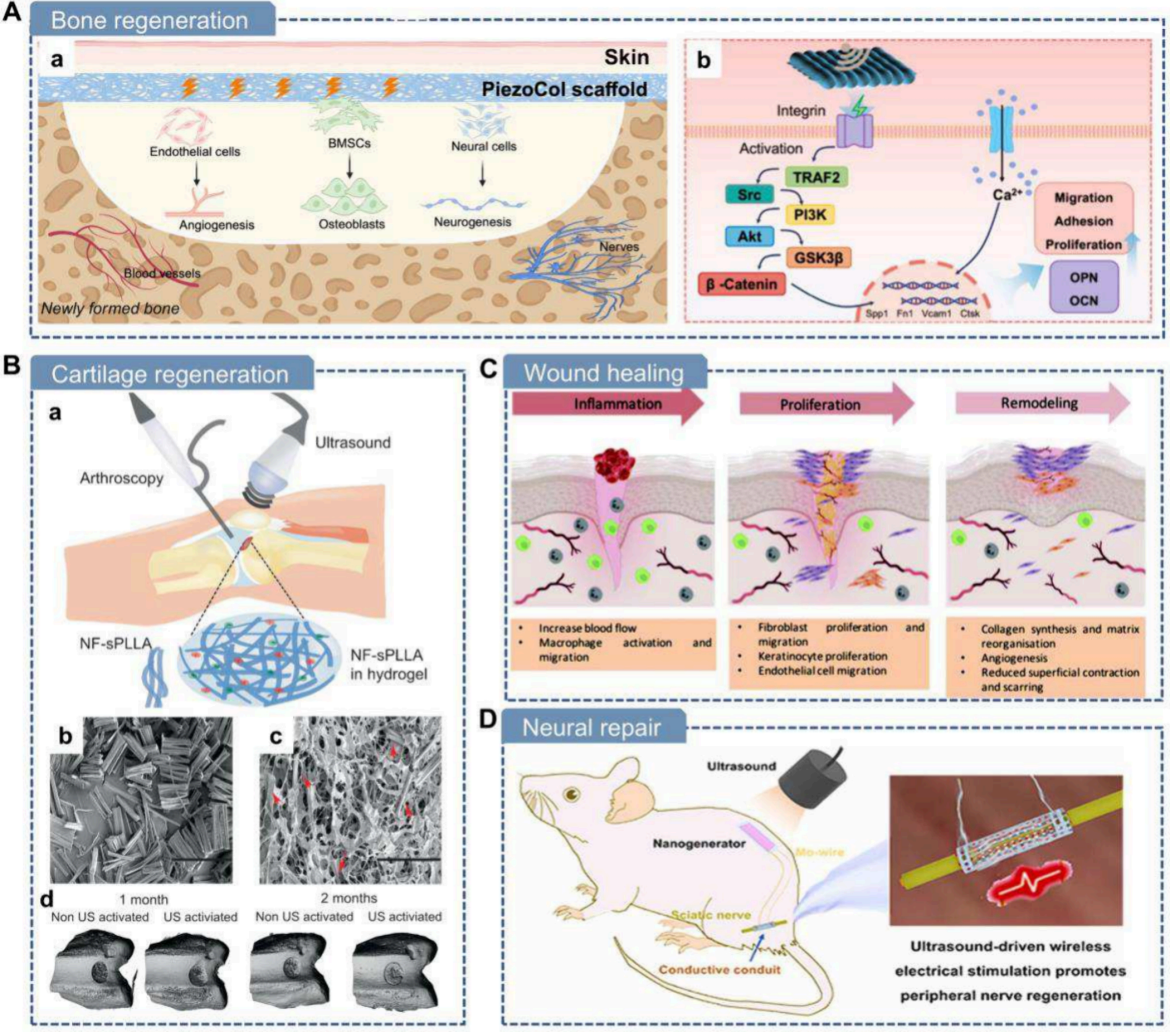
**Tissue engineering based on piezoelectric biomaterials**. A) Bone regeneration based on natural piezoelectric collagen scaffolds. (a) Schematic showing bone regeneration after ultrasound treatment of the PiezoCol collagen scaffolds implanted under the skin. (b) Comprehensive schematic of the mechanism of bone formation via piezoelectric stimulation. Reproduced with permission.^381^ Copyright 2025, Elsevier. B) Cartilage regeneration based on piezoelectric hydrogels. (a) Schematic of the injected piezoelectric hydrogel for osteoarthritis patients. SEM image of PLLA short nanofibers (b) after sectioning (scale bar: 40 μm) and (c) in the dried collagen scaffold (scale bar: 40 μm). (d) Reconstruction of the bone on femurs using μ-CT. Reproduced with permission.^295^ Copyright 2023, Springer Nature. C) Schematic of typical three stages of wound healing. Reproduced with permission.^385^ Copyright 2021, Royal Society of Chemistry. D) Schematic of nerve regeneration based on a biodegradable PENG activated by ultrasound. Reproduced with permission.^386^ Copyright 2022, Elsevier.

#### 11.5.4 Cartilage Regeneration

Piezoelectric materials have also shown promise in cartilage regeneration.^377,384^ Recently, Liu et al. engineered a biodegradable piezoelectric scaffold using PLLA nanofibers. This scaffold acted as a battery-free electrical stimulator, effectively promoting chondrogenesis and facilitating the regeneration of cartilage tissue.^197^ The PLLA scaffold generates controllable piezoelectric charges when subjected to force or joint loading, promoting extracellular protein adsorption, facilitating cell migration or recruitment, inducing endogenous TGF-β through calcium signaling pathways, and improving cartilage formation and regeneration in vitro and in vivo. They implanted the piezoelectric PLLA scaffold into the medial condyles of rabbit femurs with critical-sized osteochondral defects. The movement of the rabbits induced joint loading, thereby resulting in the generation of piezoelectric charges in the scaffold. After two months of movement, the rabbits demonstrated notable hyaline cartilage regeneration and achieved full recovery of the cartilage, as evidenced by the abundant presence of chondrocytes and type II collagen. However, the implantation of these piezoelectric scaffolds requires invasive surgical procedures and may lead to potential complications, such as infection and inflammation. Considering this, Vinikoor et al. introduced an injectable, ultrasound-activated, piezoelectric biomaterial-based hydrogel for cartilage regeneration (Figure 16B).^295^ The hydrogel was prepared by mixing cryosectioned PLLA short nanofibers with a collagen matrix. It could be inoculated into articular cartilage defects via injection to avoid invasive implantation surgery. The piezoelectric hydrogel can be remotely activated by ultrasound, generating electrical stimulation to promote the healing of severe cartilage defects and effectively treat osteoarthritis.

#### 11.5.5 Wound Healing

Wound healing is another major focus in tissue regeneration research.^385,387,388^ Current wound healing therapies primarily focus on passive healing processes, aiming to reduce wound infection and enhance tissue rehydration at the wound site. In contrast to passive treatment strategies, piezoelectric biomaterials, as a type of bioactive material, have the potential to actively accelerate wound healing by generating internal electric fields in response to mechanical stimulation. Recent research has revealed distinct impacts of electrical stimulation on wound healing, which encompass three intersecting phases: inflammation, proliferation, and remodeling. During the inflammation phase, electrical stimulation has the capacity to enhance blood flow and tissue oxygenation, inhibit bacterial growth, and reduce wound edema. During the proliferation phase, electrical stimulation promotes fibroblast proliferation, stimulates angiogenesis, and enhances collagen deposition. During the remodeling phase, electrical stimulation plays a significant role in promoting collagen maturation and remodeling, thereby expediting wound contraction and enhancing wound tensile strength.

For instance, Goonoo et al. proposed a 20/80 polydioxanone (PDX)/PHBV core–shell scaffold with balanced wetting, piezoelectric, and mechanical performance for scarless wound regeneration (Figure 16C).^385^ The piezoelectric scaffold promoted blood vessel formation and keratinocyte growth and reduced inflammatory reactions. The favorable mechanical properties of the blend scaffolds led to decreased fibroblast-mediated contraction after 3 weeks, as fibroblasts had minimal impact on fiber deformation. Das et al. utilized a piezoelectric PLLA nanofiber matrix for skin-wound healing.^388^ External ultrasound was applied to induce well-controlled surface charges of different polarities on the piezoelectric scaffold, where negative surface charges inhibited bacterial growth, while positive surface charges promoted skin regeneration. They validated the scaffolds in an in vivo critical-size skin wound mouse model, where the scaffold effectively induced rapid skin regeneration. In a recent study, Xue et al. developed a flexible, biodegradable, and wireless piezoelectric-ultrasound device using highly aligned γ-glycine/PVA films, which accelerated wound healing by ∼40% and naturally degraded after use.^389^ Its stable ultrasound-induced output (∼220 mV mm^–1^) and serpentine flower-shaped electrodes enabled effective electrotherapy in wound models, demonstrating a strong potential for transient, battery-free medical applications.

#### 11.5.6 Neural Repair

Neurological disorders, including nerve trauma and neurodegenerative diseases, have significant implications for both disability and mortality rates. Studies have indicated that electrical stimulation can induce the upregulation of brain-derived neurotrophic factor (BDNF) and its high-affinity receptor tropomyosin receptor kinase B (TrkB), in neuronal cells. This upregulation occurs through a calcium-dependent mechanism and leads to increased expression of regeneration-associated genes by upregulating cyclic adenosine monophosphate (cAMP) pathways. These molecular activities ultimately facilitate axon bursting and prevent the collapse of growth cones. Therefore, the utilization of piezoelectric biomaterials can effectively enhance nerve regeneration by generating electrical stimulation in response to mechanical stimuli applied to injured nerves.^10,390,391^

Wu et al. proposed a potassium sodium niobate (KNN) nanowire-PLLA-PHBV-based biodegradable PENG for repairing peripheral nerve injuries (Figure 16D).^386^ The implanted PENG was wirelessly activated by external ultrasound, generating an inherent electric field that was conveyed to the surrounding nerves through biodegradable conductive conduits. Using a rat sciatic nerve injury model, they validated that ultrasound-induced piezoelectric stimulation greatly promoted nerve regeneration through nerve functional recovery analysis, histological evaluation, and microstructural analysis. Yang et al. reported a self-powered and conductive carbon nanotube@gelatin methacryloyl/PLLA scaffold for peripheral nerve regeneration.^294^ In vitro experimental data demonstrated that the scaffold greatly enhanced the adhesion and elongation of Schwann cells while promoting axonal growth and neurite quantity in dorsal root ganglia. After the scaffold was implanted at a 10 mm sciatic nerve defect in rats for 12 weeks, enhanced myelin sheath formation and axonal growth were observed, significantly facilitating peripheral nerve regeneration. Nevertheless, although polymer matrices such as PLLA and PHBV are biodegradable, the long-term in vivo degradability and clearance of KNN nanowires and carbon nanotubes remain insufficiently characterized; translation should therefore proceed with caution, supported by rigorous toxicology and degradation studies. Li and Ren developed a multilayer film composed of a PLLA piezoelectric thin film and PLLA nanofibers for the growth of neuron-like cells.^392^ They found that the multilayer film significantly outperformed individual thin films or nanofibers in enhancing directed cell growth. Chen et al. reported a fully biodegradable, amino-acid–based nerve guidance conduit (NGC) composed of aligned PCL/β-glycine nanofibers that generate ES under low-frequency mechanical vibration (e.g., a massage gun).^393^ In a 10 mm rat sciatic nerve defect, the piezoelectric NGC drove Schwann-cell myelination and neurite outgrowth and achieved ∼99% motor recovery and ∼96% nerve-conduction restoration — outcomes comparable to those of autografts — while avoiding high-frequency ultrasound heating concerns.

## 12 Emerging Medical Applications of Piezoelectric Materials

In the previous section, we summarized the development of piezoelectric biomaterials and highlighted their potential in bioelectronics and biomedical applications. While these biomaterials offer advantages such as biocompatibility and degradability, their relatively recent emergence, limited electromechanical performance, and processing challenges have thus far restricted their translation into many conventional application domains where inorganic piezoelectrics dominate. To complement this perspective, this section turns to the broader field of conventional piezoelectric materials and examines several cutting-edge medical applications that are currently being explored. Particular emphasis will be placed on neuromodulation, piezocatalytic generation of reactive oxygen species (ROS), and mechanochemical synthesis approaches that leverage piezoelectric phenomena. Together, these examples illustrate how advanced piezoelectric platforms are expanding beyond traditional roles in sensing and actuation to open new opportunities for therapeutic intervention and biomedical innovation.

### 12.1 Neuromodulation

Neural stimulation technologies are reshaping both basic neuroscience and clinical practice by enabling external control of neuronal activity. Traditional electrode-based stimulators have provided important therapeutic benefits, yet their invasiveness, reliance on implanted batteries, and long-term stability issues significantly limit their broader application. Recent advances in piezoelectric and ultrasound-based technologies have enabled the design of wireless, flexible, and battery-free stimulators that can target the nervous system with higher precision and reduced invasiveness. These efforts are rapidly expanding the landscape of neuromodulation across the central nervous system,^394–398^ peripheral nerves,^399–402^ and even gut–brain communication.^403^

#### 12.1.1 Deep Brain Stimulation

Deep brain stimulation (DBS) has long been an effective therapy for neurological disorders, but conventional electrode-based systems remain hindered by invasiveness, limited spatial selectivity, and the need for battery replacement. Recent piezoelectric–ultrasound technologies have expanded the DBS toolkit with diverse strategies that differ in materials, device architecture, and stimulation mechanisms. One representative approach is the PUEH device based on Sm-doped Pb­(Mg1/3Nb2/3)­O_3_–PbTiO_3_ (Sm-PMN-PT) single crystals (Figure 17A).^404^ This flexible implant exhibits outstanding energy conversion efficiency, achieving power densities of up to 1.1 W cm^–2^ in vitro. When implanted in rat brains, it produced sufficient output under safe ultrasound intensities to stimulate the periaqueductal gray via integrated electrodes, resulting in both electrophysiological activation and behavioral analgesia. Another strategy involves dual-frequency lead-free ultrasound implants, which integrate porous piezoelectric KNN composites onto flexible circuits.^396^ Operating at two resonance frequencies (1 and 3 MHz), the system can efficiently harvest energy while delivering biphasic, charge-balanced pulses. This feature is critical to avoiding electrode degradation and tissue damage associated with monophasic stimulation.

**Figure 17:**
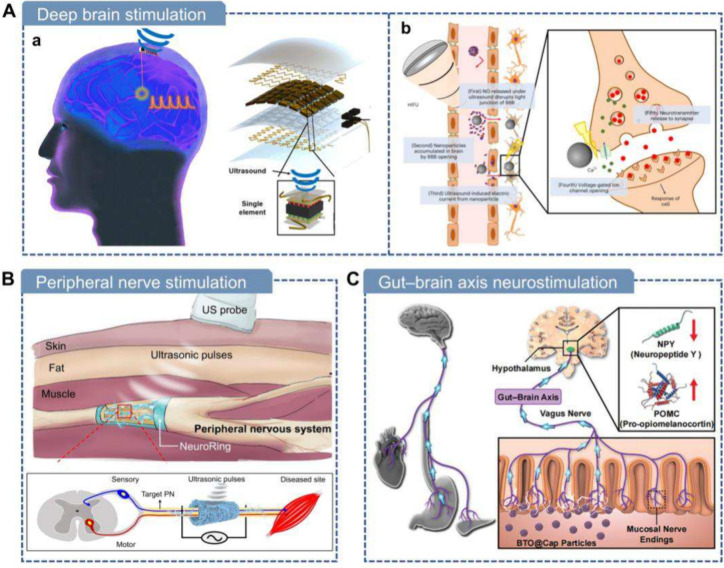
**Neuromodulation based on piezoelectric materials.** A) Deep brain stimulation (DBS). (a) Schematic of the Sm-PUEH device for DBS. Reproduced with permission.^404^ Copyright 2022, American Association for the Advancement of Science. (b) Schematic of the nanoparticle for blood–brain barrier opening and DBS under ultrasound application. Reproduced with permission.^397^ Copyright 2022, Springer Nature. B) Schematic of peripheral nerve stimulation based on a soft ferroelectret ring. Reproduced with permission.^402^ Copyright 2023, Springer Nature. C) Schematic of noninvasive and self-powered gut-brain axis neurostimulation based on BaTiO_3_ piezoelectric particles. Reproduced with permission.^403^ Copyright 2024, Wiley-VCH.

A distinct line of development is represented by ImPULS, a flexible micromachined transducer array based on KNN.^395^ Unlike electrode-driven systems, ImPULS enables direct ultrasonic stimulation, generating localized acoustic pressure (∼100 kPa) within brain tissue to activate the hippocampal and nigrostriatal neurons. In vivo studies showed time-locked c-Fos expression and dopamine release, underscoring the potential of purely acoustic, nonelectrode neuromodulation in deep brain circuits. Recently, a nonimplantable route has been demonstrated using systemically delivered BTO nanoparticles.^397^ Activated by focused ultrasound, the particles generate local electric fields and simultaneously release nitric oxide, which transiently opens the blood–brain barrier to facilitate nanoparticle accumulation. This combined mechanism enhanced dopamine release and alleviated Parkinsonian symptoms in mice without apparent toxicity, offering a radically different paradigm for noninvasive DBS.

Together, these examples demonstrate how wireless, biocompatible, and highly localized DBS platforms are transitioning from a proof-of-concept toward translational relevance. They offer improved spatial precision, tunable stimulation parameters, and longer functional lifetimes compared to conventional electrode systems. Future efforts will need to address chronic biostability, integration with closed-loop recording interfaces, and translation into large animal and human models.

#### 12.1.2 Peripheral Nerve Stimulation

Peripheral nerve stimulation (PNS) provides an important route for modulating motor activity, treating inflammatory disorders, and restoring neuromuscular function. Traditional PNS devices are often rigid and bulky, powered by implanted batteries or wired transcutaneous connections, which limits long-term stability and raises concerns about tissue damage, inflammation, and patient comfort. Recent advances in piezoelectric and ultrasound-driven technologies have enabled a new class of wireless, soft, and conformal stimulators that interface more naturally with peripheral nerves.

Li and colleagues reported the NeuroRing, a soft ferroelectret-based ultrasound receiver designed as a flexible ring that gently wraps around peripheral nerves (Figure 17B).^402^ By harvesting incident ultrasound, the device generates localized electrical pulses without the need for rigid electrodes or batteries. Demonstrated in a colitis model, NeuroRing successfully modulated sacral splanchnic nerve activity and alleviated disease symptoms, highlighting its potential as a safe and biofriendly platform for neuromodulation. In parallel, Zhang et al. developed a conch-inspired piezoelectric stimulator, where a spiral resonator efficiently converted acoustic signals — such as those from a mobile phone — into electrical pulses to stimulate the sciatic nerve in mice, producing reproducible muscle activation under a fully wireless scheme.^398,400^

Collectively, these studies illustrate a transition in PNS from rigid, battery-powered electrodes to soft, wireless, and programmable bioelectronic interfaces. By leveraging piezoelectric and ferroelectret materials, these devices not only achieve efficient ultrasound energy harvesting but also conform mechanically to delicate nerve tissue, offering new opportunities for long-term, minimally invasive peripheral neuromodulation.

#### 12.1.3 Gut–Brain Axis Neurostimulation

The gut–brain axis is increasingly recognized as a critical pathway for regulating appetite, metabolism, and even emotional states. Vagus nerve stimulation (VNS), delivered via cervical electrodes, has been clinically tested for epilepsy, depression, and obesity, but invasive implantation has limited its widespread use. Noninvasive, self-powered alternatives are therefore highly desirable.

Mac et al. developed orally ingested self-powered stimulators for targeting the gut–brain axis. These devices consist of BTO piezoelectric particles conjugated with capsaicin, a ligand for TRPV1 receptors (Figure 17C).^403^ After ingestion, the particles specifically bind to the TRPV1-expressing gastric nerve endings. Natural stomach peristalsis provides the mechanical input to generate piezoelectric electrical pulses, which stimulate vagal afferents and enhance hypothalamic satiety signals. In diet-induced obese mice, daily ingestion over 3 weeks produced significant reductions in body weight and improvements in metabolic markers, without detectable toxicity. This platform stands out as a noninvasive, molecularly targeted, and self-powered neuromodulation strategy. Unlike surgically implanted stimulators, they leverage natural physiological processes (gastric motility) to power stimulation and achieve specificity through ligand–receptor interactions. The concept opens the door to a new category of “ingestible bioelectronics” that could be extended to other vagal-mediated conditions, including mood disorders, inflammation, and gastrointestinal dysfunctions.

### 12.2 Piezocatalytic ROS Medicine

Piezocatalysis represents an emerging catalytic modality that couples the piezoelectric effect with redox chemistry to generate ROS. Unlike conventional photocatalysis, which is limited by shallow light penetration and external illumination requirements, piezocatalysis can be activated by widely available mechanical inputs such as ultrasound, vibration, or body motion, enabling ROS generation in deep tissues and diverse aqueous environments. The resulting ROS — including hydroxyl radicals (•OH), superoxide anions (•O_2_^–^), and hydrogen peroxide — possess high oxidative reactivity and can be harnessed for biomedical interventions ranging from cancer therapy to antibacterial treatment and environmental purification.

#### 12.2.1 Mechanism of Piezocatalysis

The fundamental basis of piezocatalysis lies in the strain-induced polarization of non-centrosymmetric piezoelectric crystals, which creates an internal electric potential and drives charge redistribution at the catalyst surface. To date, two major mechanistic models have been established — energy band theory (Figure 18A) and the screening charge effect (Figure 18B).^405,406^ According to energy band theory, mechanical deformation modulates the conduction and valence bands of piezoelectric materials, facilitating charge carrier separation and enabling redox reactions at the solid–liquid interface. In this framework, electrons can reduce dissolved oxygen to superoxide anions (•O_2_^–^), while holes can oxidize water molecules to produce hydroxyl radicals (•OH). By contrast, the screening charge effect emphasizes the dynamic adsorption and release of external charges at the material–electrolyte interface. Under periodic stress (e.g., ultrasound), surface polarization charges are alternately screened and unbalanced, driving continuous electron transfer reactions that yield ROS. Both models are supported by experimental evidence and offer reasonable explanations of piezocatalysis from different perspectives. They highlight distinct aspects of charge generation and transfer and can be applied to interpret piezocatalytic behavior under different material systems and operating conditions.

**Figure 18:**
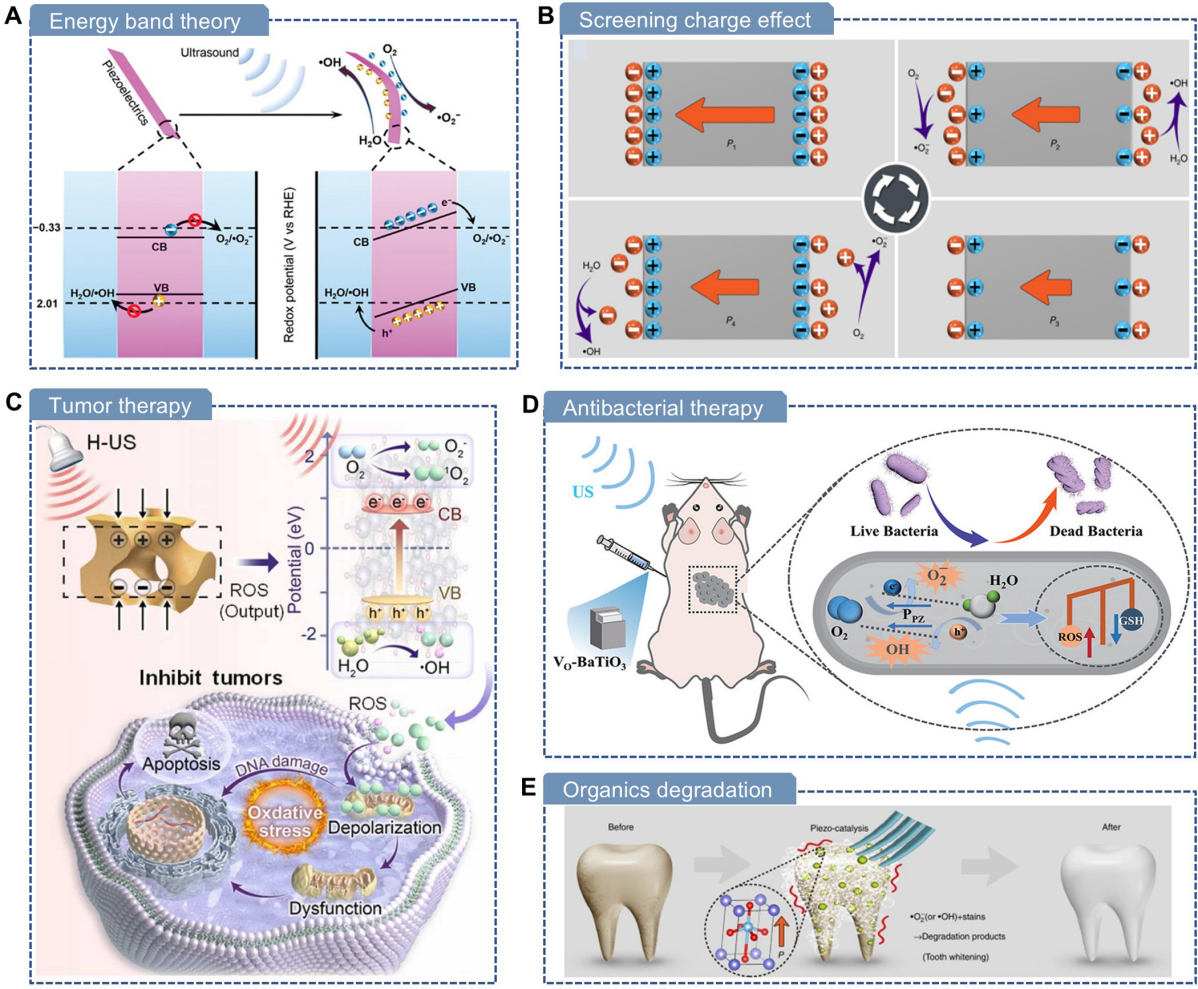
**Piezocatalytic ROS medicine.** A) Schematic of energy band theory of piezocatalytic. Reproduced with permission.^405^ Copyright 2023, Wiley-VCH. B) Schematic of screen charge theory of piezocatalytic. Reproduced with permission.^406^ Copyright 2020, Springer Nature. C) Schematic of bone cancer therapy and regeneration based on the piezoelectric lattice. Reproduced with permission.^408^ Copyright 2025, Elsevier. D) Schematic of antibacterial therapy with ROS catalyzed by BaTiO_3_ nanoparticles. Reproduced with permission.^410^ Copyright 2023, American Chemical Society. E) Schematic of pigment molecule degradation on a tooth with piezoelectric particles. Reproduced with permission.^406^ Copyright 2020, Springer Nature.

#### 12.2.2 Tumor Therapy

Cancer therapy is one of the most intensively studied applications of piezocatalysis. A major advantage is that ultrasound-driven piezocatalysis bypasses the depth limitations of photodynamic therapy and can function effectively in hypoxic tumor microenvironments where oxygen-dependent therapies underperform. Ultrasound-activated piezoelectric nanoparticles produce ROS directly within tumors, amplifying oxidative stress to trigger apoptosis or necrosis. Moreover, piezocatalytic therapy can synergize with immune checkpoint blockade or drug delivery to achieve systemic antitumor effects.

Xue and co-workers developed a wearable ultrasound microneedle patch (wf-UMP) that integrates lead-free piezoelectric nanoparticles into dissolvable microneedles and a flexible ultrasound transducer array.^407^ Upon skin application and ultrasound stimulation, the device enabled localized ROS generation in tumors, induced immunogenic cell death, and synergized with anti-PD-1 therapy to inhibit both primary and metastatic tumor growth. Chen et al. engineered biocompatible BCZT piezoelectric lattices through advanced 3D printing, endowing the constructs with bone-mimetic mechanical integrity alongside ultrasound-responsive ROS generation (Figure 18C).^408^ Such multifunctional scaffolds not only suppressed tumor progression but also enhanced osteogenic differentiation, underscoring their promise as an integrated platform for bone cancer therapy and regeneration. In addition, Zhu et al. demonstrated that tetragonal BTO nanoparticles embedded within an injectable thermosensitive hydrogel could act as efficient piezocatalysts under ultrasound, continuously generating •OH and •O_2_^–^ radicals for localized tumor eradication.^409^

Together, these examples show that piezocatalysis offers a minimally invasive, wireless, and highly controllable therapeutic strategy. By selecting appropriate piezoelectric materials and device architectures, it is possible to integrate tumor ablation with immunomodulation and tissue regeneration.

#### 12.2.3 Antibacterial Therapy

Beyond oncology, piezocatalysis has shown great promise in combating bacterial infections, particularly under the increasing threat of antibiotic resistance. By generation of ROS under ultrasound, piezoelectric materials can disrupt bacterial membranes, eradicate biofilms, and interfere with metabolism without inducing resistance.

He et al. designed oxygen-vacancy-engineered BTO nanoparticles that produced abundant ROS under ultrasound, enabling rapid sterilization of *E. coli* and *S. aureus* (Figure 18D).^410^ In vivo, these nanocrystals also accelerated wound healing, demonstrating a combination of antibacterial and regenerative effects. Zhang and colleagues reported a sonosensitive diphenylalanine-based antimicrobial peptide (FFRK8), which became piezoelectric through the FF motif.^411^ Under ultrasound, it generated ROS and killed >99% of methicillin-resistant *S. aureus* (MRSA) within 15 min, while showing excellent biocompatibility in a goat infection model. Overall, current findings support the view that piezocatalysis is a promising non-antibiotic, ROS-driven antibacterial strategy with broad-spectrum efficacy. By integrating into wound dressings or scaffolds, piezocatalytic systems not only eradicate pathogens but also support tissue repair, making them attractive candidates for infection control in drug-resistant and chronic wounds.

#### 12.2.4 Organics Degradation

Another important dimension of piezocatalysis lies in biomedical hygiene and environmental decontamination, where ROS are harnessed to oxidize and decompose organic molecules. A representative example is the nondestructive tooth whitening strategy based on BTO nanoparticles (Figure 18E).^406,412^ Under ultrasonic agitation mimicking daily brushing, the nanoparticles catalytically generated ROS that degraded pigment molecules from tea, coffee, and wine stains. Unlike peroxide bleaching, this process caused minimal enamel damage and avoided cytotoxicity, demonstrating a safe approach to cosmetic dentistry. Similarly, piezocatalysis has been applied to water purification. A ZnO/SrTiO_3_ heterojunction catalyst was engineered to facilitate interfacial charge separation, thereby enhancing hydroxyl radical generation for efficient degradation of organic pollutants such as dyes.^413^ Such results highlight how the same fundamental mechanism — mechanically induced ROS generation — can be tailored for both clinical hygiene (tooth care and wound cleaning) and environmental remediation.

### 12.3 Piezocatalytic Materials Synthesis

Piezocatalysis has recently emerged as a powerful approach for mechanochemical materials synthesis, where mechanical energy is converted into chemical potential to drive redox reactions, bond formation, and phase transformations. Unlike photocatalysis or electrocatalysis, this strategy does not rely on light, electrodes, or high temperatures, offering a sustainable route to chemical conversion. Strain-induced polarization in piezoelectric materials can activate small molecules, initiate polymerization, and mediate mineral deposition, enabling direct synthesis from mechanical input.

#### 12.3.1 Gas Evolution and Fixation

Mechanochemical piezocatalysis has attracted increasing attention for gas-evolving reactions, notably water splitting and the fixation of small molecules such as N_2_ and CO_2_. Strain-induced piezopotentials in piezoelectric and ferroelectric materials create surface charges that can directly catalyze redox reactions, offering an electrode-free and sustainable route for energy conversion.

Hydrogen and oxygen evolution: Konishi and co-workers first demonstrated that vibrating ZnO microfibers and BTO dendrites in aqueous solution could split water into H_2_ and O_2_, establishing the concept of the piezoelectrochemical effect.^414^ Starr and colleagues later validated this mechanism by showing that oscillating PMN–PT cantilevers produced measurable hydrogen, with reaction rates dependent on strain amplitude and vibration frequency (Figure 19A).^415,416^ More recently, Bowen’s group reported that BTO-based catalysts exhibited dramatically enhanced piezocatalytic hydrogen evolution when operated near their Curie temperature, where polarization fluctuations maximize charge separation.^417^

**Figure 19:**
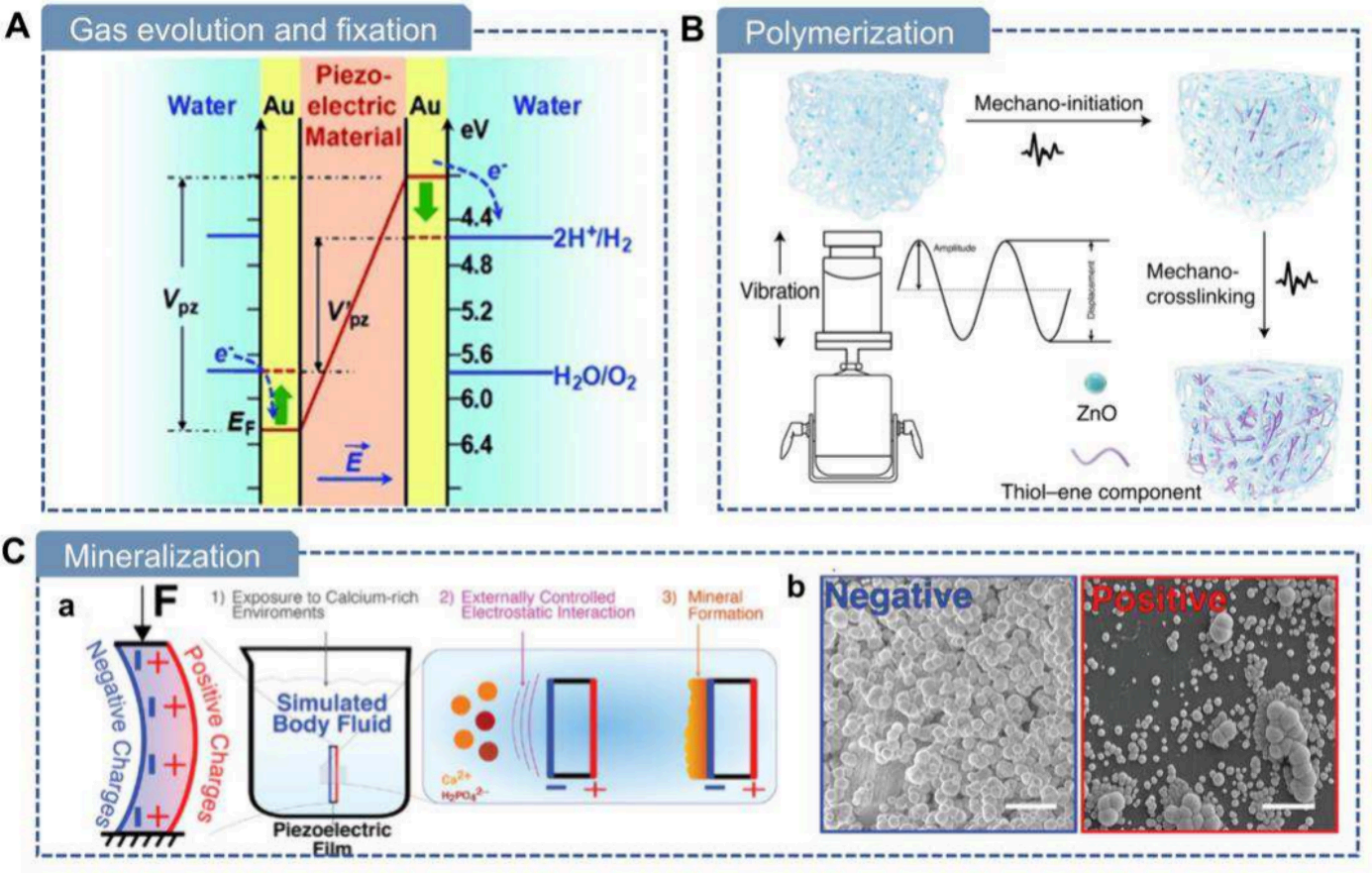
**Piezocatalytic materials synthesis.** A) Schematic of split of water with piezoelectric PMN–PT cantilevers. Reproduced with permission.^415^ Copyright 2012, Wiley-VCH. B) Schematic of thiol–ene cross-linking by midfrequency vibration of ZnO nanoparticles. Reproduced with permission.^422^ Copyright 2021, Springer Nature. C) Mineralization of piezoelectric PVDF scaffolds. (a) Schematic of the mineralization process. Negative charge generated by mechanical loads induced mineral formation. (b) Images showing mineral production on the negative side of PVDF. Reproduced with permission.^423^ Copyright 2020, Wiley-VCH.

Gas fixation: Piezocatalysis has also been extended to nitrogen and carbon dioxide conversion, which are two of the most energy-intensive chemical processes. Yuan et al. showed that oxygen-vacancy–engineered BTO significantly enhanced piezo-photocatalytic ammonia production, reaching 106.7 μmol g^–1^ h^–1^,^418^ while Ag_2_S/KTa_0_._5_Nb_0_._5_O_3_ heterojunctions promoted charge separation and improved N_2_ reduction efficiency under ultrasound.^419^ In parallel, lead-free KNN particulates enabled CO_2_ reduction without light or sacrificial agents, yielding up to 438 μmol g^–1^ h^–1^.^420,421^

In summary, piezocatalysis can efficiently drive small-molecule activation and gas production under mild conditions. From H_2_ and O_2_ evolution to N_2_ fixation and CO_2_ reduction, coupling mechanical energy with surface electrochemistry offers a sustainable route to chemical synthesis. Beyond energy applications, these reactions are also medically relevant: in situ O_2_ generation can relieve tumor hypoxia and enhance oxygen-dependent therapies, while ROS from water splitting contributes to antibacterial treatment and wound repair. Such dual functions highlight the versatility of piezocatalytic gas production.

#### 12.3.2 Polymerization

Mechanoredox polymerization employs piezocatalysis to convert mechanical energy into redox activity, enabling polymer formation under mild and sustainable conditions. Unlike conventional thermal or photochemical routes, this strategy avoids high temperatures, UV irradiation, or external initiators, making it particularly attractive for sustainable chemistry and biomedical contexts. Representative advances include atom transfer radical polymerization (ATRP),^424^ reversible addition–fragmentation chain transfer (RAFT), free radical polymerization (FRP), and thiol–ene click chemistry (thiol–ene).

ATRP: Esser-Kahn and co-workers, for the first time, demonstrated that ultrasound-excited BTO nanoparticles reduce Cu­(II) to Cu­(I), initiating ATRP of acrylates and methacrylates with controlled molecular weights and narrow dispersities.^424^ Building on this concept, Wang et al. enhanced mechanically induced ATRP by promoting interfacial electron transfer: replacing BTO with ZnO strengthened NP–Cu interactions and cutting the piezoelectric nanoparticle loading by >70%.^425^

RAFT: Chakma et al. introduced mechanoredox catalysis into RAFT by combining BTO with diaryliodonium salts. Under ultrasound or ball milling, radicals were generated efficiently, yielding polymers with predictable molecular weights and low dispersities, demonstrating that piezocatalysis can be readily adapted to controlled radical systems.^426^

FRP: Nothling and colleagues reported solvent-free FRP driven by stress or ball milling of BTO powders, where radicals from surface water initiated polymerization of acrylamides, acrylates, and styrenics.^427^ Wang et al. showed that 2D MoS_2_ nanosheets accelerated radical generation, enabling rapid hydrogel polymerization and remodeling within minutes, illustrating the potential for dynamic and adaptive materials.^428^

Thiol–ene: Mohapatra et al. reported ultrasound-driven thiol–ene cross-linking mediated by piezo-electrochemical Cu catalysis, achieving efficient step-growth processes in organic environments.^429^ Complementing this, Esser-Kahn’s group introduced a truly mechanochemically initiated thiol–ene route: midfrequency vibration (≈10^2^–10^3^ Hz) of organogels containing piezoelectric ZnO nanoparticles generates interfacial thiyl radicals and drives room-temperature, oxygen-tolerant thiol–ene cross-linking (Figure 19B).^422^ The efficiency and orthogonality of thiol–ene chemistry highlight its value for constructing functional gels and bioinspired networks.

Overall, these studies demonstrate that mechanoredox catalysis can mediate diverse polymerization strategies. By enabling polymerization processes through mechanical excitation, piezocatalysis provides a versatile and sustainable platform for advanced polymeric materials, with particular promise for biomedical uses such as injectable hydrogels, self-healing scaffolds, and stimuli-responsive drug delivery systems.

#### 12.3.3 Mineralization

Mineralization, a hallmark of biological materials such as bone and shell, has recently been emulated in synthetic systems through piezocatalytic and mechanoredox strategies. By coupling mechanical stress with piezoelectric charge generation, inorganic minerals can be deposited within polymeric matrices, leading to composites that strengthen in response to a load.

Orrego et al. designed bioinspired piezoelectric PVDF scaffolds that adaptively mineralize under mechanical stress (Figure 19C).^423^ Local piezoelectric charges guided the deposition of calcium-based minerals from the surrounding media, producing functionally graded structures that reinforced regions of high stress, mimicking bone remodeling. Similarly, Chernozem et al. fabricated electrospun poly­(3-hydroxybutyrate)-based fibrous scaffolds that underwent ultrasound-induced CaCO_3_ mineralization, resulting in improved hydrophilicity, enhanced osteoblast adhesion, and proliferation, demonstrating strong potential for bone tissue engineering.^382^ Expanding beyond scaffolds, Ayarza and colleagues reported a striking example of mechanically triggered mineralization inside organogels.^430^ Under vibration, ZnO nanoparticles reacted with thiadiazole derivatives to form Zn/S mineral microrods, selectively reinforcing the gel matrix at stressed regions. This approach provided site-specific mineral growth, resulting in composites with significantly improved mechanical strength. In short, by directing inorganic deposition through mechanical cues, piezocatalytic mineralization opens new avenues in regenerative medicine and functional composite design.

Overall, these emerging applications are still predominantly explored using traditional inorganic or nondegradable organic piezoelectric materials, while piezoelectric biomaterials face distinct challenges in achieving comparable performance and stability for translation into such advanced fields as neuromodulation, piezocatalytic medicine, and catalytic material synthesis. A primary limitation is their relatively low electromechanical output at device-relevant scales, which constrains their ability to meet the stimulation thresholds required for deep neuromodulation or to sustain efficient catalytic reactions. Their structural stability under physiological conditions is also limited: hydration, enzymatic degradation, and cyclic mechanical loading can rapidly induce fatigue and diminish the piezoelectric performance. From a structural perspective, fabricating complex architectures — ranging from nanoscale particles for catalytic therapies to macroscale scaffolds for neuromodulation — remains more challenging than in inorganic systems with mature ceramic processing routes. Finally, the intrinsic catalytic efficacy of biomolecular systems tends to be modest compared to inorganic oxides, with lower charge separation and ROS generation efficiencies, underscoring the need for innovative molecular designs or hybrid strategies that preserve both biocompatibility and biodegradability.

## 13 Summary and Perspective

Piezoelectric biomaterials hold great promise as intelligent materials for next-generation bioelectronics and biomedical applications. Their intrinsic biocompatibility and biodegradability allow safe integration into living systems, where they can naturally degrade or be absorbed after fulfilling their function. A key step toward practical implementation is the ability to self-assemble or microfabricate these materials into large-scale, highly ordered, and robust structures. In this comprehensive review, we critically summarize recent advances in the design strategies and fabrication techniques of piezoelectric biomaterials. We compare the piezoelectric properties of amino acids, peptides, proteins, polysaccharides, and synthetic biomaterials, with a focus on their processing methods, structural forms, and characterization techniques, as detailed in Tables 2–5. Additionally, we highlight emerging biomedical applications, including sensing, actuation, energy harvesting, filtration, tissue engineering, neuromodulation, piezocatalytic medicine, and piezocatalytic materials synthesis. Despite increasing research interest, the development of reliable, biodegradable piezoelectric biomaterials remains in its early stages, facing key challenges on the path to widespread clinical and commercial adoption, as outlined in Figure 20.

**Figure 20:**
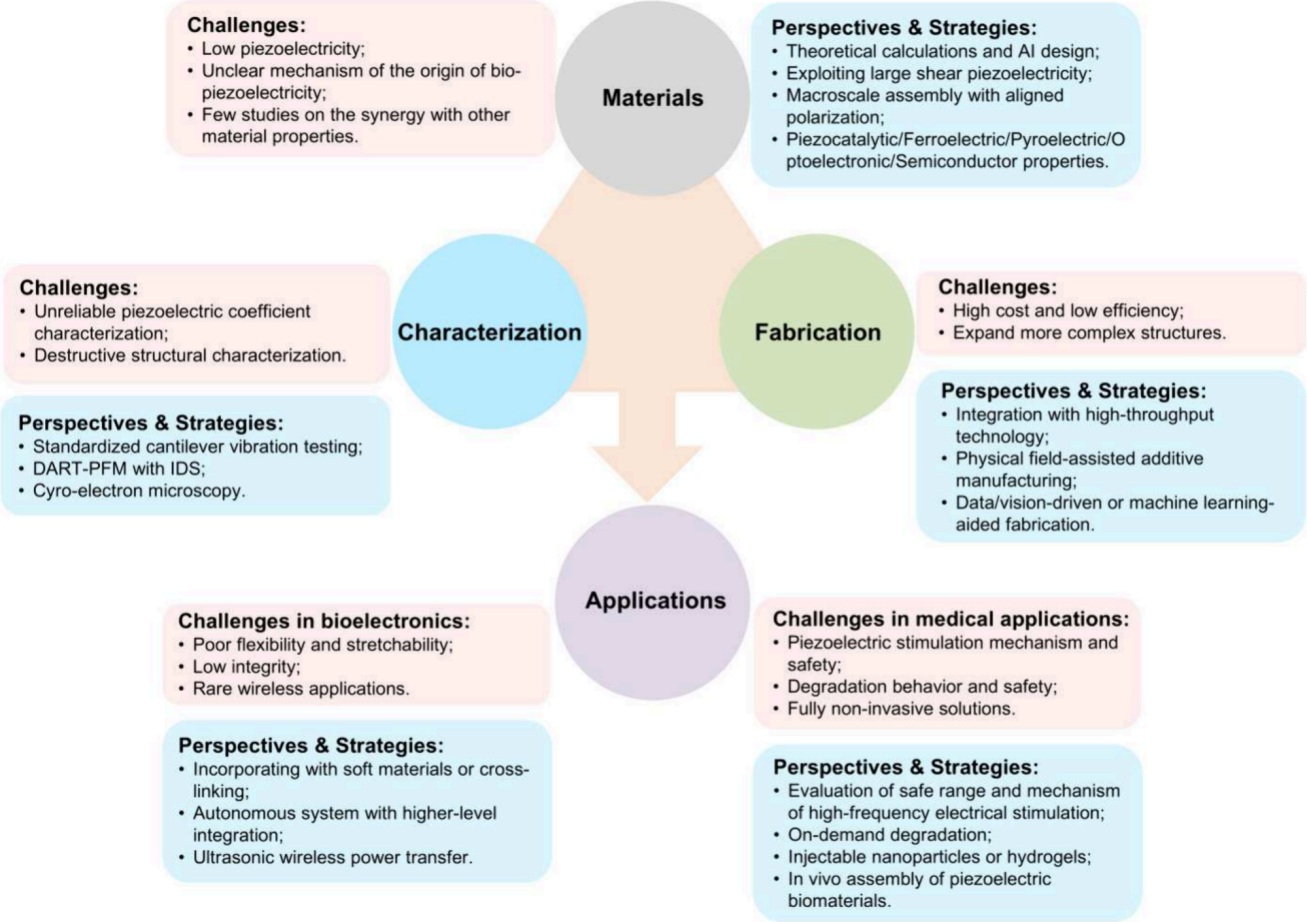
Challenges and perspectives.

**Table 2:** Comparison of the Fabrication Method and Piezoelectric Coefficients of Piezoelectric Amino Acids and Their Derivatives

Type	Materials	Morphology	Fabrication Method	Piezoelectric Coefficients (pm V^–1^)	Measurement/Calculation Method	Ref
Amino acids and their derivatives	β-glycine	Needle-like crystals	Drop casting	178 (d_16_)	DFT calculations and resonance testing	^42^
	β-glycine	Nanocrystalline films	Electrospray deposition	11.2 (d_33_)	PFM and quasi-static method	^53^
	β-glycine	Isolated crystals	Inkjet printing	2–4 (d_33_)	PFM	^271^
	β-glycine-PVP	Nanocomposite films	Aerosol printing	10.8 (d_33_)	PFM and quasi-static method	^308^
	β-glycine-PVA	Nanofibers	Electrospinning	4.2 (d_eff_)	PFM	^301^
	γ-glycine	Single crystals	Drop casting	10.4 (d_33_)	DFT calculations and quasi-static method	^42^
	γ-glycine-PVA	Sandwich films	Solution casting (Hydrogen bonding heterostructure)	5.3 (d_33_)	Quasi-static method	^74^
	dl-alanine	Single crystals	Drop casting (Racemic coassembly)	10.3 (d_33_)	DFT calculations and PFM	^54^
	dl-alanine	Polycrystalline aggregate films	Drop casting (Racemic coassembly)	4.1 (d_33_)	Quasi-static method	^54^
	Isoleucine	Polycrystalline aggregate films	Mechanical annealing	1.2 (d_33_)	Quasi-static method	^252^
	Valine	Sheet arrays	PVD	11.4 (d_33_)	DFT calculations and PFM	^329^
	L-AcW	Single crystals	Drop casting (Molecular modifications)	47.3 (d_25_)	DFT calculations	^63^
	BPA/Ac-l-Ala	Single crystals	Drop casting (Co-assembly)	26.3 (d_14_)	DFT calculations	^62^
	BPA/Ac-d-Ala	Single crystals	Drop casting (Co-assembly)	21.9 (d_14_)	DFT calculations	^62^

**3:** Comparison of the Fabrication Method and Piezoelectric Coefficients of Piezoelectric Peptides and Their Derivatives

Type	Materials	Morphology	Fabrication Method	Piezoelectric Coefficients (pm V^–1^)	Measurement/Calculation Method	Ref
Peptides and their derivatives	FF	Nanotubes	Drop casting	18 (d_33_)	PFM	^103^
				80 (d_15_)		
	FF	Vertical aligned microrod array	Epitaxial growth with electric filed	17.9 (d_33_)	PFM	^310^
	FF	Horizontally aligned nanotubes	Dip-coating	46.6 (d_15_)	PFM	^254^
	FF	3D printed pattern	3D printing	7.23 (d_33_)	PFM	^253^
	FF	Microribbons	Inkjet printing	40 (d_33_)	PFM	^270^
	L,L-FF-D,D-FF	2D layered crystals	Racemic coassembly and mechanical exfoliation	20 (d_33_)	PFM	^236^
	20% FW-FF	Fibers	Drop casting (Co-assembly)	35.5 (d_33_)	PFM	^237^
	Fmoc-FF	Nanofiber network hydrogels	Solvent-based method (Molecular modifications)	1.7 (d_15_)	PFM	^87^
	Hyp-Phe-Phe	Collagen-like fibers	Drop casting (Molecular modifications)	16 (d_34_)	DFT calculations and PFM	^234^
				4 (d_33_)		
	cyclo-GW	Needle-like crystals	Drop casting (Molecular modifications)	–0.2 (d_22_)	DFT calculations	^89^
				14.1 (d36)		
	Boc-Dip-Dip	Nanorod-like crystals	Drop casting (Molecular modifications)	73.1 (d_33_)	DFT calculations and PFM	^223^
	L-tryptophan-d-tryptophan	Layered organic-water structure	Drop casting (Hydrogen bonding heterostructure and neutron doping)	61.9 (d_33_)	DFT calculations and PFM	^241^
	PBLG	Nanofibers	Electrospinning	25 (d_33_)	Quasi-static method	^95^
	PBLG	Films	Solution casting with magnetic field	26 (d_14_)	Quasi-static method	^97^
	PBLG	Films	Polymerization with electric field	23 (d_33_)	Quasi-static method	^96^
	PMLG	Films	Solution casting	2 (d_14_)	Quasi-static method	^91^

**4:** Comparison of the Fabrication Method and Piezoelectric Coefficients of Piezoelectric Proteins and Formed Biological Tissues

Type	Materials	Morphology	Fabrication Method	Piezoelectric Coefficients (pm V^–1^)	Measurement/Calculation Method	Ref
Protein	Collagen (SIS)	Utrathin films (100 nm)	Mechanical exfoliation	4.1 (d_15_)	PFM	^152^
	Collagen (Bone)	Bulk	Mechanical cutting	0.2 (d_14_)	Quasi-static method	^16^
	Collagen (Rat tail tendon)	Films	Mechanical cutting	6.21(d_15_)	PFM	^149^
				0.89 (d_33_)		
	Collagen (Human tendon)	Films	Mechanical cutting	1 (d_15_)	PFM	^111^
	Silk	Films	Mechanical annealing	1.5 (d_14_)	Quasi-static method	^117^
	Spider silk	Fibers	Mechanical cutting	0.36 (d_33_)	PFM	^116^
	Keratin (Wool)	Fibers	Mechanical cutting	0.1 (d_14_)	Quasi-static method	^28^
	Keratin (Horn)	Bulk	Mechanical cutting	0.6 (d_14_)	Quasi-static method	^120^
	Elastin (aortic wall)	Films	Mechanical cutting	1 (d_33_)	PFM	^128^
	Lyzozyme	Aggregate films	Drop casting	6.5 (d_33_)	Quasi-static method	^132^
	Phage virus	Monolayer films	Dip-coating and genetic modifications	0.7 (d_33_)	PFM	^21^
	Phage virus	Vertical aligned nanopillars	Template confinement and genetic modifications	10.4 (d_33_)	PFM	^133^
	Phage virus	Vertical aligned films	Template confinement and genetic modifications	13.2 (d_33_)	PFM	^134^

**Table 5:** Comparison of the Fabrication Method and Piezoelectric Coefficients of Piezoelectric Cellulose, Chitin, Synthetic Biomaterials, and Their Derivatives

Type	Materials	Morphology	Fabrication Method	Piezoelectric Coefficients (pm V^–1^)	Measurement/Calculation Method	Ref
Cellulose	Wood	Bulk	Mechanical cutting	0.1 (d_14_)	Quasi-static method	^14^
	Cellulose microfiber	Microfiber paper	Hydrothermal synthesis	0.4 (d_33_)	Quasi-static method	^186^
	CNC	2D films	/	1.3 (d_22_)	DFT calculations	^185^
				0.9 (d_11_)		
	CNC	Films	Corona-poled	2.31 (d_33_)	PFM	^311^
CNC	Vertically aligned films	Template confinement with electric field	19.3 (d_33_)	PFM	^184^	
Chitin	α-Chitin	Film	/	0.1 (d_14_)	Quasi-static method	^190^
	β-Chitin	Film	Centrifugal casting	4 (d_33_)	DFT calculations and PFM	^57^
Synthetic biomaterials	PLLA	Film	Mechanical annealing	11 (d_14_)	Quasi-static method	^191^
	PLLA	Nanofiber	Electrospinning	19 (d_14_)	Quasi-static method	^192^
	PHB	Films	Solution casting	1.6–2 (d_14_)	Quasi-static method	^204^
	2,2,3,3,4,4-Hexafluoropentane-1,5-diol (HFPD)	Single crystal	Solvent diffusion method	138 (d_33_)	Quasi-static method and PFM	^207^
	HFPD-PVA	Films	Solution casting	34.3 (d_33_)	Quasi-static method	^207^

### 13.1 Materials

#### 13.1.1 Challenges

Currently, the performance of piezoelectric biomaterials, particularly the piezoelectric strain coefficient (d_33_), remains relatively low compared with inorganic ceramics or organic polymers. For applications not requiring high charge output, such as sensors or piezoelectric stimulation, piezoelectric biomaterials might be sufficient to fulfill their functionalities. However, for transducers and energy harvesters, it is still essential to enhance the piezoelectric properties of these biomaterials to achieve more reliable functionality and effectiveness. Furthermore, the underlying mechanisms responsible for the piezoelectric properties of biomaterials are still not clearly understood. Unlike inorganic piezoelectric crystals, the polarization in biomaterials typically arises from molecular dipoles, and their organization and arrangement are considerably complex. Additionally, there has been limited research focusing on the investigation of other chemical properties beyond piezoelectricity and their synergistic interactions with piezoelectricity.

#### 13.1.2 Perspectives and Strategies

Establishing reliable correlations of the molecular structure and chemical characteristics of biomaterials with their piezoelectric properties is of paramount importance for understanding and improving their piezoelectricity. The advancement of theoretical research has been greatly facilitated by the development of theoretical calculations and simulations, such as DFT and MD. These approaches provide valuable insights for materials design, significantly accelerating the exploration of piezoelectric biomaterials. Notably, utilizing emerging machine learning AI techniques for materials discovery will offer promising avenues for rapid advancements in this field.

Furthermore, despite many biomaterials exhibiting lower longitudinal piezoelectric coefficients, they often possess higher shear piezoelectric coefficients. Therefore, there is promising potential to achieve excellent output performance by effectively harnessing their shear piezoelectricity via the rational design of precisely intricate 3D structures. In addition, controlling the molecular polarization orientation on a large scale is a crucial step in enhancing the macroscopic piezoelectricity of biomaterials.

Besides piezoelectricity, many of these biomaterials also exhibit other material properties, such as ferroelectricity, pyroelectricity, optoelectronic properties, and semiconductor properties.^88,431–433^ The synergy of piezoelectricity with these different characteristics in biomaterials will open up new possibilities.^434,435^ For instance, developing ferroelectric biomaterials with low coercive fields would overcome the challenges of random orientation, enabling them to achieve significant macroscopic piezoelectricity through electric poling post-treatment, similar to traditional inorganic piezoceramics.

### 13.2 Characterization

#### 13.2.1 Challenges

The three most employed tests in piezoelectric biomaterials research are PFM, a quasi-static d_33_ meter, and device testing under tapping force. However, each of these techniques has its limitations. PFM is capable of measuring only the nanoscale response and is susceptible to interference from surface features such as electrostatic charges and large roughness.^436,437^ The d_33_ meter faces challenges when testing thin films, soft materials, and materials with weak piezoelectricity. Device testing under tapping force involves an undefined force and the introduction of triboelectricity.^359,361^ Moreover, many piezoelectric tests lack standardization, as the rate of applied stress or compressed area is often not reported. Additionally, voltage or current outputs are frequently reported without normalization to the respective area or thickness.

In addition to the quantification of piezoelectricity, structural characterization of piezoelectric biomaterials also faces challenges. Because of their relatively fragile chemistry, their lattice structures can be easily damaged by high-energy electron beams. As a result, the currently available characterization techniques are unable to provide high-quality lattice images of biomaterials, thus impeding further research on establishing reliable correlations between their piezoelectric properties and structural characteristics.

#### 13.2.2 Perspectives and Strategies

Standardized piezoelectric testing and a reliable comparison database are essential for advancing research in piezoelectric biomaterials. The cantilever energy harvester has been widely adopted as a performance benchmark.^41,351^ Typically, a single piezoelectric element is placed near the fixed, high-stress end of the cantilever. Once the cantilever’s material and geometry are defined, the load conditions can be standardized, simplifying the quantification of piezoelectric output and coefficients. This method also avoids triboelectric interference and ensures consistency in testing. We thus strongly recommend vibration testing as a standard approach for characterizing piezoelectric biomaterials. Here, we propose a set of standardized testing parameters, including the elastic modulus, dimensions, initial displacement, and electrode distribution and dimensions of the cantilever, as summarized in Table S1 and Figure S1. By measuring the material’s modulus, capacitance, and electrical output, researchers can calculate a reliable effective d_33_ value using eq 2 (details shown in Supplementary Note 1).

2 $$d_{33}\approx 2d_{31}\approx \frac{8UCL^{3}}{3Ehy{L_{1}}^{2}\left(2L-L_{1}\right)}\approx 1.34\times 10^{7}\frac{UC}{E}$$

PFM remains a powerful, nondestructive technique capable of detecting picometer-scale deformations with nanometer spatial resolution.^437–439^ To minimize surface topography effects, the dual AC resonance tracking (DART) method can be used to obtain intrinsic piezoresponses by correcting resonance magnification with the quality factor.^440^ To reduce false signals from electrostatic surface charges, researchers may use probes less sensitive to electrostatic forces (e.g., stiffer or longer-tipped probes) or apply an external DC bias, as measured in scanning Kelvin probe microscopy (SKPM), to compensate for probe–sample potential differences. Notably, the recently developed Interferometric Displacement Sensor (IDS) technique enables absolute measurement of cantilever deflection and amplitude, effectively eliminating electrostatic interference in piezoelectric testing.^441,442^

For structural characterization, cryo-electron microscopy (cryo-EM) holds great promise as a nondestructive tool for studying piezoelectric biomaterials. By rapidly freezing the sample, cryo-EM vitrifies it into a glass-like state without forming ice crystals. It protects biological samples from the detrimental effects of high-vacuum conditions and intense electron beams. This advanced technique is expected to provide valuable insights into the mechanisms of piezoelectric biomaterials in the near future.

### 13.3 Fabrication

#### 13.3.1 Challenges

Despite extensive research on microfabrication techniques for piezoelectric biomaterials in recent years, several challenges still need to be addressed. Specifically, the current manufacturing technologies often suffer from high costs and low efficiency, making it difficult to transition them to practical industrial production. In addition, most fabrication routes for biomaterials are inherently incompatible with standard electronic manufacturing processes, which limits their integration into existing device platforms. Furthermore, precise control over the assembly of these biomaterial structures remains a significant challenge, hindering the expansion toward more well-arranged and complex architectures. Moreover, while microfabricated structures might exhibit enhanced physical properties, the underlying mechanisms behind these improvements require further comprehensive investigation.

#### 13.3.2 Perspectives and Strategies

Current manufacturing technologies can be integrated with high-throughput approaches, such as roll-to-roll platforms, to enable efficient large-scale production of piezoelectric biomaterials.^308^ In addition, combining additive manufacturing techniques with various physical field assistance offers the capability to fabricate more sophisticated structures such as biomimetic architectures and 3D topological designs, where in situ physical fields can promote the favorable alignment of piezoelectric domains.^443–450^ For instance, electric field-assisted 3D printing has been employed to construct one-dimensional peptide assemblies mimicking the multilayered architecture of lobster exoskeletons.^85^ Additionally, electrospray deposition can be used to manufacture freestanding nanoparticles or well-defined micropatterns.^292^ Furthermore, sound and light may also play unexpected roles in the fabrication of biomaterials. Finite element analysis can guide the design of 3D structures for piezoelectric biomaterials, with the aim of achieving optimal performance. Moreover, data-/vision-driven or machine learning-assisted advanced manufacturing approaches hold the potential to explore more possibilities for the fabrication of biomaterials.^451^

### 13.4 Bioelectronics

#### 13.4.1 Challenges

Soft bioelectronics must possess sufficient elasticity and stretchability to conform to biological tissues and to accommodate substantial and frequent strains caused by body movements. However, currently studied piezoelectric biomaterials often exhibit high Young’s modulus and poor elastic recovery, making them incompatible with soft bioelectronics. Furthermore, most developed piezoelectric biomaterials only achieve basic functionalities through combination with biodegradable electrodes and encapsulation materials, while there is limited progress in advancing the higher-level integration of bioelectronic devices. Moreover, currently available piezoelectric biomaterial-based electronic devices are rarely fully wireless. Biodegradable conductive materials have been used as transdermal wires to connect implantable bioelectronic devices to external devices for data collection or control purposes. Nevertheless, the risk of infection persists at the interface between the skin and the electrical wires.

#### 13.4.2 Perspectives and Strategies

Enhancing the stretchability of electronic materials can be achieved by modifying the structure of rigid materials using mechanical and geometrical designs or incorporating piezoelectric nanoparticles into elastomers. Cross-linking was recently reported to treat piezoelectric polymers, enabling high elastic strain while maintaining favorable piezoelectric properties. Precise control over slight cross-linking holds the potential to design inherently stretchable piezoelectric biomaterials, achieving a delicate balance between crystallinity and elasticity.^242^

By integration of piezoelectric biomaterials that actuate, sense, and harvest energy with a biocompatible microchip, circuits, and capacitors, a self-contained system can be created, operating autonomously without the need for an external interface. For example, implantable bioelectronic devices based on piezoelectric biomaterials offer the potential to replace defective muscles. The piezoelectric sensors can recognize muscle dysfunction, which can be corrected through a piezoelectric actuator. Simultaneously, the changes can be monitored and signals can be sent to the implanted microchip, which controls the entire process. The energy required for the system’s normal functioning can be harvested from body movements or vibrations using piezoelectric nanogenerators and stored in a biocompatible capacitor via a rectifier circuit.

Achieving reliable wireless transmission is crucial for transient bioelectronic devices to eliminate the risk of infection at wired connection sites and enable portable applications. Wireless transmission of power and sensing signals can be achieved through various methods, including electromagnetic (EM) waves, near-field inductive coupling (NIC), and acoustic waves.^452^ However, wireless power transfer based on EM waves and NIC faces challenges related to tissue penetration depth limitations and difficulties in miniaturization. For sub-millimeter RF-powered devices, the effective frequencies typically lie in the GHz range, where the human body readily absorbs EM radiation. For NIC wireless power transfer, reducing the size of the receiver coils results in a decrease in the output power. Moreover, such systems become more susceptible to disturbances caused by variations in the distance or angle between the transmitter and receiver. Ultrasound provides an effective method for wireless power transmission since its wavelength is approximately 10^5^ times smaller than electromagnetic waves of the same frequency, which allows the miniaturization of piezoelectric energy harvesting devices. Therefore, ultrasonic energy harvesters using piezoelectric biomaterials hold potential for achieving fully degradable and completely wireless transient bioelectronic devices.

### 13.5 Medical Applications

#### 13.5.1 Challenges

While the use of piezoelectric implants for electrical stimulation therapy to promote cell growth, differentiation, and proliferation has been widely reported, the safety range of piezoelectric stimulation remains unclear. Additionally, due to the inherent low-pass filtering properties of cells, low-frequency electrical stimulation (below 500 Hz) is typically employed for neural activation, while high-frequency stimulation is used for conduction block to alleviate pain.^394,452^ Currently, most applications of piezoelectric stimulation therapy rely on high-frequency ultrasound (≥20 kHz) triggers, which may be too fast to stimulate the neural activity directly.

Further, due to complex degradation kinetics and the intricate physiological environment within the body, predicting the degradation process of implants is challenging. However, biocompatible elements must be released within specific doses and rates to ensure safety. Achieving on-demand degradation of piezoelectric biomaterials, which can dissolve at controlled rates or completely/partly dissolve at a triggered time, remains a significant challenge.

Moreover, while piezoelectric biomaterials can safely degrade within the body, their implantation still requires invasive surgical procedures, leading to complications such as damage to healthy tissues, infections, inflammation, and longer recovery times. Therefore, there is a pressing need to develop less invasive techniques and, ideally, approaches that eliminate the need for surgical intervention.

#### 13.5.2 Perspectives and Strategies

The safety of piezoelectric stimulation in various cells and tissues requires a comprehensive evaluation and thorough exploration. Furthermore, a clear understanding of how the parameters of the piezoelectric biomaterials, including the piezoelectricity, material morphology, applied stress, and surface charge, impact piezoelectric stimulation is essential to achieve optimal therapeutic outcomes. Moreover, although some studies have proposed explanations through a sequence of events involving an elevated calcium influx,^453^ the fundamental mechanisms of action behind the efficacy of high-frequency stimulation should be further investigated and clearly understood. Additionally, specific techniques, such as employing additional circuit rectification or leveraging the self-rectifying behavior of materials, could enable the utilization of high-frequency stimulation to activate voltage-gated ion channels effectively.^454^

The most common approach to regulating the degradation rate is achieved by adjusting the thickness of biodegradable encapsulation materials such as PLA.^191^ Materials capable of initiating degradation processes on-demand and with controllable rates are crucial for the safe application of temporary biomedical implants. Therefore, it is necessary to develop new piezoelectric biomaterials and study their mechanisms in response to various physicochemical stimuli, including exposure to solvents, light, heat, electricity, and sound.^453,455–457^ Furthermore, the biocompatibility assessment of their degradation processes requires further research and extension to larger mammalian models.

To avoid invasive surgeries, nanoparticles or hydrogels offer a viable solution that can be noninvasively injected into the body and remotely activated using external ultrasound devices. While inorganic nanoparticles like BTO have been widely employed in tissue regeneration, neural modulation, and cancer treatment,^10^ reliable manufacturing of biodegradable piezoelectric nanoparticles is yet to be achieved. Potential techniques, such as electrospray deposition to create freestanding piezoelectric biomaterial nanoparticles, could be explored.^53^ Additionally, the recent advancements in utilizing focused ultrasound and sono-ink for printing at centimeter depths through biological tissues have garnered significant interest.^458,459^ In the future, similar approaches may enable noninvasive printing of piezoelectric biomaterials inside the body, allowing complex structures to form through in situ self-assembly.

In summary, piezoelectric biomaterials stand at the frontier of biointegrated electronics and biomedicine, offering biodegradable, biocompatible, sustainable, and functionally versatile alternatives to conventional materials. While significant strides have been made in materials discovery, fabrication techniques, and proof-of-concept applications, the transition from laboratory research to real-world implementation remains limited by several critical challenges. These include enhancing piezoelectric performance, achieving scalable and standardized manufacturing, integrating wireless and self-sustained systems that interface seamlessly with biological environments, and ensuring safe operation and controllable degradation under physiological conditions.

Looking ahead, a truly transformative leap will depend on interdisciplinary collaboration, bridging materials science, chemistry, physics, biomedical engineering, biotechnology, electronics, and computational modeling. Emerging technologies such as AI-guided materials design, high-resolution additive manufacturing, and noninvasive in situ assembly may unlock new levels of precision, functionality, and personalization. With sustained effort and innovation, piezoelectric biomaterials are poised to redefine the landscape of future biological devices, enabling smarter, safer, and more sustainable medical interventions.

## Supplementary Material



## Notes

Calculations and schematic and standardized parameters of cantilever vibration testing (PDF)
