# Supplementary material for: Design and Manufacturing of Piezoelectric Biomaterials for Bioelectronics and Biomedical Applications
Source: Chem Rev. 2025 Oct 9;125(20):9875–929. doi: 10.1021/acs.chemrev.5c00399 (PMC12550821; doi:10.1021/acs.chemrev.5c00399)
Supplement: Supplementary file 1 [file cr5c00399_si_001.pdf]

Supplementary Information for

Design and manufacturing of piezoelectric biomaterials for bioelectronics and biomedical applications

Authors

Zhuomin Zhang[1,2,3†*], Zhenqi Wang[1†], Xuemu Li[1], Yi Zheng[1,2], Zhengbao Yang[1*]

Affiliations

1. Department of Mechanical and Aerospace Engineering, Hong Kong University of Science and Technology, Clear Water Bay, Hong Kong SAR 999077, China
2. Department of Mechanical Engineering, City University of Hong Kong, Hong Kong SAR 999077, China
3. Department of Mechanical Engineering, Stanford University, Stanford, CA 94305, USA

* Correspondence: Zhengbao Yang, zbyang@ust.hk; Zhuomin Zhang, zmzhang@stanford.edu

† These authors contributed equally to this work

This PDF file includes:

Supplementary Note 1
Figure S1
Table S1

Supplementary Note

To quantify the piezoelectric performance of piezoelectric biomaterials, it is recommended to evaluate their d_{33} coefficients using a standardized cantilever energy harvester-based vibration system. The measurement system is depicted in Supplementary Figure 1. For the preparation of the biomaterial thin film PEH, silver paste or sputtering deposition should be employed to apply electrodes on both sides of the film. The electrode area is 1 cm x 1 cm, and the film area should be slightly larger to prevent electrical contact between the two electrodes. Subsequently, the film is wired, encapsulated, and adhered onto the fixed side of a rectangular-shaped stainless-steel cantilever with length L (10 cm), width b (2 cm), and thickness h (350 μm) using an AB glue. In some cases, biomaterials can be directly deposited or grown on a stainless-steel substrate, which can serve as the bottom electrode, eliminating the need for adhesives. Typically, the elastic modulus of biomaterials ranges from megapascals to gigapascals, while stainless steel has an elastic modulus (E_s) of 200 GPa, significantly higher than that of biomaterials. This facilitates efficient stress transfer to the biomaterial thin films.

When a spontaneous force F is applied to the free end of the cantilever, it vibrates, and the stress along the length direction at different positions can be calculated based on the vertical displacement y of the free end, which is recorded by a laser vibrometer. The spontaneous force F can be calculated by

$$F = \frac{3E_s I y}{L^3} \qquad (1)$$

Where I is the moment of inertia of the cantilever. Assuming the bending moment of the cantilever is M, the average stress of the substrate surface in the region of PEH along the length direction is denoted as σ_s, which can be determined by

$$\sigma_S = \frac{M\frac{h}{2}}{I} = \frac{F\left(L-\frac{L1}{2}\right)\frac{h}{2}}{I} = \frac{3E_s hy(2L-L_1)}{4L^3} \qquad (2)$$

Considering that the strain of the substrate and the film are similar, we can calculate the average stress of the film along the length direction based on the following equation.

$$\sigma_1 = \frac{E}{E_s}\sigma_S = \frac{3Ehy(2L-L_1)}{4L^3} \qquad (3)$$

Where, E represents the elastic modulus of the piezoelectric biomaterials thin film. The cantilever PEH typically employs the d_{31} mode, activated by a transverse or bending stress and generating the charge on the out-of-plane surface. Hence, the transverse piezoelectric coefficient can be derived from

$$d_{31} = \frac{Q_3}{\sigma_1 A} = \frac{UC}{\sigma_1 A} \qquad (4)$$

where Q denotes the generated charge and A represents the area of the electrodes. U represents the voltage output of the PEH, and C is the capacitance of the device. Generally, d_{33} is approximately twice the value of d_{31}; therefore, we can obtain the value of d_{33} from

$$d_{33} \approx 2d_{31} = \frac{2Q}{\sigma_{11}A} = \frac{8UCL^3}{3EhyL_1{}^2(2L-L_1)} \qquad (5)$$

It should be noted that the d_{33} quantification approach proposed here provides an approximate estimation of the piezoelectric performance for biomaterials thin films. For thicker or bulk materials, the method may require further modifications to account for their specific characteristics.

Supplementary Figure

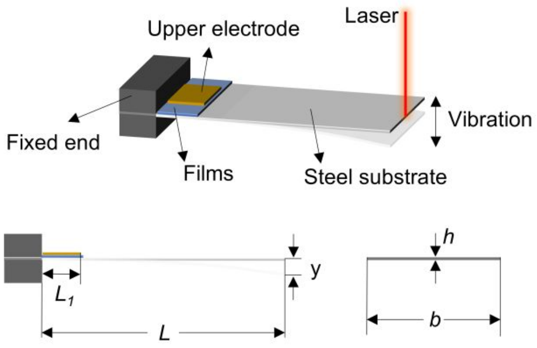

Figure S1. Schematic of standardized cantilever vibration testing.

Supplementary Table

Table S1. Standardized parameters for the cantilever vibration testing.

Type	Parameters	Values
Standardized cantilever vibration testing system	Elastic Modulus of the substrate E_S (GPa)	200
	Length L (cm)	10
	Width b (cm)	2
	Thickness h (μm)	350
	Vertical displacement y (cm)	3
	Top electrode length and width L_1 (cm)	1
To be measured	Elastic Modulus of the piezoelectric film E (GPa)	Measured value
	Capacitance C (F)	Measured value
	Voltage output U (V)	Measured value
